# Cell coupling compensates for changes in single-cell Her6 dynamics and provides phenotypic robustness

**DOI:** 10.1242/dev.202640

**Published:** 2024-05-20

**Authors:** Parnian Doostdar, Joshua Hawley, Kunal Chopra, Elli Marinopoulou, Robert Lea, Kiana Arashvand, Veronica Biga, Nancy Papalopulu, Ximena Soto

**Affiliations:** ^1^ Division of Developmental Biology and Medicine, School of Medical Sciences, Faculty of Biology, Medicine and Health, The University of Manchester, Oxford Road, Manchester M13 9PT, UK; ^2^ Division of Molecular and Cellular Function, School of Biological Sciences, Faculty of Biology, Medicine and Health, The University of Manchester, Oxford Road, Manchester M13 9PT, UK

**Keywords:** Zebrafish, Telencephalon, Her6, Oscillations, Neurogenesis

## Abstract

This paper investigates the effect of altering the protein expression dynamics of the bHLH transcription factor Her6 at the single-cell level in the embryonic zebrafish telencephalon. Using a homozygote endogenous *Her6:Venus* reporter and 4D single-cell tracking, we show that Her6 oscillates in neural telencephalic progenitors and that the fusion of protein destabilisation (PEST) domain alters its expression dynamics, causing most cells to downregulate Her6 prematurely. However, counterintuitively, oscillatory cells increase, with some expressing Her6 at high levels, resulting in increased heterogeneity of Her6 expression in the population. These tissue-level changes appear to be an emergent property of coupling between single-cells, as revealed by experimentally disrupting Notch signalling and by computationally modelling alterations in Her6 protein stability. Despite the profound differences in the single-cell Her6 dynamics, the size of the telencephalon is only transiently altered and differentiation markers do not exhibit significant differences early on; however, a small increase is observed at later developmental stages. Our study suggests that cell coupling provides a compensation strategy, whereby an almost normal phenotype is maintained even though single-cell gene expression dynamics are abnormal, granting phenotypic robustness.

## Teaser

**Summary:** Cell-cell coupling at population level compensates for abnormal dynamic Her6 gene expression at single-cell level, resulting in rescued early development and a mild phenotype at late stages.

## INTRODUCTION

Oscillations of transcription factor (TF) proteins are powerful transmitters of information that direct cellular outcomes in various biological contexts, for example DNA damage response, cell proliferation and development, including somitogenesis (Isomura and Kageyama, 2014). Oscillatory expression of basic helix-loop-helix (bHLH) Hairy and Enhancer of split (HES in mammals and Her in zebrafish) transcriptional inhibitors is involved in somitogenesis (Kageyama et al., 2012). HES/Her proteins have also been found to be oscillatory in the developing central nervous system (CNS) where they also drive oscillations of proneural factors such as DLL1 (Delta-like 1), NGN2 (Neurog2) and ASCL1 (aschaete-scute family bHLH transcription factor 1) (Imayoshi et al., 2013a). These oscillations, with periodicities within the range of hours (i.e. ultradian), have been shown to be important for correct progression of CNS development.

The functional relevance of TF oscillations has been examined with a wide range of experimental approaches to alter their dynamic expression. These include methods such as exogenous TF expression from a ubiquitous promoter (Marinopoulou et al., 2021; Sabherwal et al., 2021) or even direct optogenetic manipulation of dynamics (Imayoshi et al., 2013b; Isomura et al., 2017; Shimojo et al., 2016). Many of the manipulations of oscillatory dynamics aimed to uncover function have been based on the molecular requirements for generating oscillatory expression (Novák and Tyson, 2008). Specifically, oscillations are often based on a negative feedback loop coupled with biological time delays, associated with transcription and translation, as well as instability of mRNA and protein. Negative autoregulation is a common feature of the HES proteins, first reported in the mouse (Hirata et al., 2002; Bessho et al., 2003) and confirmed for Zebrafish Her proteins (Lewis, 2003; Giudicelli et al., 2007; Brend and Holley, 2009). In CNS development, changes in mRNA stability/translation have been achieved by mutating or blocking microRNA binding sites in the endogenous gene (Bonev et al., 2012; Soto et al., 2020), and changes in time delays have been achieved by changing the length of introns (Ochi et al., 2020; Shimojo et al., 2016). Collectively, these functional studies have shown that HES/Her oscillations are important in dictating cell-fate transitions. These include maintaining mouse telencephalic neural progenitors in development (Imayoshi et al., 2013a; Shimojo et al., 2008), the transition from progenitor to differentiation states in zebrafish hindbrain (Soto et al., 2020) as well as neural stem-cell exit from quiescence (Marinopoulou et al., 2021; Sueda and Kageyama, 2020).

Another intriguing layer of complexity in HES/Her dynamics is their upstream regulation by Notch signalling, enabling cell state decisions to be integrated between neighbouring cells. However, the tissue-level organisation of oscillators is far from intuitive and requires a combination of theory and real-time observation to be understood. Using such methods, it has been recently reported that, in the developing mouse CNS, HES5 oscillators are organised in microclusters of synchronised cells which, in addition, are spatially periodic and temporally dynamic (Biga et al., 2021). This complex organisation requires tuning of cell-cell coupling via Notch signalling and, in turn, it controls the spatiotemporal dynamics of neuronal differentiation (Biga et al., 2021; Hawley et al., 2022). However, whether such tissue-level organisation has additional roles during perturbed development is not currently known.

To characterise single-cell and tissue-level properties of the HES/Her oscillators in perturbed tissue, we have focused on *her6* (the *Hes1* orthologue) in the optically superior vertebrate model zebrafish within the context of the developing zebrafish telencephalon. First, we investigated the changes in Her6 single-cell oscillatory dynamics by alterations of a less explored molecular requirement for oscillations, protein stability, which has been shown to be important in somitogenesis (Hirata et al., 2004). Second, we examined the tissue-level response to the altered single-cell dynamics and in relation to the observed phenotype. To reduce protein stability, we have taken advantage of the CRISPR/Cas9 technology to insert a Venus fluorescent protein fused to a protein destabilisation (PEST) domain at the C-terminus of the endogenous Her6. PEST sequences are signals for making proteins susceptible to rapid proteolysis and they can exert this effect when fused to other proteins (Aulehla et al., 2008; Li et al., 1998; Ninov et al., 2012; Rogers et al., 1986).

Here, we report that Her6 oscillates in zebrafish telencephalic progenitors and that, surprisingly, destabilising Her6 results in an increase in the proportion of progenitors that show oscillatory Her6 expression. As expected, destabilising Her6 results in a pronounced and premature downregulation of Her6 during development and, overall, a lower level of Her6 protein in the majority of these telencephalic progenitors. However, counterintuitively, some cells retain high levels of Her6 expression. Together, the opposing effects in Her6 levels leads to interspersion of high-low cells and increased expression heterogeneity in the population.

Experimentally disrupting Notch signalling and computational modelling suggests that this increased variability of Her6 expression in neighbouring cells is a tissue-level property that emerges out of the coupling of Her6 dynamics between cells. Surprisingly, despite the altered Her6 expression at a single-cell level, the telencephalon was mostly phenotypically normal early on (24 hpf) in terms of size and differentiation, with some increased neurogenesis observed at late stages (48 hpf). We conclude that when Her6 is destabilised, a tissue-level communication serves to partially ‘rescue’ Her6 levels in some cells and to increase the proportion of cells with oscillatory Her6 expression, thus providing some phenotypic robustness to the population in the face of altered molecular dynamics in single cells.

## RESULTS

### *Her6:Venus* is a faithful reporter of proliferative embryonic neural progenitors in zebrafish telencephalon

**Fig. 1.**
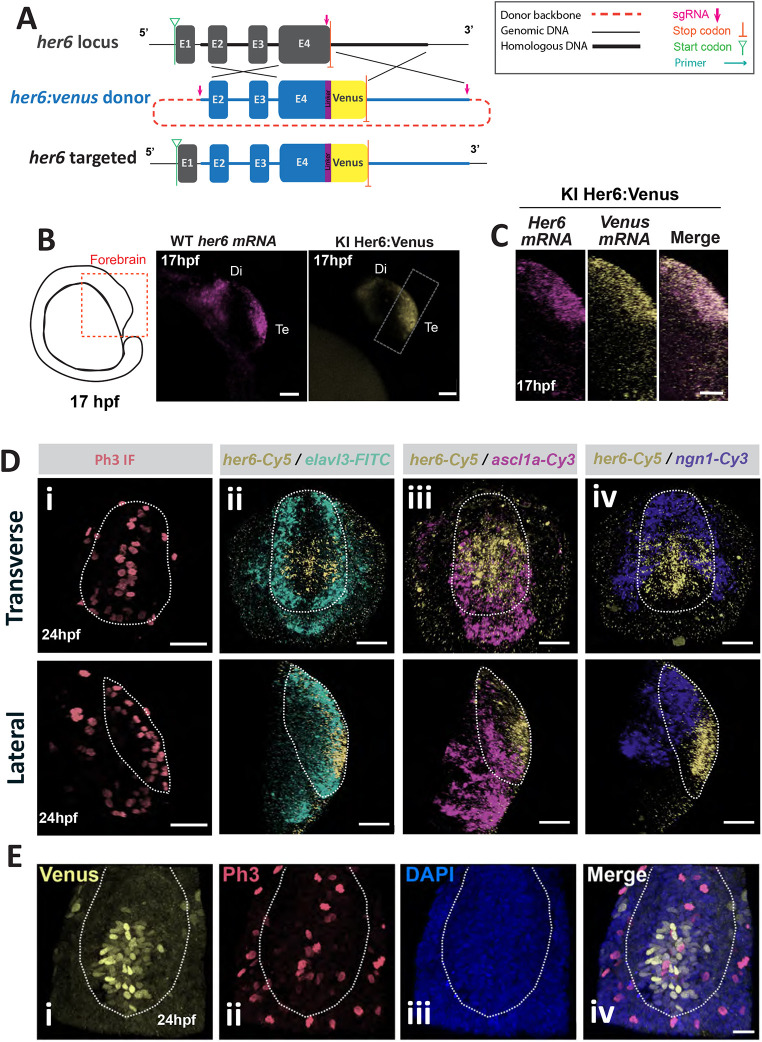
***her6* is expressed in a subpopulation of neural progenitor cells in *Her6:Venus* zebrafish telencephalon.** (A) Schematic of the *Her6:Venus* knock-in design from Soto et al. (2020). (B) Left: schematic depicts the lateral view of zebrafish embryo forebrain at 17 hpf. Middle: maximum intensity projection (MaxIntProj) image of WM FISH of endogenous *her6* in wild type (WT) embryo. Right: snapshot of live Her6:Venus (HV) expression in single *z*-plane from homozygous (HOM) embryo. Di, diencephalon; Te, telencephalon. (C) MaxIntProj images in lateral view from double WM FISH against *her6* and *venus* mRNAs in 17 hpf HOM *HV* embryo. (D) Images of the telencephalon from 24 hpf HOM *HV* embryos in transverse (top) and lateral (bottom) view: (i) WM immunofluorescent staining for Phospho-histone H3 (Ph3); (ii) WM FISH against *her6/elalv3*; (iii) WM FISH against *her6/ascl1*; (iv) WM FISH against *her6/ngn1*. (E) Transverse section of the telencephalon in HOM HV embryo at 24 hpf showing immunofluorescence labelling against Her6-Venus (i) and Ph3 (ii) accompanied by DAPI (iii); merged image indicates co-localisation (iv). Dotted contour indicates presumptive Te boundaries. Scale bars: 50 µm (B); 20 µm (C,E); 40 µm (D).

Her6 has been well-characterised in the development of the hindbrain and hypothalamus (Pasini et al., 2001; Scholpp et al., 2009). To characterise Her6 expression dynamics in zebrafish telencephalon, we used the *Her6:Venus* transgenic line (*HV*) that generates the endogenous protein fusion Her6:Venus (HV), as described by Soto et al. (2020) (Fig. 1A). Here, we focused on characterising Her6 in the forebrain using embryos of 17-24 h post fertilisation (hpf) to cover early neurogenesis (Fig. 1B-E; Fig. S1). First, we compared HV protein from homozygote *HV* knock-in embryos with *her6* mRNA from wild-type embryos in the forebrain region and observed a broadly similar HV pattern of expression (Fig. 1B). Double whole-mount fluorescent *in situ* hybridisation (WM FISH) against endogenous *her6* and *venus* in homozygous *HV* knock-in embryos (Fig. 1C) also showed broad co-localisation. These findings showed that Her6 is expressed in the rostral and ventral telencephalon and that there is no aberrant expression, confirming the suitability of the *HV* model for studying Her6 telencephalic expression, see also Soto et al. (2020). Second, we investigated the spatial localisation of *her6* in relation to known markers of proliferation and differentiation (Fig. 1D). *her6* expression was found in proliferating progenitors at the telencephalic midline, marked by the anti Phospho-Histone H3 (Ph3) staining (Fig. 1E), but the domain extended further laterally and showed partial overlap with the domain of the early post-mitotic neural marker *ELAV like neuron-specific RNA binding protein 3* (*elavl3*) (Kim et al., 1996) as we have shown before in the hindbrain (Soto et al., 2020) and less so with the proneural/neurogenic factors *ascl1a* and *ngn1* (*neurogenin 1; neurog1*) (Schmidt et al., 2013). These results suggest that *her6* is expressed in proliferative embryonic neural progenitors, continues to be expressed as cells make a transition to differentiation and is switched off in differentiated cells (Fig. 1D).

### Her6 telencephalic expression exhibits heterogeneity, due to ultradian oscillations and asynchronous downregulation in single cells

**Fig. 2.**
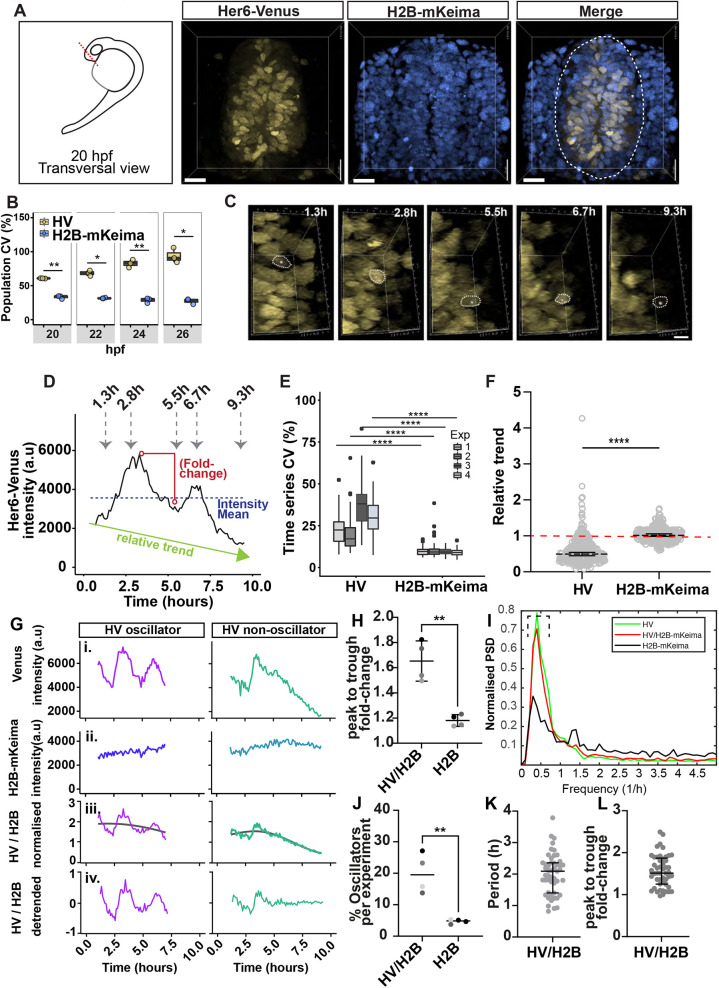
**Her6 expression is heterogeneous across the telencephalon in *HV* embryos.** (A) Schematic of 20 hpf embryo, dashed red line indicates the position of live imaging (left). Transverse view of 3D-reconstructed confocal live images (right) from 20 hpf telencephalon of *HV* embryo showing HV (yellow) and H2B-mKeima (blue). Dashed contour indicates the telencephalon boundaries. (B) Coefficient of variation (CV) at population level for HV and H2B-mKeima in *HV* embryos at 20-26 hpf. Boxplots indicate median with interquartile range; dots represent average CV per time point per embryo. (C) 3D-reconstructed snapshot confocal images depicting a single cell expressing HV over time, starting at 20 hpf. (D) HV expression over time of the single cell marked in C. The arrows indicate the time points of snapshots shown in C. The graph also depicts dynamic parameters including intensity mean of a single cell over time (blue dashed line), relative trend (green arrow) and fold-change (red circles). On *x*-axis 0=20 hpf. (E) Boxplots comparing the CV for each cell trace over time between HV and H2B-mKeima time-series from the same cell. Boxplots indicate median with interquartile range (IQR), whiskers indicate maximum (maximum value in the data, Q3 +1.5× IQR) and minimum (minimum value in the data, Q1 + 1.5× IQR) points, dots represent outliers. (F) Comparison of relative trend between HV and H2B-mKeima obtained from the same cell trace. Values >1 indicate upregulation, values=1 indicate steady expression and values <1 indicate downregulation. Bars indicate median with interquartile range, circles represent cells. (G) Representative examples of oscillatory and non-oscillatory HV time-series (i) with corresponding H2B-mKeima traces (ii) obtained from the same nuclei. Corresponding HV traces after being divided by H2B-mKeima (iii, HV/H2B normalised). Normalised traces after removing the long-term trend (iv, HV/H2B detrended). (H) Comparison of peak-to-trough fold-change in HV/H2B (Her6:Venus/H2B-mKeima) versus H2B (H2B-mKeima) collected in the same nuclei. Bars indicate median and interquartile range; each dot is median per embryo. (I) Aggregate power spectral density (PSD) obtained from time-series HV, HV/H2B and nuclear control H2B-mKeima from one experiment. (J) Percentage of oscillators in detrended HV/H2B versus H2B control. Bars indicate median, each dot indicates median per embryo. (K,L) HV period and peak-to-trough fold-change from HV/H2B detrended single cells. Bars indicate median and interquartile range, dots indicate cells. *P≤0.05; **P≤0.01; ****P≤0.0001 (statistical test details found in Table S6). Scale bars: 20 µm (A); 5 µm (C).

*her6* mRNA can be detected in the presumptive forebrain as early as 11 hpf and persists in the developing telencephalon up to at least 30 hpf (Fig. S1). To characterise Her6 dynamics in the telencephalon, we performed live imaging at 20-30 hpf to capture the time window with the highest rate of neural differentiation while Her6 was still expressed (Fig. 2A-C).

*HV* knock-in embryos were injected with *h2b-mkeima* mRNA (nuclear marker) for the purposes of segmentation and single-cell analysis (Materials and Methods). Snapshot images from transverse views of the telencephalon at 20 hpf showed a heterogenous HV expression among single cells (Fig. 2A). This was confirmed by estimating the coefficient of variation (CV), the ratio of standard deviation (s.d.) of nuclear intensities at a specific time to the mean, which was used to measure snapshot heterogeneity of expression in the cell population (Fig. 2B). HV expression appeared progressively more heterogeneous at 20-26 hpf: this effect was not, however, observed for the stable nuclear marker H2B-mKeima collected from the same nuclei (Fig. 2B).

This increase in heterogeneity during development could be the result of changes in single-cell HV expression over time. For example, Her6 expression may be fluctuating between low and high values, and/or it may downregulate as development progresses (long-term trend). HV expression was tracked at a single-cell level and we observed fluctuations in protein expression (see example in Fig. 2C with matching timestamps in Fig. 2D). To confirm the presence of heterogeneity in HV expression in single-cell traces, we measured the CV in single-cell time-series (CV_t_) (Fig. 2E). HV expression had significantly higher variability compared with H2B-mKeima, showing that the population heterogeneity (Fig. 2B) arises from changes in HV expression over time at the single-cell level (Fig. 2E).

H2B-mKeima time-series had relative trend ratios close to 1 showing steady expression over time, whereas the large majority of HV time-series showed downregulation (Fig. 2F). Overall, the presence of downregulation contributed to the observed Her6 single-cell heterogeneity (Fig. 2E). However, Her6 also exhibited fluctuations in expression overlaid onto a slow-varying downward trend (Fig. 2D), so we investigated whether these short-term dynamics also contributed to Her6 heterogeneity.

We have previously shown, in hindbrain, Her6 ultradian oscillatory protein expression at single-cell level (Soto et al., 2020). Thus, we investigated ultradian activity in time-series of HV in single neural progenitors from the telencephalon (Fig. 2G-L). Single cells were tracked to monitor HV (Fig. 2Gi) and H2B-mKeima (Fig. 2Gii) fluorescence intensity over time and HV was normalised to mKeima fluorescence intensity to remove any potential imaging artefacts (Fig. 2Giii, HV/H2B normalised). Then, to focus on the ultradian dynamics, we removed the long-term trends (such as downregulation) in the normalised traces (Fig. 2Giv, HV/H2B detrended). In the zebrafish telencephalon, both oscillatory and non-oscillatory HV expression was observed (Fig. 2G; Fig. S2A). We used the Hilbert transform to measure peak-to-trough fold-change as a means to describe amplitude of intensity fluctuations (Fig. S2B) (Manning et al., 2019). The average Her6 fold change in all cells ranged between 1.5-1.8× and was more prominent than noisy fluctuations observed in nuclear H2B-mKeima (Fig. 2H). Frequency analysis indicated that the Venus signal has a prominent peak around 2.5 h, indicative of the presence of oscillatory activity (Fig. 2I). The dominant frequency peak was closely reproduced by the HV/H2B signal and, as expected, appeared severely dampened in the H2B-mKeima (Fig. 2I).

To distinguish periodic (oscillatory) expression from stochastic aperiodic (non-oscillatory) expression, single cell HV/H2B traces were analysed using a statistical method of stochastic oscillatory activity detection using Gaussian processes (Materials and Methods; Phillips et al., 2017; Soto et al., 2020). HV oscillatory cells represented 15-30% of the total neural progenitor cells analysed (Fig. 2J). Due to an imposed false discovery rate (FDR), only a small number of H2B-mKeima traces were found to be oscillatory (∼5%; Fig. 2J) and these appeared to be strikingly different from the HV oscillators (Fig. 2G; Fig. S2A). Importantly, the percentage of oscillators detected in HV/H2B was significantly higher than those observed in nuclear H2B-mKeima in the same cells (Fig. 2J). The detected oscillators showed a median of 1.9 h period ranging between 1 h and 3 h (Fig. 2K) in agreement with previous reports (Soto et al., 2020) and similar to the timescale described for Her1 during somite formation (Rohde et al., 2021 preprint), although longer than that described in Delaune et al. (2012), which could be owing to changes in time delays or coupling parameters (Lewis, 2003). The fold-change ranged between 1.5× and 2.5× (Fig. 2L), like the overall fold-change in the population (Fig. 2H). This indicated that both oscillatory and non-oscillatory ultradian dynamics contribute to gene expression heterogeneity.

### Decreasing HV protein stability increases the number and amplitude of oscillators and promotes downregulation

**Fig. 3.**
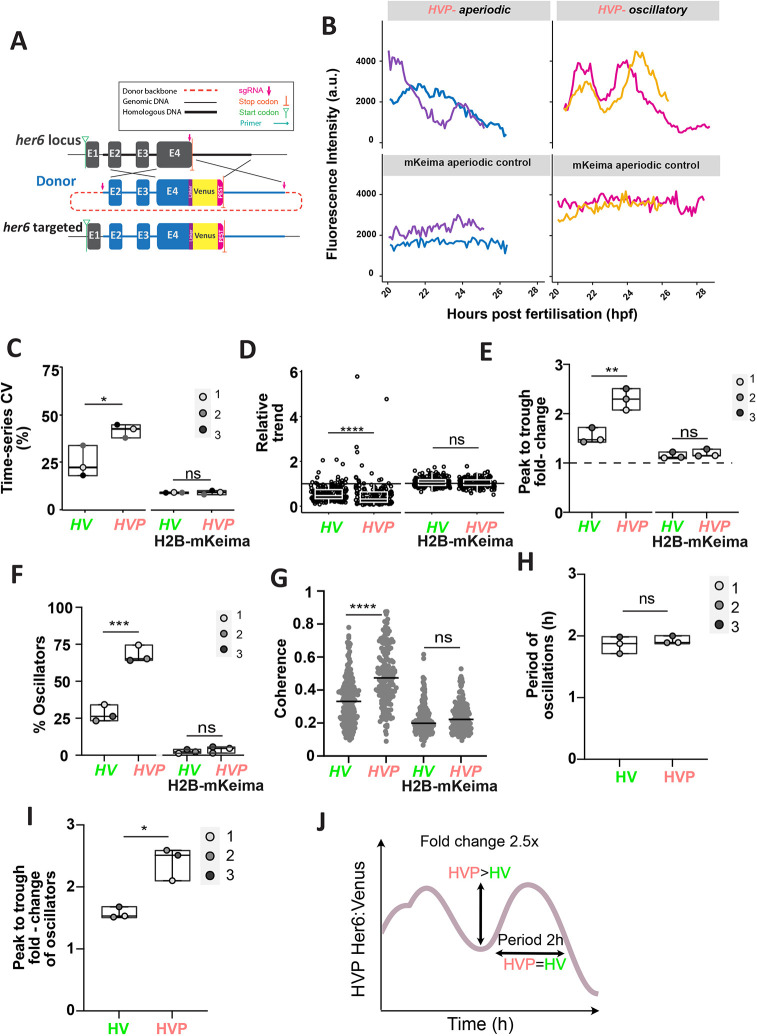
**Her6 expression dynamics in single cells are altered when the protein is destabilised.** (A) Schematic of the *Her6:Venus-PEST* (*HVP*) knock-in design. CRISPR/Cas9-mediated HDR resulted in the insertion of the KI cassette including Venus and PEST into the endogenous locus. Detailed description is presented in Fig. S3A,B. (B) Representative examples of Venus oscillatory (HVP-oscillatory) and Venus non-oscillatory (HVP-aperiodic) and their corresponding H2B-mKeima time-series obtained from *HVP* embryos at 20-30 hpf. (C) Comparison of time-series CV from raw single-cell Venus and H2B-mKeima observed in *HV* versus *HVP*. ns: *P*=0.9381. (D) Relative trend of Venus and H2B-mKeima raw time-series in *HV* versus *HVP* embryos. Values >1 indicate upregulation, values=1 indicate steady expression and values <1 indicate downregulation. Circles represent individual cells. ns: *P*=0.4207. (E) Peak-to-trough fold-change in oscillators expressing raw Venus and their respective mKeima compared in *HV* and *HVP* embryos. ns: *P*=0.4798. (F) Percentage of oscillators in *HV* versus *HVP* embryos. Cells expressing Venus (HV and HVP, respectively) and mKeima (H2B-mKeima from the respective *HV* and *HVP* embryos). ns: *P*=0.3609. (G) Coherence values observed in Venus and mKeima compared between *HV* and *HVP* time-series. Dots represent individual cells, line indicates median. ns: *P*=0.8239. (H,I) Period and peak-to-trough fold-change of oscillatory HV versus HVP expression. ns: *P*=0.3165. (C-I) Boxes indicate median with interquartile range. Dots represent median per embryo in C,E-I. (J) Diagram representation of the main features of HVP oscillations. *P≤0.05; **P≤0.01; ***P≤0.001; ****P≤0.0001 (statistical test details found in Table S6). ns, not significant.

We hypothesised that changes in Her6 protein properties, such as its rate of degradation, would affect its dynamic behaviour, with a concomitant effect on neural differentiation. To test this hypothesis, we created an in-frame fusion of the endogenous Her6 protein with Venus fluorescent protein plus a protein destabilising PEST domain at the C terminus, generating the *Her6:Venus:PEST* (*HVP*) zebrafish knock-in line (Fig. 3A; Fig. S3A,B; Materials and Methods). The spatial localisation of the *her6* domain in *HVP* was comparable with *HV* (Fig. S3C,D). We also validated this knock-in by conducting WM FISH against endogenous *her6* and *venus* in homozygous *HVP* knock-in embryos (Fig. S3E,F). To confirm that the insertion of the PEST domain destabilises the HV protein, we carried out protein half-life experiments (Fig. S4A-D). As previously shown, the Her6 protein half-life is shorter (11 min; Soto et al., 2020) relative to its mouse orthologue HES1 (22.3 min; Kobayashi et al., 2015). We expected that by adding one PEST domain, this would reduce even further the Her6 protein half-life (potentially in the range of few minutes) and would therefore make it technically more difficult to estimate in zebrafish. As an alternative, we used human mammalian cells and this allowed us to confirm that HVP has shorter half-life than HV expressing cells (Fig. S4A-D).

To test the effect of changing the Her6 protein stability, we compared HV with HVP protein dynamics in single cells at 20-28 hpf. Statistical detection revealed that, similar to HV dynamic protein expression, both oscillating and non-oscillating Her6:Venus expression were present in *HVP* (Fig. 3B). To examine Her6 fluctuations more quantitatively, we analysed the time-series CV, which revealed that HVP time-series were significantly more variable across all experiments when compared with HV (Fig. 3C; Fig. S4E). The increased heterogeneity in *HVP* was in part due to differences in the long-term trend, quantified as relative trend ratio, where HVP showed more drastic downregulation relative to HV (Fig. 3D; Fig. S4F).

We found that the peak-to-trough fold-change values in HVP time-series were consistently higher than HV across experiments (Fig. 3E). We also observed a significant increase in the percentage of oscillators in HVP when compared with HV, (Fig. 3F; ∼70% and ∼25% in HVP versus HV, respectively) validated using an independent frequency-based measure by which we observed that coherence of oscillations increased in HVP compared with HV (Fig. 3G). The oscillatory period remained unaltered (Fig. 3H; Fig. S4G), whereas the fold-change increased from ∼1.6× in HV to ∼2.5× in HVP (Fig. 3I; Fig. S4H). As expected, H2B-mKeima showed no differences between HV and HVP conditions (Fig. 3C-G; Fig. S4E,F) and an acceptable FDR of 5% (Fig. 3F).

Overall, these quantitative comparisons showed that the increased single-cell variability in HVP compared with HV is a composite of increased Her6 downregulation, an unexpected rise in the proportion of cells with oscillatory expression and an increase in peak-to-trough fold-change values (Fig. 3J).

### Decreasing Her6 stability increases spatial heterogeneity in single-cell protein expression in the telencephalon

**Fig. 4.**
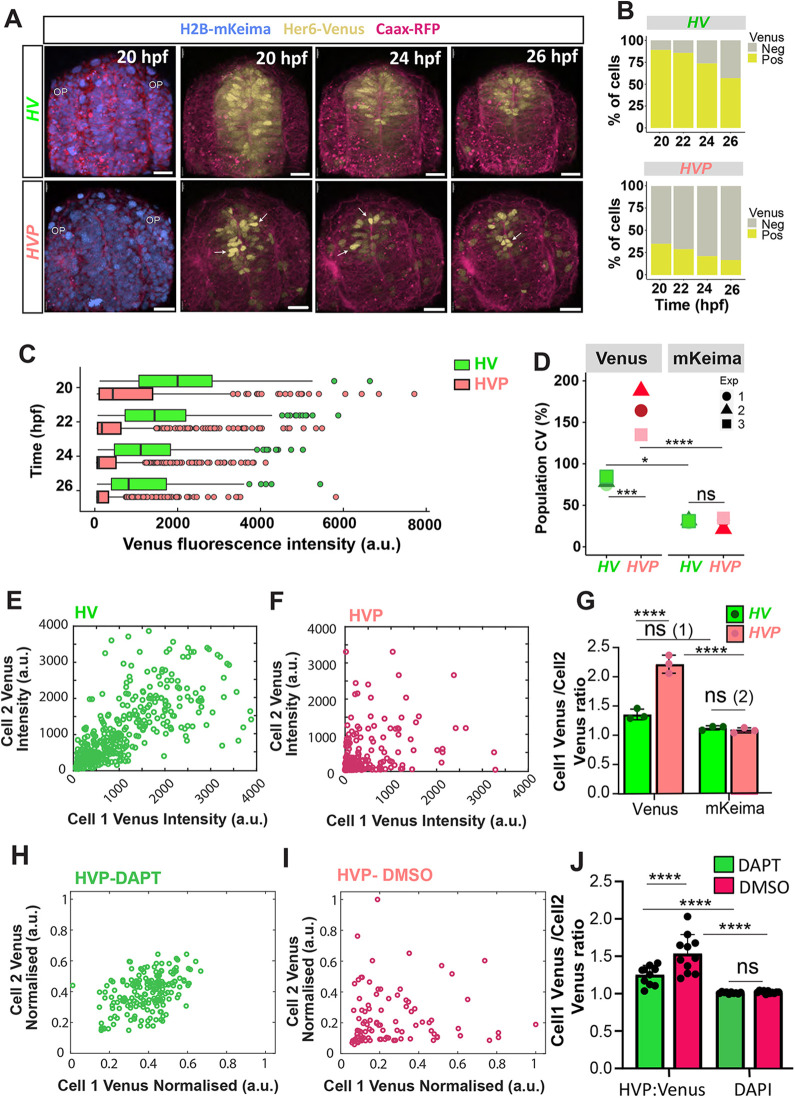
**Spatial heterogeneity in Her6:Venus expression is altered in embryos with destabilised protein.** (A) Representative examples of 3D-reconstructed confocal images of the transverse view of the telencephalon in *Her6:Venus* (*HV*; top panels) and *Her6:Venus-PEST* (*HVP*; bottom panels) embryos at 20-26 hpf showing HV and HVP expression (yellow), nuclei (H2B-mKeima, blue) and membranes (CAAX-RFP, red). Arrows indicate highly expressing HVP cells. OP, olfactory placode. (B) Percentage of Venus-expressing cells in *HV* versus *HVP* from a single experiment. (C) Distribution of nuclear HV and HVP at 20-26 hpf (temporal breakdown by repeat in Fig. S5C). Markers represent individual nuclei per time point, boxes indicate median and interquartile range. (D) Comparison of population CV obtained from Venus (HV and HVP) and H2B-mKeima expression from *HV* and *HVP* embryos (temporal breakdown by repeat in Fig. S5D). (E,F) Local intensity mapping of Venus in neighbouring cells observed in *HV* (E) and *HVP* (F) embryonic telencephalon imaged simultaneously at 24 hpf. Dots indicate intensities in individual nuclei (Cell 1) that were paired with their nearest neighbour (Cell 2) based on 3D Euclidean distance. (G) Intensity ratios observed in neighbouring cells (see E,F) showing Venus and H2B-mKeima quantified in *HV* versus *HVP* embryos at 20-26 hpf. Bars indicate mean±s.d., dots represent mean per embryo. ns (1): *P*=0.0901; ns (2): *P*=0.9989. (H,I) Local intensity mapping of Venus in neighbouring cells observed in DAPT- (H) and DMSO- (I) treated *HVP* embryos at 24 hpf. Dots indicate intensities in individual nuclei (Cell 1) that were paired with their nearest neighbour (Cell 2) based on 3D Euclidean distance. (J) Average intensity ratios observed in neighbouring cells (see H,I) showing Venus and H2B-mKeima quantified in *HVP* embryos at 24 hpf treated with DAPT versus DMSO. Bars indicate mean±s.d., dots represent mean per embryo. ns: *P*>0.9999. *P≤0.05; ***P≤0.001; ****P≤0.0001 (statistical test details found in Table S6). ns, not significant. Scale bars: 30 µm.

Following the characterisation of Her6 protein dynamics at a single-cell level, we set out to investigate protein expression differences at tissue level. Snapshots from live imaging demonstrate that fewer cells expressed Her6 in *HVP* and their expression appeared to be more variable between neighbouring cells than in *HV* at 20, 24 and 26 hpf (Fig. 4A; Movie 1). To quantify this difference, we used the H2B-mKeima to detect nuclei in the imaged domain of the telencephalon (in 3D snapshots in different stages), in both *HV* and *HVP*, regardless of their Venus expression. To separate Venus(+) and Venus(−) cells, a detection threshold was set for each embryo based on background measurements (see Materials and Methods). The analysis reflected a lower abundance of cells expressing Venus for the duration of live imaging in *HVP*, quantified as the proportion of Venus(+) cells over the total number of cells expressing H2B-mKeima (Fig. 4B; Fig. S5A). We also observed a tendency for faster decrease of Venus(+) cells in *HVP* when compared with *HV* (Fig. S5B). Consistently, we observed a reduced volume of the *her6* domain in *HVP* compared with *HV* (Fig. S5C). However, the *her6* relative expression by quantitative reverse transcription polymerase chain reaction (RT-qPCR) was unaffected (Fig. S5D), which may be an effect of population averaging masking any changes in heterogeneity of expression. Indeed, many HVP cells had very low expression, as expected from the protein destabilisation; however, unexpectedly some HVP cells expressed Her6 at high HV levels (Fig. 4C; Fig. S5E). Further analysis at population level confirmed that *HVP* has higher variability when compared with *HV* (Fig. 4D; Fig. S5F,G). As expected, we observed minimal population heterogeneity in nuclear markers H2B-mKeima and DAPI (Fig. 4D; Fig. S5F,G).

Overall, these results suggest that a decrease in Her6 stability increases both the temporal protein expression heterogeneity at a single-cell level and the spatial protein expression heterogeneity at a population level.

### Increased differences in Her6 expression in *HVP* neighbouring cells become reduced and more similar to *HV* when Notch signalling is blocked

We investigated the relationship of Her6 expression between neighbouring cells in *HV* and *HVP* embryos at 24 hpf (Fig. 4E-G; Fig. S6A). For each cell, the closest neighbour was identified, and the intensities were plotted as paired sets, with Venus intensity of a selected cell 1 on the *x*-axis and its neighbouring cell 2 intensity on the *y*-axis (Fig. 4E,F). The intensity correlation in neighbouring cell 1 and cell 2 intensities was significantly reduced in *HVP* (∼0.35) compared with *HV* (∼0.56) (Fig. S6B). This reduction was primarily from a difference in level observed persistently in *HVP* neighbouring cells over time (Fig. S6C). As expected, H2B-mKeima expression appeared to be more randomly distributed and similar between *HV* and *HVP* (Fig. S6A,B). We quantified the difference in intensity between neighbouring cells using a cell 1 Venus/cell 2 Venus ratio (Fig. 4E,F) and reported average ratio values per embryo (Fig. 4G; Fig. S6D; temporal breakdown). Intensity ratios in *HV* neighbours were low, indicating similarity (∼1.4), and increased significantly in *HVP* to >2 (Fig. 4G; Fig. S6D). As expected, mKeima local intensity ratios were ∼1 for both *HV* and *HVP* (Fig. 4G). In summary, neighbouring cells in *HV* have more similarity in local intensity resulting in small differences in Her6 between neighbouring cells, whereas neighbouring *HVP* cells are more heterogenous in Her6 expression.

As vertebrate genes of the HES/Her family are targets of Notch signalling (Delaune et al., 2012; Naganathan and Oates, 2020), we hypothesised that Notch-mediated cell coupling may allow some cells to ‘compensate’ for the loss of Her6 expression in their neighbours, leading to the observed heterogeneity in levels. To test this hypothesis, we exposed HVP embryos to DAPT, a gamma-secretase inhibitor that disrupts Notch signalling, or to DMSO as a control. We observed a decrease in intensity ratios between neighbouring cells in HVP embryos when Notch signalling was reduced (Fig. 4H-J), which was also reflected in the increase of correlation coefficient (Fig. S6E).

In conclusion, the quantification of local and global differences in the Her6-Venus reporter suggests that, as protein stability is reduced, the heterogeneity in protein expression becomes increased; however, this is reversed when Notch-dependent cell coupling is disrupted.

### The unexpected increase in Her6 expression heterogeneity can be mathematically explained by cell-cell coupling

**Fig. 5.**
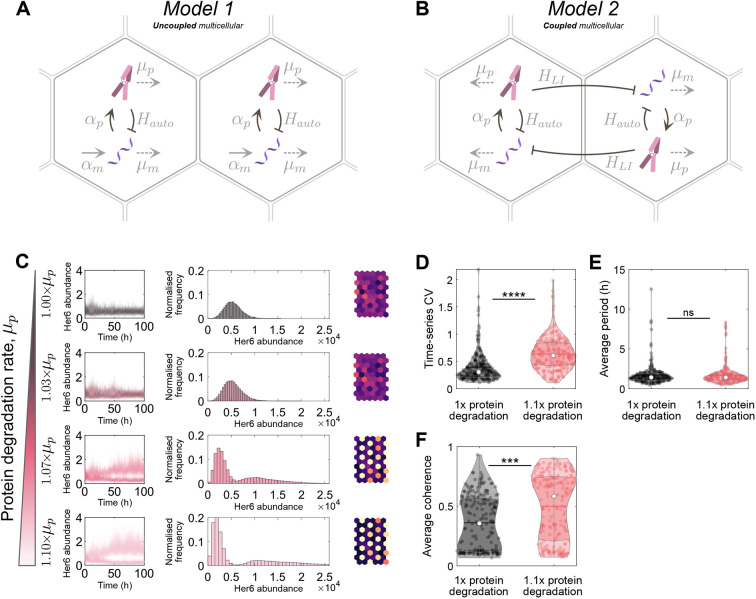
**Single and multicellular models of Her6 protein expression with normal and altered protein stability.** (A) Model 1: a stochastic single-cell model that explicitly models mRNA and protein. *α*_*p*_ and *α*_*m*_ are protein and mRNA synthesis rates, respectively, and *μ*_*p*_ and *μ*_*m*_ are the degradation rates. *H*_*auto*_ is the Hill function used for the direct repressive action of Her6 protein on its own mRNA production, which has an associated time delay (Table S1). (B) Model 2: the same as Model 1 but extended to a multicellular model. Cells are coupled bidirectionally with a repressive Hill function, *H*_*LI*_, that has an associated time delay and represents Notch-Delta lateral inhibition signalling. For all simulations, both models were run as a hexagonal grid of 10 rows by 6 columns. (C) Her6 expression from Model 2 run with a parameter set contained within the accepted optimiser parameter sets. Each row corresponds to a different protein degradation rate (indicated on the left, ranging between 1× and 1.1×). The first column shows the time-series of every cell for the full simulation. The second column shows histograms of Her6 abundance taken from the second half of the simulations. The third column shows the final time point of each simulation plotted on a hexagonal grid in which colour indicates Her6 expression in each cell (lighter colours indicate higher expression). (D) Time series coefficient of variation (CV_t_) plotted at normal and 1.1× degradation. For normal degradation rate, median CV_t_=0.30 (white dot), and for 1.1× degradation, median CV_t_=0.61. Kruskal–Wallis statistical test showed a significant difference in the distributions, *P*=1.3×10^−20^. (E) Median temporal period for each parameter set. Median period was 141 h in both cases. Kruskal–Wallis statistical test showed no significant difference in the distributions, *P*=0.55. (F) Median coherence for each parameter set. Median coherence for normal degradation was 0.36, and for 1.1× degradation coherence was 0.58. Kruskal–Wallis statistical test showed a significant difference in the distributions, *P*=1.5×10^−8^. ***P≤0.001; ****P≤0.0001. ns, not significant.

To understand whether increased protein degradation alone can account for the HVP increase in heterogeneity we used mathematical modelling, analysing two stochastic delay differential equation models of Her6 expression. Model 1 is an uncoupled multicellular model in which individual cells are explicitly modelled as Her6 protein repressing its own mRNA expression (Fig. 5A). This autoinhibition is implemented using a repressive Hill function with an associated time delay as in Monk (2003). Model 2 extends Model 1 to include Notch-Delta signalling between cells (Fig. 5B). This cell-cell coupling is achieved by introducing a bidirectional repressive Hill function between cells – again with an associated time delay – so that Her6 protein can influence mRNA expression in neighbouring cells (see Materials and Methods). By comparing these two models, the importance of single-cell dynamics and lateral inhibition in the HVP phenotype were analysed.

Model 1 could not produce any parameter sets that led to a substantial increase in heterogeneity, whereas many parameter sets were identified for Model 2 (distributions of accepted parameter values shown in Fig. S7A)*.* This indicates that increasing protein degradation can indeed lead to increased heterogeneity, but only in the case when cells are coupled via lateral inhibition.

To understand how Her6 expression is changing in Model 2 when protein degradation rate is varied, distributions of Her6 expression were plotted in Fig. 5C, starting from a degradation rate of 1× (top; representing HV) to degradation rate of 1.1× (bottom; representing HVP). The parameter set used was randomly selected from the group of accepted optimiser parameter sets, and other example outputs using different parameter sets are shown in Fig. S7B,C. This highlights that CV increases due to cells being increasingly influenced by lateral inhibition as the protein degradation rate increases, resulting in a ‘salt and pepper’ pattern, and producing similar distribution shifts as in HVP.

Additionally, summary statistics of single-cell behaviour were produced for all accepted parameter sets to further compare simulation outputs with tissue measurements. Time-series coefficient of variation CV_t_ (Materials and Methods) indicated how variable the amplitude of fluctuations were in single cells across the whole time series (Fig. 5D). As is the case in the tissue, the model exhibited larger fluctuations in expression levels (higher CV_t_) with a higher degradation rate (compare with Fig. 3C). Fig. 5E shows that the ultradian periods (median 1.4 h) were largely similar to those observed in the tissue and that there was no significant change as protein degradation rate was increased (compare with Fig. 3H). Finally in Fig. 5F, the average coherence of individual cell time traces is given as an indicator of the quality of oscillations, which shows that there tended to be more oscillatory expression when the degradation rate was increased, again following the trend seen in the tissue measurements (compare with Fig. 3G).

### Embryos with altered Her6 dynamics and increased Her6 population heterogeneity are phenotypically normal at an early stage

**Fig. 6.**
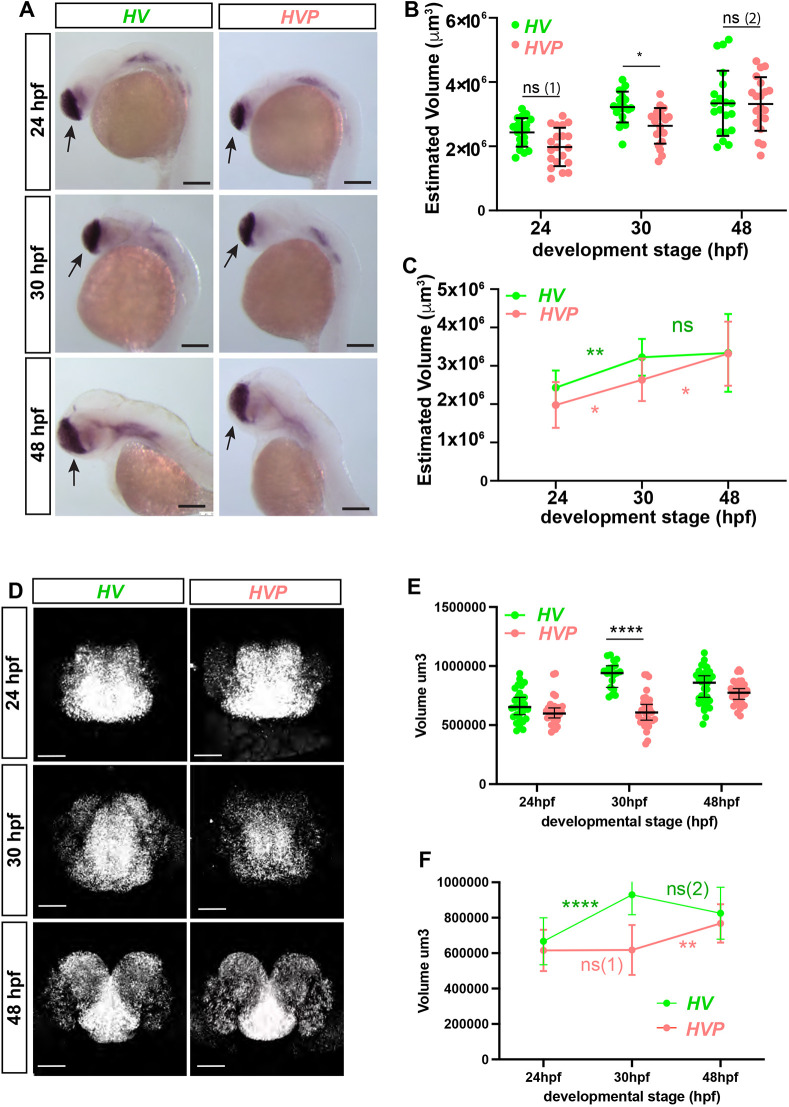
**The size of telencephalon in *HV* and *HVP* embryos at 24, 30 and 48 hpf.** (A) WM Chromogenic ISH showing *foxg1* mRNA expression in *HV* and *HVP* at specific stages. Black arrows: telencephalon. (B) 3D volumetric comparison of telencephalon size (calculated using length, depth and width measurements from data in A; see also Fig. S8A-E) in *HV* versus *HVP* from 24 to 48 hpf. Bars indicate mean±s.d., dots indicate individual embryos. ns (1): *P*=0.1104; ns (2): *P>*0.99. (C) Comparison of the changes in telencephalic volume over time. Spots and bars indicate the mean±s.d. ns: *P*>0.99. (D) Representative examples of *in situ* hybridization chain reaction (HCR) for *foxg1* mRNA observed in HV versus HVP embryos at 24, 30 and 48 hpf. (E) Volume measurements of telencephalon region marked by *foxg1* from data in D observed in *HV* and *HVP* embryos at different stages. Bars indicate median and 95% CI, dots indicate individual embryos. ns: *P*>0.99. (F) Comparison of telencephalon volume growth rates in *HV* and *HVP* embryos (data from E). Spots indicate mean, bars indicate s.d. ns (1): *P*>0.99; ns (2): *P*=0.6064. *P≤0.05; **P≤0.01; ****P≤0.0001 (statistical test details found in Table S6). ns, not significant. Scale bars: 150 µm (A); 50 µm (D).

So far, we have shown that destabilised Her6 leads to altered single-cell dynamics, accompanied by an increase in population heterogeneity due to cell coupling. Next, we sought to determine the phenotypic effects of these changes during the development of the telencephalon. First, we asked whether there was a global difference in the size of the whole telencephalon. We measured the overall volume of the telencephalon using chromogenic whole-mount *in-situ* hybridisation (WM ISH) (Fig. 6A-C; Fig. S8A-E) and hybridisation chain reaction (HCR) technique (Fig. 6D-F; Movies 2-4) against the telencephalic marker *foxg1* in 24, 30 and 48 hpf embryos. There was no difference in size at 24 hpf and despite small differences in telencephalon dimensions, particularly width, at 30 hpf (Fig. S8E), by 48 hpf the telencephalon had regained its size and appeared normal in *HVP* when compared with *HV* (Fig. 6B,C,E,F). We next examined the expression of terminal neural differentiation markers.

### Changes in differentiation are observed in later stages of telencephalic development

**Fig. 7.**
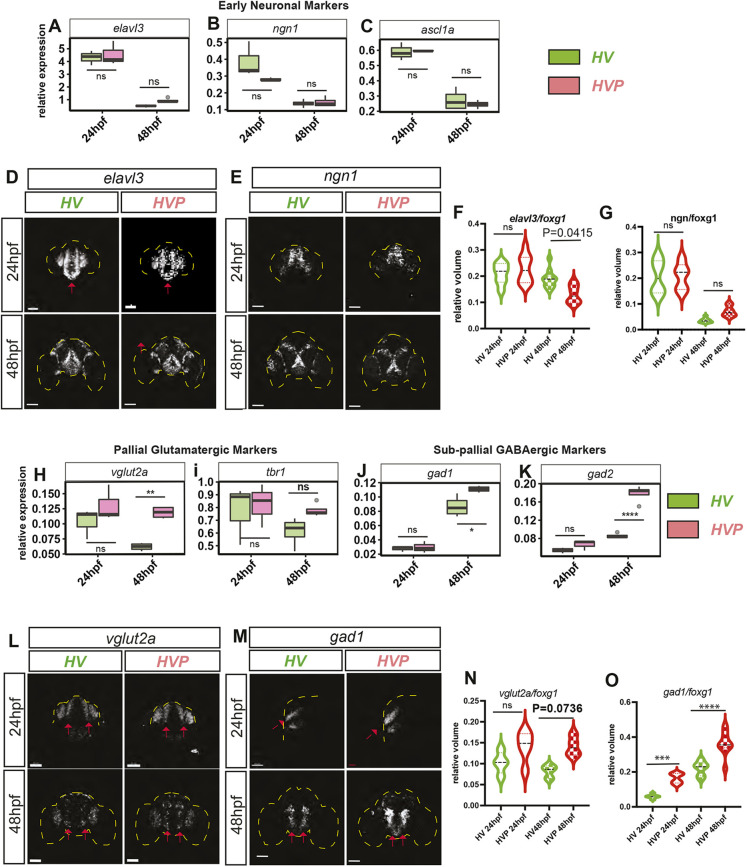
**The phenotypic effect of destabilising Her6 protein is negligible in 24 hpf embryos but becomes more evident by 48 hpf.** (A-C) qPCR quantification of mRNA expression level observed in *elavl3* (A), *ngn1* (B) and *ascl1* (C) between *HV* (green) and *HVP* (pink) in dissected telencephalon of 24 and 48 hpf embryos. Boxes indicate median and interquartile range. Whisker endpoints indicate 25^th^ percentile−1.5× interquartile range and 75^th^ percentile+1.5× interquartile range. ns: *P*>0.05. (D,E) Representative examples of *elavl3* (D) and *ngn1* (E) mRNA visualised using HCR in *HV* and *HVP* forebrain at 24 and 48 hpf. Frontal view. Red arrows indicate telencephalic region. Yellow dashed line indicates embryonic outline. (F,G) Double HCR was performed on each embryo for *elavl3* or *ngn1* with *foxg1.* Relative volume was calculated as a ratio of the respective gene with *foxg1*. (F) Relative volume *elavl3*/*foxg1* compared between *HV* and *HVP* embryos at different stages. (G) Relative volume of *ngn1*/*foxg1* compared between *HV* and *HVP* embryos at different stages. (H-K) Quantification of mRNA by qPCR showing the expression level of pallial glutamatergic markers, *vglut2a* and *tbr1* (H,I), and sub-pallial GABAergic markers, *gad1* and *gad2* (J,K). Boxes indicate median and interquartile range. Whisker endpoints indicate 25^th^ percentile−1.5× interquartile range and 75^th^ percentile+1.5× interquartile range. ns: *P*>0.05. (L,M) Representative examples of *vglut2a* (M) and *gad1* (N) mRNA visualised using HCR in *HV* and *HVP* forebrain at 24 and 48 hpf. Frontal view in L and M, 48 hpf. Lateral view in M, 24 hpf. Red arrows indicate telencephalic region. Yellow dashed line indicates embryonic area. (N,O) Double HCR was performed on each embryo for *vglut2a* or *gad1* with *foxg1.* Relative volume was calculated as a ratio of the respective gene with *foxg1*. (N) Relative volume of *vglut2a*/*foxg1* compared between *HV* and *HVP* embryos at different stages. (O) Relative volume of *gad1*/*foxg1* compared between *HV* and *HVP* embryos at different stages. *P≤0.05; **P≤0.01; ***P≤0.001; ****P≤0.0001 (statistical test details found in Table S6). Scale bars: 50 µm.

As members of the HES/Her family of proteins are transcriptional repressors of neuronal differentiation, we analysed the expression domains of *ascl1* (by RT-qPCR) and *ngn1* (by HCR and RT-qPCR) that label early differentiation in the sub-pallium and pallium, respectively – as well as the general post mitotic neurogenic progenitor marker *elavl3* (by HCR and RT-qPCR) – in *HV* and *HVP* at 24 hpf and 48 hpf (Fig. 7; Fig. S8F-I; Movies 5-12). We expected an increase, but no differences were observed in the volume of *elavl3*, *ascl1* and *ngn1* (Fig. S8F-I) or their expression level in *HV* versus *HVP* embryos at 24 hpf (Fig. 7A-C). Furthermore, no differences were observed in the expression of *ascl1* and *ngn1* at the later stage (48 hpf; Fig. 7B,C) or in the relative volume of *ngn1* (Fig. 7E,G) – although *elavl3* showed small and inconsistent changes between *HV* and *HVP* at 48 hpf (Fig. 7A,D,F). These findings suggest that there are no marked changes in differentiation as result of destabilising Her6 at an early developmental stage.

We then looked by RT-qPCR and HCR at the presence of terminally differentiated neurons, which are a clear readout of developmental output in the developing nervous system (Fig. 7H-O) at 24 and 48 hpf in the *HV* and *HVP* telencephalon. We observed that the levels of expression of the pallial glutamatergic neuronal markers *vglut2a* (*slc17a6b*) and *tbr1* (*tbr1a*), as well as subpallial GABAergic neuronal markers *gad1* (*gad1b*) and *gad2* were not different between *HV* and *HVP* at 24 hpf, but they were significantly higher in *HVP* compared with HV at 48 hpf, except for *tbr1* (Fig. 7H-K). Furthermore, the relative volume for *gad1* showed some increase also at an earlier stage (Fig. 7M,O; Movies 11,12) and the relative volume for *vglut2a* showed a tendency to increase in *HVP* at 24 and 48 hpf compared with *HV*, although there was no significant different (Fig. 7L,N; Movies 9,10).

Taken together, our observations suggest that when Her6 is destabilised in single cells there is a decline in its level of expression in many cells, as would be expected. However, a compensatory mechanism mediated by cell-cell coupling rescues Her6 protein level in some cells resulting in an increased level of Her6 heterogeneity at the population level. In turn, this results in almost normal neural differentiation and telencephalic size, in spite of the premature loss of Her6 in many progenitor cells. However, such tissue-level compensation is no longer effective at later stages of development, hence an increase in some differentiation markers is observed.

## DISCUSSION

In this manuscript, we have examined the role of protein biochemical properties in shaping Her6 dynamics at a single-cell level, and the consequences of altering such properties at the tissue level, focusing on the development of the zebrafish telencephalon. We have discovered that reducing Her6 protein stability using an in-frame fusion of a destabilising PEST domain to the endogenous protein reduces the Her6 protein levels overall and causes the Her6 expressing domain in the telencephalon to shrink prematurely over the course of development. However, the phenotypic effect was mild and only evident at late stages in development, suggesting that a compensatory mechanism may be operating to ‘rescue’ normal development.

HES/Her proteins tend to be unstable and protein instability is a prerequisite for oscillations to occur (Hirata et al., 2004); indeed, Her6 is naturally an unstable protein. The addition of a PEST domain had a small effect in the overall stability of the protein, which was revealed when the protein was introduced in a heterologous system (a mammalian cell line); yet, the effect on the protein dynamics was pronounced. The implication of our work is that small differences in protein stability at the single cell level (in the order of 10%) can have a strong effect on protein dynamics at the population level when the cells are coupled. This demonstrates that the dynamic output of the HES/Her negative feedback loop is carefully balanced around the stability values of the protein; although at this point, we cannot totally exclude any additional effects on the dynamic properties of the protein.

Alongside the expected reduction of protein abundance in most neural progenitor cells due to reduced protein stability, we have made two counterintuitive findings: first, we observed an increase in the proportion of cells with oscillatory Her6 and an increased amplitude, indicating more and better-quality oscillations; second, some progenitor cells appeared to maintain Her6 expression at the wild-type level in *HVP* embryos. As the work was carried out in homozygous stable lines of fish, we can exclude the possibility of genetic mosaicism as the cause for these findings. These unexpected results consequently meant that there was an increased heterogeneity of Her6 protein levels at the population level and increased differences between neighbouring cells. Disrupting Notch signalling with a chemical inhibitor reversed some of the heterogeneity between neighbouring cells, suggesting that it relies on cell-cell coupling.

Her6, like other HES/Her genes, is expressed in proliferating progenitor cells and, in the mouse, combinations of HES gene knockout leads to failure of progenitor maintenance and premature differentiation (Ochi et al., 2020). Thus, we expected that the premature depletion of Her6-expressing cells in *HVP* embryos would result in premature differentiation. Surprisingly, the telencephalon appeared to be normal, at least with the criteria employed here: early differentiation markers (*elavl3*, *ngn1* and *ascl1*) and overall size of the telencephalon (measured based on *foxg1* expression) at an early stage of development (24 hpf). At later stages (48 hpf), there was an increase in some terminal differentiation markers. The late manifestation of a (mild) phenotype contrasts with the early onset of Her6 expression, which has been detected as early as 11 hpf (see Fig. S1).

We surmise that cells that maintain normal Her6 levels somehow compensate for the premature loss of Her6 in other cells, perhaps by undergoing extra proliferation at the expense of differentiation. This is supported by the growth curve of the *HVP* telencephalon, which appears to ‘catch up’ with the *HV* telencephalon between 30 hpf and 48 hpf. It is tempting to speculate that the increased proportion of high amplitude oscillations is also part of a compensatory mechanism. The increase in oscillatory activity is consistent with our previous prediction that an increased protein degradation rate would allow cells to enter an oscillatory regime (Manning et al., 2019).

How do high Her6-expressing cells arise if the protein is globally destabilised? Even though our experimental results suggested that cell coupling is involved, the answer to this question is not intuitive; therefore we employed mathematical modelling to get predictive insights into the potential mechanisms. Our modelling showed that when cells are coupled, destabilisation of Her6 affects the regulatory network in such way that a bifurcation occurs between neighbouring cells, leading to a population-level increase in low/off cells, while some cells retain normal levels of Her6 expression. This effect is not observed when Her6 is modelled in uncoupled cells; in other words, the bifurcation of the population into high and low Her6-expressing cells in *HVP* embryos arises as an emergent property of cell-cell coupling, a condition satisfied by the experimental evidence for involvement of Notch signalling. Furthermore, changes in single-cell behaviour between normal and increased degradation simulations showed good agreement with the changes in period, amplitude and quality of oscillation seen in the HV and HVP phenotypes. The precise type of Notch receptor/ligand involved is variable among Hairy/E(spl) family members (Hans et al., 2004; Schmidt et al., 2013; Tseng et al., 2021) and needs further investigation. Additionally, other signalling pathways that are present in the forebrain, such as Shh (Wilson and Rubenstein, 2000; Danesin et al., 2009), may contribute to the regulation of Her6, as is the case for Hes1 (Wall et al., 2009). Presently, we cannot exclude the possibility that ectopic expression of one of the other Her family genes, as has been described in the mouse (Ochi et al., 2020), contributes to phenotypic compensation. In addition, the activity of HES/Her proteins can be controlled by homo- and heterodimerization, as has been shown for Her1/Her7 and Hes6 (which is distinct from Her6) in the context of somitogenesis (Schröter et al., 2012). Thus, altered homo-or heterodimer formation of the mutant Her6 protein may contribute to the altered expression dynamics and/or the phenotypic compensation we observe. Compensation by other genes or by changes in dimerisation properties should be taken into consideration in future studies.

In conclusion, altering Her6 protein stability has a knock-on effect on altering single-cell dynamics, but cell-cell coupling may act to rescue developmental abnormalities. Such a rescue mechanism is imperfect, because at a later stage increased differentiation is observed, suggesting that the limits of compensation may have been reached. We suggest that emergent properties of coupling single-cell HES/Her oscillations not only control the spatiotemporal order of neurogenesis, as we have shown previously (Biga et al., 2021; Hawley et al., 2022), but also provide a mechanism for phenotypic robustness to the whole tissue when single-cell dynamics are altered.

## MATERIALS AND METHODS

### Research animals

All animal work was carried out in line with the conditions of the Animal (Scientific Procedures) Act 1986 and under the UK Home Office project licence (PFDA14F2D). Animal handling was carried out by personal licence holders.

### Characterisation of the knock-ins by step-wise polymerase chain reaction and sequencing

Genomic DNA was extracted from 2-4 days post fertilisation (dpf) embryos using NP40/Proteinase K (PK) extraction method. Then 20 µl of extraction solution [25 µl of PK (New England Biolabs; NEB)/ml of NP40 lysis buffer] was added to each embryo and incubated at 55°C for 3-4 h, followed by enzyme deactivation at 95°C for 15 min.

All PCR reactions for characterisation were carried out using the Phusion High-Fidelity DNA Polymerase kit (Thermo Fisher Scientific) based on the manufacturer's instructions using the primers in Table S2. For sequencing, in some cases amplicons were cloned using Zero Blunt TOPO PCR cloning Kit (Invitrogen) using the manufacturer's protocol. Positive clones were sequenced using M13 primers included in the kit. In other cases, amplicons were directly sequenced using the same primers used for amplification.

### Molecular cloning

The ZFIN ID number for Her6 is ZDB-GENE-980526-144. To generate PCS2-Her6-Venus-HA and PCS2-Her6-Venus-PEST-HA plasmids, the Venus sequence was amplified from the Her6-Venus (HV) CRISPR donor designed by Soto et al. (2020) using the primers in Table S3 with inclusion of Eco-RI restriction sites.

The purified Venus-EcoRI amplicon was cloned into pCR-Blunt II-TOPO (Invitrogen) using the manufacturer's guidelines. The plasmid was purified from the selected colony using the QIAprep Spin Miniprep Kit (Qiagen), checked for insertion using restriction enzyme digest with Eco-RI (NEB) and sequenced (Eurofins, LIGHTRUN).

To generate both pCS2-her6-venus-HA and pCS2-her6-PEST-venus-HA vector, we excised out PA GFP from PCS2-Her6-PA GFPHA and PCS2-Her6-PA GFP-PEST-HA with Eco-RI digestion. The digested plasmid was resolved using agarose gel electrophoresis and purified from the gel using the QIAquick Gel Extraction Kit. Vectors were treated with Antarctic Phosphatase (NEB) to prevent re-ligation. PCS2-Her6-HA and PCS2-Her6-PEST-HA (vectors) and Venus-EcoRI (insert) were assembled using T4 DNA ligase (NEB) using the manufacturer's protocol. The resulting colonies were examined by colony PCR using MyTaq Red mix (Meridian Bioscience, BIO-25043) with the Venus primers shown in Table S4. Positive clones for pCS2-her6-venus-HA and pCS2-her6-PEST-venus-HA were sequenced and purified using the PureLin HiPure kit (Thermo Fisher Scientific).

### Chromogenic and WM FISH

*foxg1* antisense probes labelled with digoxigenin (Dig) were generated using the foxg1-pBluescriptII construct kindly gifted by Corinne Houart (King's College London, UK), and chromogenic WM ISH against *foxg1* was carried out as previously described (Thisse and Thisse, 2008). The antibody used to detect the riboprobes was AP-anti-DIG (Roche, 11093274910, 1:500). For WM FISH, the *her6*-Dig, *her6*-Dinitrophenol (DNP), *Elavl3*-Fluorescein (FITC), *venus*-FITC were generated from previously described constructs (Soto et al., 2020), and *ngn1*-Dig and *ascl1-*Dig probes were generated from constructs kindly gifted by Dr Laure Bally-Cuif (Institut Pasteur, France). Tyramide amplification was used after the addition of probes and horseradish peroxidase-conjugated antibodies as described by Lea et al. (2012), anti-DIG-POD (Roche, 11207733910, 1:500) and anti-FITC-POD (Roche, 11426346910, 1:500).

WM ISH embryos were submerged in glycerol on glass dishes, positioned to the desired orientation and imaged using a Leica M165 FC Stereo microscope. All image processing and measurements from ISH were carried out using the FIJI measurement tool.

WM FISH embryos were imaged using the Upright LSM 880 Airyscan microscope (Zeiss) in Fast Airyscan mode with a W Plan-Apochromat 20×/1.0 DIC (UV) VISIR M27 75 mm lens. The first step of processing was carried out using default Airyscan Processing setup in ZEN Black. Further analysis of domain sizes was carried out by generating a mask of the signal using the Surfaces tool of Imaris 9.3.1 software. The surface grain size was 2-3 µm and the threshold was set manually for each gene in each embryo based on the best representation of the signal. The volume of the mask used for the analysis was automatically quantified by the Imaris software.

### Whole mount immunofluorescence

Whole mount immunofluorescence was adapted from Mendieta-Serrano et al. (2013). Embryos were collected and fixed with 4% Formaldehyde-PBS. They were washed 2×10 min in blocking solution [PBS, bovine serum albumin (BSA) 0.1% (Sigma Aldrich, A7906) and Triton X-100 (TX-100) 1%] and 2×10 min in PBS with Triton X-100 (PBS-TX) while rocking at room temperature (RT). For permeabilisation, embryos were treated with Proteinase K (10 μg/ml in PBS) for 10 min for 24 hpf embryos or 13.5 min for 48 hpf embryos and then washed 2×5 min in PBS-TX.

Embryos were blocked for 2 h at RT with rocking. After blocking, embryos were incubated with chicken polyclonal anti-GFP antibody (Abcam, ab13970, 1:300) and rabbit polyclonal anti-Phospho-Histone H3 (Ph3) antibody (Sigma Aldrich, 06-570, 1:500) in blocking solution overnight at 4°C.

Primary antibodies were washed off the following day 1×20 min in blocking solution and further 5×10 min washes at RT with rocking. Following from this, they were incubated with Alexa Fluor 568 goat anti-rabbit antibody (Thermo Fisher Scientific, A11011, 1:500) and Alexa Fluor 488 donkey anti-chicken antibody (Jackson ImmunoResearch, 703-545-155, 1:500) in blocking solution for 3 h at RT while rocking. They were washed for 15 min and then 10 min in blocking solution and a further 10 min in PBS-TX. For nuclear staining, embryos were incubated in DAPI (Thermo Fisher Scientific, 62248) at a final concentration of 5 μg/ml at 4°C overnight while rocking. On the following day, DAPI solution was removed and embryos were washed 3×10 min with PBS-TX.

### Quantification of volume from WM ISH datasets

For each condition, 20 embryos were imaged in both transverse and lateral orientations. Using the FIJI measure tool, the length (L) and depth (D) of the telencephalon was estimated from the lateral images and the width (W) from the transverse images (Fig. S8D). The values were converted from arbitrary units to mm using the scale bar recorder on the images. The volume of the *foxg1* expression domain (calculated by multiplying the three dimensions D×L×W) was used as a proxy of the telencephalic volume.

### Hybridization chain reaction

To avoid any potential technical bias introduced by the colorimetric reaction, we have confirmed the telencephalic volume result using HCR, which has a high to noise ratio. HCR was performed following the manufacturer's instructions (Molecular Instruments). *foxg1*, *elavl3*, *ngn1*, *gad1* and *vglut2a* probes were generated by Molecular Instruments as 20 probe set sizes. We used an overnight amplification step to estimate the telencephalon volume based on presence/absence of signal. Where appropriate, we performed double HCR to co-label for *foxg1* and the desired gene in order to have a relative volumetric measurement to the telencephalic region (*foxg1*).

Following the HCR protocol, embryos were treated with 1 mg/ml DAPI in 1× PBS with 0.1% Tween-20 (PBS-Tween) for 2 h at RT. Embryos were mounted in 1% agarose and imaged using a confocal Zeiss Upright LSM880 AiryScan microscope with W Plan-Apochromat 20×/1.0 DIC (UV) VISIR M27 75 mm lens. Images were analysed using Imaris software with surface tool. Surface parameters used were:

*foxg1* 24 hpf surface grain: 5-7, *HV* threshold: 29±6%, *HVP* threshold: 26±6%;

*foxg1* 30 hpf surface grain: 7, *HV* threshold: 21±8%, *HVP* threshold: 27±6%;

*foxg1* 48 hpf surface grain: 5, *HV* threshold: 22±8%, *HVP* threshold: 21±7%;

*elavl3* 24 hpf surface grain: 2, *HV* threshold: 34.8±6%, *HVP* threshold: 34.4±6%;

*elavl3* 48 hpf surface grain: 3, *HV* threshold: 37±6%, *HVP* threshold: 35±6%;

*ngn1* 24 hpf surface grain: 5, *HV* threshold: 41±7%, *HVP* threshold: 34±6%;

*ngn1* 48 hpf surface grain: 5, *HV* threshold: 45±10%, *HVP* threshold: 35±3%;

*vglut2a* 24 hpf surface grain: 2, *HV* threshold: 32±3%, *HVP* threshold: 33±5%;

*vglut2a* 48 hpf surface grain: 5, *HV* threshold: 46±2%, *HVP* threshold: 50±4%;

*gad1* 24 hpf surface grain: 4, *HV* threshold: 48±5%, *HVP* threshold: 42±5%;

*gad1* 48 hpf surface grain: 4/5, *HV* threshold: 39±5%, *HVP* threshold: 36±5%.

### Live imaging

mRNA for cellular markers was generated *in vitro* using the mMESSAGE mMACHINE™ SP6 Transcription Kit (Thermo Fisher Scientific) and purified using the MEGAclear Transcription Clean-Up Kit (Thermo Fisher Scientific). All embryos were injected with ∼1 nl of solution consisting of mRFP-Caax (40 ng/µl), H2B-mKeima (40 ng/µl) and 0.05% Phenol Red, as previously described (Soto et al., 2020).

Embryos were mounted 1 h before imaging in a 50 mm glass-bottom dish (MatTek Corporation), positioned face-up for a transverse view in 1% LM agarose with MS222 (final concentration 160 ng/ml). After 1 h of setting at RT, the dish was filled with embryo water supplemented with MS222 (final concentration 160 ng/ml) and N-Phenylthiourea (PTU) (0.045% stock, Sigma Aldrich), which was circulated using a peristalsis pump (Harvard Apparatus).

All live imaging was carried out at 28°C on an Upright LSM880 Airyscan microscope in Fast Airyscan mode. We used W Plan-Apochromat 20×/1.0 DIC (UV) VISIR M27 75 mm lens, 3× zoom, image size 139×139 µm, Z: 81-86 and Z size: 38.4-43.4 µm. The three channels were imaged sequentially and the filters used were: Channel 1, BP 420-480+LP 605; Channel 2, BP420-480+BP495-550; Channel 3, BP 420-480+LP 605. The lasers used were: Channel 1, 561 nm (between 2% and 10% for mRFP); Channel 2, 458 nm (between 6% and 10% for mKeima); Channel 3, 514 nm (between 6% and 13% for Venus). Images were captured every 6 min for 6-12 h. The Airyscan files were processed using the automatic Airyscan processing tool of the ZEN black software.

### DAPT treatment and imaging

Previous work has described a wide range of DAPT concentrations (5-100 mM) (Arslanova et al., 2010; Li et al., 2014). For our studies, we identified an optimal concentration of 40 mM and incubation from 6 hpf to 24 hpf to reduce (but not completely lose) the expression of Her6 protein; this reduction was confirmed by imaging fluorescence protein intensity. A pool of 5-7 embryos were treated with 40 mM DAPT overnight from 6 hpf. The following morning, the chorion was removed and fresh 40-50 mM DAPT was added until 24 hpf; it was an average of 3 h incubation after the chorion was removed. The embryos were always kept at 28°C, except during chorion removal and sorting. At 24 hpf, embryos were fixed with 4% paraformaldehyde for 2 h at room temperature, washed 3×5 min with 1×PBS-Tween and stained with 1 mg/ml DAPI in 1× PBS-Tween and AF647-Phalloidin (Thermo Fisher Scientific, A22287) for 2 h at room temperature. Embryos were imaged within 1-2 days post fixation. They were mounted in 1% agarose and imaged using a confocal Upright LSM880 AiryScan microscope with a W Plan-Apochromat 20×/1.0 DIC (UV) VISIR M27 75 mm lens. Images were collected from fixed 24 hpf *HV* and *HVP* embryos, followed up by measurement of the HV and HVP fluorescent protein intensity.

### Nuclear segmentation in fixed embryos based on DAPI

Individual nuclei were identified manually in 3D based on DAPI intensity. First, using Imaris, the membrane marker (Phalloidin staining) was subtracted from the DAPI signal to ensure good separation between nuclei. Then a 3D Spot region was manually placed at the centre of the DAPI corresponding to each nucleus with a spot size of 2.5 μm diameter in all dimensions. The average signal corresponding to spot regions was exported in the Venus and DAPI channel.

### Nuclear segmentation and single cell tracking from live imaging data

We used the Spots tool on Imaris 9.3.1 to automatically identify cells in the domain of interest based on the nuclear marker H2B-mKeima. The automated detection was checked manually for Venus- or H2B-mKeima-expressing nuclei that may be missed due to the signal quality. For the population analysis, both Her6:Venus and H2B-mKeima mean fluorescence intensities were extracted from these cells from selected time points, starting from the first frame of imaging at 20 hpf and continuing at 2 h intervals up to 26 hpf. To investigate whether Her6 expression is downregulated over the course of development, we quantified the relative trend in each time-series by dividing the last intensity value by the first (relative trend ratio) (Fig. 2F).

For distinguishing between Venus (+) and Venus (−), a threshold was determined for each embryo. For this, we first calculated the median value of background intensity in each individual embryo as a starting threshold. Then we manually examined cells with expression above and below this initial threshold and adjusted it if needed to represent the boundary between visible Venus (+) and Venus (−) cells.

For single-cell traces analysis, single nuclei were tracked and analysed using methods described previously (Soto et al., 2020) using Imaris 9.3.1. In short, we first subtracted the H2B-mKeima signal (Channel 2, nuclear marker) from the Caax-mRFP signal (Channel 1, membrane marker) using the ‘arithmetic tool’ to remove background signal within the boundaries of each cell. The resulting channel (Channel 4) was then subtracted from the Her6:Venus signal (Channel 3) to segment as distinguishable nuclei. We then used the ‘Spots’ (5 µm in *x*, *y* and *z* diameter) and ‘Track over time’ functions to curate tracks of individual cells over time using a combination of automatic and manual tracking. In cases where automatic tracking was used, all final tracks were manually checked to ensure the same cell was tracked over time and there had been no mixing between neighbouring tracks. We also generated background tracks to measure background fluorescence. All statistics were exported from Imaris and processed. All track data were exported from Imaris 9.3.1. As the Imaris 9.3.1 version does not distinguish between daughter cells connected to the same mother cell when exporting the intensity values, we used the Track Reconstruction (tRecs) Python script (https://github.com/TMinchington/tRecs) to connect the tracks of daughter cells and re-form the cell families in the exported data. Tracks shorter than 3 h were rejected and for cells that divided only one track was considered (out of the two daughter cells).

### Selection of cell pairs in HVP embryos

From the tracking datasets of *HVP*, we identified for each track the nearest neighbour over time by computing the average Euclidean distance in 3D and selected pairs that stayed less than 15 μm apart on average, from which we show representative examples (Fig. S6C).

### Detection of oscillatory and aperiodic fluctuating activity from time-series

Dynamic analysis of single-cell tracks was performed using the method developed by Phillips et al. (2017) and adapted for zebrafish single cell tracks by Soto et al. (2020). In summary, we first normalised Her6:Venus to the H2B-mKeima signal by division (Fig. 2Giii; HV/H2B Normalised) to correct for any fluctuations in Her6:Venus that were caused by global changes in transcription and translation or technical issues during image acquisition. Next, as the analysis pipeline is most accurate in detecting oscillators in absence of long-term trends (Phillips et al., 2017), we detrended the Her6:Venus/H2B-mKeima from long-term trends of 4.5 h (Fig. 2Giv; HV/H2B normalised-detrended), a recommended value representing three times the expected period (Phillips et al., 2017) as estimated in Her6 hindbrain progenitors (Soto et al., 2020).

The detection method uses two competing Gaussian Process covariance models corresponding to either random aperiodic fluctuating (OU) or noisy periodic wave (OUosc). During the analysis, the experimental time-series data is used first to infer optimal parameters for both models separately and the probability of the data under each model is computed. Time-series collected from background are used to calibrate technical noise. The confidence in the data being oscillatory is expressed as a log likelihood ratio (LLR) of the probability of the data under the oscillatory model over the non-oscillatory model. To select the LLR threshold for classifying oscillators and controlling the FDR, a set of synthetic data is generated from the OU model and synthetic aperiodic LLR scores are calculated.

By comparing the LLR score from the non-oscillatory synthetic data with LLR from the experimental data, we selected the LLR threshold suitable for our data with the stringent FDR of 3%. In order to ensure a fair statistical comparison, the datasets were paired in FDR as well as statistical testing. Specifically, in the comparison of HV/H2B versus H2B-mKeima (Fig. 2) and the comparison of HV to HVP (Fig. 3) the corresponding datasets were analysed as paired. This produced a similar percentage oscillation in HV (Fig. 2J and Fig. 3F). The aperiodic technical control percentage H2B-mKeima passing at the same LLR threshold was ∼5% across all pairings. Although the imposed FDR is 3% in the synthetic data, a 5% passing rate of the technical control signal H2B is within accepted tolerance values.

For the amplitude of fluctuations and oscillations, peaks and troughs in the HV/H2B normalised tracks were identified using the Hilbert transform applied to detrended HV/H2B. Subsequent peaks and troughs were paired and their position was used to identify corresponding intensities in the raw HV/H2B signal and calculate fold-change (peak/trough) in the raw data (Manning et al., 2019). We report maximum peak-to-trough fold-change per track throughout.

### Coherence of power spectra

We used frequency analysis to investigate how oscillatory each time-series is by quantifying coherence (Alonso et al., 2007). For each time-series, we generated the periodogram and then computed coherence as the area under the curve situated in a region close to the dominant frequency (10% either side of the peak) over the total area of the periodogram. This tracks the contribution of the dominant frequency in explaining the data, with values close to 1 corresponding to a perfect wave.

### Intensity analysis from fixed point nuclear detection of neighbouring cells

The 3D coordinates of each detected nucleus in the cell population snapshot of selected time points were used to measure the inter-nuclear distance at ∼2.3 µm with no differences between HV (2.314±0.1326 µm) and HVP (2.343±0.1388 µm). The closest neighbour to each nucleus was identified based on minimal 3D Euclidean distance and the intensity levels of Venus and mKeima were stored in paired datasets referred to as cell-cell intensity distributions. Some signal variability was associated with *z*-depth due to imaging artefacts or other uncharacterised expression gradients. To circumvent their effect on the analysis, ratiometric analysis was performed where the ratio between mean fluorescence of each selected nucleus was calculated in relation to its nearest neighbour in each embryo, referred to as cell-cell intensity ratios.

### Telencephalon dissection and RNA extraction

To dissect the telencephalon, a Petri dish was coated with 1% agarose in embryo water and, once set, punctured with a 10 µl pipette tip to generate small holes. Then 24 or 48 hpf embryos were anaesthetised with MS222 and placed in the small holes generated on the agarose plate to prevent them from moving.

Embryos were visualised with intermediate contrast on a stereo microscope and the telencephalon was dissected using a Gastromaster microdissection machine at the highest settings. The dissected tissue was transferred with a 10 µl pipette tip coated with low-melting (LM) agarose (to prevent it from sticking to the plastic) into 500 µl of ice-cold Trizol. Each sample contained 20 dissected telencephalons.

For RNA extraction, tissues were dissociated by pipetting. We added 100 µl of chloroform to each sample and incubated at RT for 5 min. Samples were centrifuged for 15 min at 21,000 ***g*** and supernatant was transferred to a fresh tube. Then 0.5 µl of Glycoblue (Invitrogen) was added to facilitate RNA precipitation and visualisation, along with 250 µl of isopropanol. Samples were incubated at −20°C overnight. They were then centrifuged at 21,000× ***g*** for 1 h and supernatant was removed. The pellet was washed with 500 µl of 70% ethanol and centrifuged for 5 min at 21,000 ***g***. Pellets were air dried for ∼5 min at RT and resuspended in 7-10 µl of RNAse free water (Invitrogen).

### Quantitative PCR

RT-qPCR was used to have an understanding of levels of gene expression and HCR was used to estimate the volume of the gene in the study. To remove any potential DNA contamination, RNA samples were first treated with RQ1 RNase-Free DNase (Promega) system according to the manufacturer's instructions. cDNA was generated using SuperScript III Reverse Transcriptase kit (Invitrogen). For qPCR reaction, the probes shown in Table S5 were used with TaqMan Universal PCR Master Mix (Applied Biosystems) according to the manufacturer's instructions. Then 5 ng of cDNA was used in a 5 µl reaction and each sample was run with two technical repeats. RT-qPCR was carried out using a 96-well StepOnePlus Real-Time PCR System with quantitation (comparative CT) and TaqMan experimental setup as a standard run.

CT values were analysed as follows. The technical repeats for each sample were checked to ensure there were no differences larger than 1 unit and were then averaged for each gene per sample. *actb1* (a highly expressed gene and an established qPCR control) and *tmem50a* [recommended housekeeping gene for comparing zebrafish developmental stages (Xu et al., 2016)] were used as controls. The CT values for both housekeeping genes in each sample were averaged to give a single housekeeping CT value. ΔCT was calculated by subtracting housekeeping CT from the CT value of each gene of interest. ΔΔCT was calculated as 2^ΔCT^.

### Cycloheximide chase experiments in MCF7 cells

Human breast cancer MCF7 cells (purchased from ATCC and regularly screened for mycoplasma contamination) were transfected with Lipofectamine 3000 (Invitrogen) according to the manufacturer's instructions. We plated 25,000-35,000 cells in one quarter of a four-quarter glass bottom dish (Greiner) in DMEM media (Sigma Aldrich) supplemented with 10% fetal bovine serum (Gibco). When cells reached 60-70% confluency (often after 24 h) they were transfected with 500 ng HV or HVP plasmid. Two days post transfection, cells were treated with 5 µg cycloheximide (Sigma-Aldrich) and imaged immediately afterwards on an Inverted LSM 880 Airy/FCS Multiphoton (NLO) microscope (Zeiss) with a Plan-Apochromat 20×/0.8 M27 objective and image acquisition every 17 min using tile scanning, for ∼22 h. Two separate detectors were used for high and low Venus levels, as plasmid transfection results in highly variable levels of expression.

Maximum intensity projections of all images were generated using FIJI and data were randomised. Cell tracking was performed blindly on the randomised data on Imaris 9.3.1 using the Spots function with a spot diameter of 11.9 µm. The mean Venus intensity from all spots was extracted and, when signal was saturated with the detector capturing lower levels of Venus expression, the mean intensity from the other detector was used. Cells with saturated signal were excluded.

To estimate the protein half-life, the Venus expression tracks for HV or HVP were pooled together for each biological experiment (*n*=3) and GraphPad Prism 9.3.1 was used to fit one phase decay exponential curve to each dataset.

### Statistical analysis

All data were processed using R-4.1.3. Main R packages used were readr 2.1.2, tibble 3.1.6, dplyr 1.0.8, tidyr 1.2.0, ggplot2 3.3.5 and tidyverse 1.3.1. All statistical analysis was carried out using GraphPad Prism Version 9.3.1. Table S6 gives detail of statistical tests performed, number of biological repeats and total number of cells or embryos per figure. Statistical significance: ns, *P*>0.05; *P≤0.05; **P≤0.01; ***P≤0.001; ****P≤0.0001.

### Quantification of heterogeneity by CV

CV measures the relative variability around the mean in the data. This is a very useful tool for comparing variability in grouped data where different groups do not have comparable means, such as Venus and mKeima or HV and HVP in the context of the present work. CV is calculated by the division of s.d. by the mean. Two types of CV were used: (1) Population CV was obtained by calculating the mean intensity of all cells detected in each snapshot followed by calculating the s.d. of intensities of all cells in that population. Next, the s.d. was divided by the mean for each snapshot, resulting in a single CV value for that snapshot. In cases where several time point snapshots are pooled, the mean, s.d. and CV were calculated for the whole pool. (2) Time-series CV (CV_t_) was obtained by calculating the mean intensity of each single tracked cell over the duration of its time-series as well as the s.d. of all time points. Next, the s.d. was divided by the mean, resulting in time-series CV_t_.

### Mathematical modelling

#### Models 1 and 2: Time delayed stochastic models with and without cell-cell coupling

Models 1 and 2 are based on the same equations, with the only difference being that Model 1 does not include coupled Her6 dynamics between cells. As Model 1 is contained within Model 2, in this section Model 2 will be described in full first and then the difference between the models will be highlighted at the end of the section.

Model 2 is a multicellular grid of cells that are coupled together via lateral inhibition, representative of Notch-Delta signalling (adapted from Biga et al., 2021). Expression of mRNA and protein are included explicitly, time delays are included and the simulations are stochastic. The equations that describe protein and mRNA levels in each cell are:
(1)

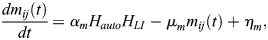

(2)

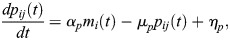
where *m*_*ij*_(*t*) and *p*_*ij*_(*t*) are the mRNA and protein abundance in a cell on row *i* and column *j* of the simulated grid of cells. *α*_*m*_ and *α*_*p*_ are the transcription and translation rates, respectively, and *μ*_*m*_ and *μ*_*p*_ are the degradation rates of mRNA and protein, respectively. *H*_*auto*_ and *H*_*LI*_ are the autoinhibition and lateral inhibition Hill functions that in full are:
(3)

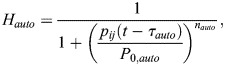

(4)

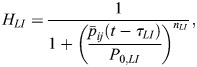
where *τ*_*auto*_ and *τ*_*LI*_ are the time delays associated with Her6 autoinhibition and lateral inhibition, respectively. *P*_0,*auto*_ and *P*_0,*LI*_ are the repression thresholds of the Hill functions, and *n*_*auto*_ and *n*_*LI*_ are the Hill coefficients. 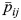 is the average protein in the adjacent neighbouring cells:
(5)

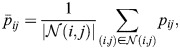
where 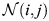 is the set of neighbours a cell is in signalling contact with and 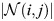 is the total number of neighbours in the set.

The terms *η*_*m*_ and *η*_*p*_ in Eqns 1 and 2 are the stochastic noise terms for mRNA and protein, which have been implemented as a chemical Langevin set of equations, meaning that these terms are Gaussian white noise scaled by the square root of the number of events that occur in each process:
(6)

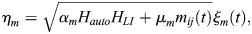

(7)

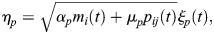
where *ξ*_*m*_(*t*) and *ξ*_*p*_(*t*) are Gaussian white noise with mean of 0 and variance of 1. Equations were solved using the Euler-Maruyama method. All parameter values and ranges used in the simulations are defined in Table S1.

The key difference in Model 1 is that it does not include the lateral inhibition coupling Hill function given by Eqn 4 – the rest of the equations remain the same.

### Optimiser implementation

As most of the parameter values in the model are not known from experimental data in the zebrafish telencephalon, we opted to define a range of reasonable parameter values based on the literature and explored model behaviour within these ranges (Table S1 gives all parameter ranges used). Efficient exploration of parameter space was achieved by implementing the models within the objective function of a pattern search optimiser (Audet and Dennis, 2002) and setting the optimiser to find parameter sets that lead to an increase in heterogeneity when protein degradation is increased. The MATLAB in-built pattern search algorithm was used as this is a global optimiser that can work on non-smooth error functions. Heterogeneity was measured by the population CV, which is defined below.

Each run of the optimiser consisted of the CV value being compared at 1× degradation and 1.1× degradation for the same parameter set; this 10% change in degradation rate roughly reflects the 12% observed change in the cycloheximide half-life experiments performed in MCF7 cells. The objective function error value being optimised was defined as:

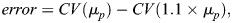
where *μ*_*p*_ is the protein degradation rate. The optimiser was set to minimise the error because the more negative the error value, the larger the increase in expression heterogeneity across the population as a result of increasing the degradation rate. The optimiser was run 6000 times from random initial parameters and was limited to a maximum of 500 function evaluations per run. The parameter sets were subsequently filtered to only include sufficiently negative error values to ensure definite changes in CV were taking place (error<−0.25) and a minimum level of Her6 expression was required to ensure a reasonable expression level (>2000 protein abundance). Each individual run of the model was 100 h long, with time steps of 1 min, and a grid of cells 10 rows by 6 columns was used – the approximate dimensions of one side of the telencephalon.

### CV calculation in the model

For all summary statistics calculated from the model, the final 50% of the time-series were used. Simulation times were 100 h total, and so the summary statistics represent the model behaviour from the last 50 h hours of the simulations. This was to ensure dynamics reached a dynamic equilibrium and reduced the influence of initial conditions.

Population CV was calculated for each parameter set as 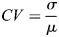, where *σ* and *μ* are the s.d. and mean expression calculated from all the simulated protein expression data from all cells and time points (the whole population), respectively.

In addition, CV_t_ was calculated as 
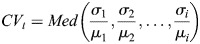
, where *σ*_*i*_ and *μ*_*i*_ are the s.d. and mean expression calculated using all time points from cell *i*, and Med () is the median.

### Temporal period calculation in the model

Given a time-series of protein expression ***x***(*t*)=(*x*_1_, *x*_2_, …, *x*_*t*_), the wavelet transform *W*(***x***)(*f*, *t*), where *f* is frequency, and *t* is time, can be used to determine the dominant period at each time point. The dominant period is defined here as the one that has the highest power, where power is calculated as 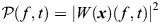. Temporal period for individual cells was calculated using the continuous wavelet transform MATLAB function, which uses Morse wavelets.

For statistical testing a bootstrapping method was used. This involved randomly permuting ***x*** to give ***x***_*rand*_, which is expected to have no periodic behaviour. The distribution of power values in the wavelet transform of ***x***_*rand*_ was then used to calculate a significance threshold by taking all time points at a given frequency and determining the upper 95% confidence interval of power. Then, if 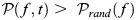, detected periods can be accepted as genuine in the signal ***x***. The average dominant period is calculated by taking the mean of the significant periods detected within a single cell. For a given parameter set, the median value of all cells is taken.

## Supplementary Material



10.1242/develop.202640_sup1 Supplementary information
