# Supplementary material for: Cell coupling compensates for changes in single-cell Her6 dynamics and provides phenotypic robustness
Source: Development. 2024 May 20;151(10):dev202640. doi: 10.1242/dev.202640 (PMC11190438; doi:10.1242/dev.202640)
Supplement: Supplementary information [file develop-151-202640-s1.pdf]

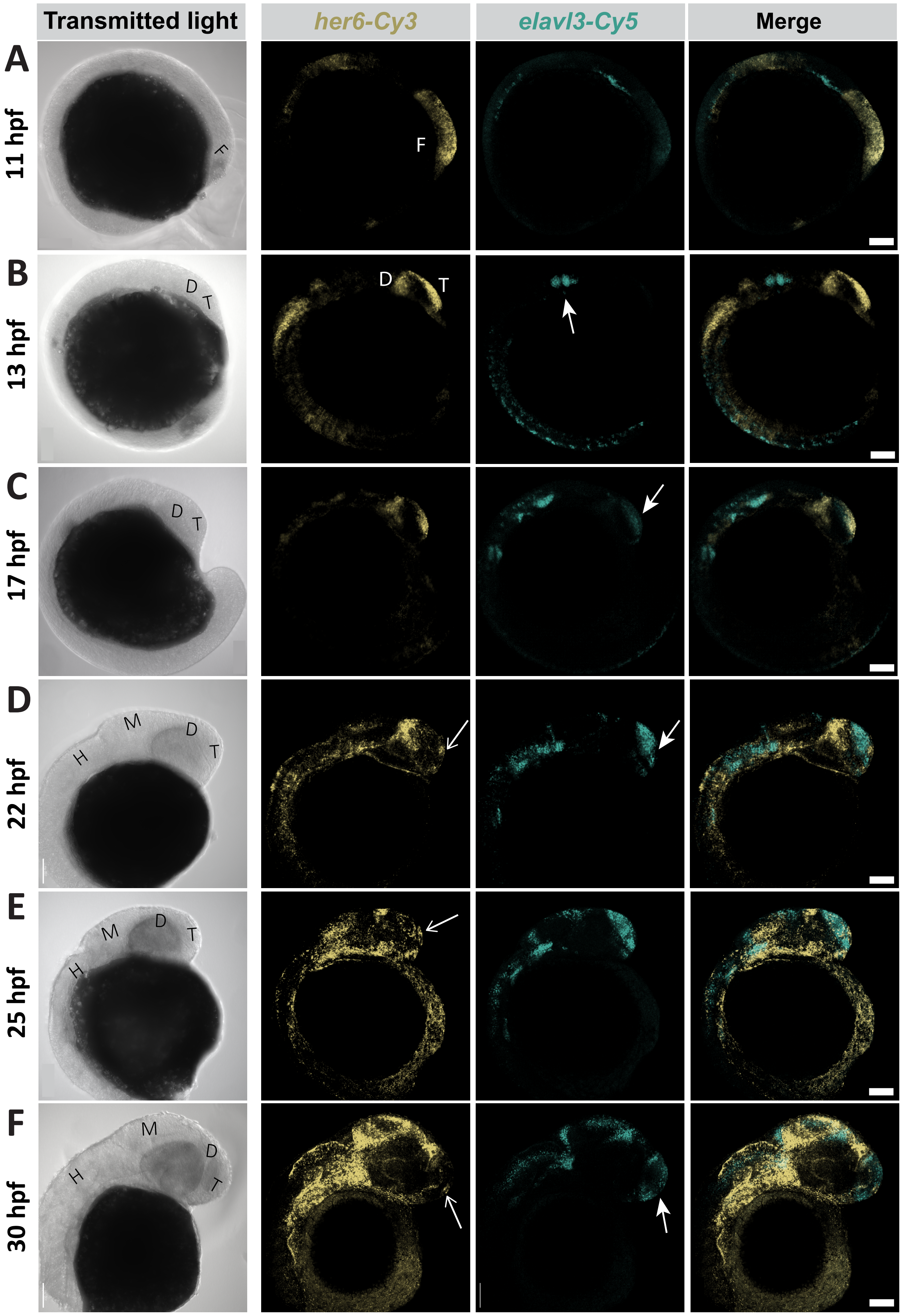

Development • Supplementary information

Fig. S1. Progression of *her6* mRNA expression relative to *elavl3* in the developing zebrafish forebrain (Related to Figure 1). Lateral view of wild type (WT) zebrafish brain development between 11-30 hours post-fertilisation (hpf). Maximum intensity projection of fluorescent in situ hybridization (FISH) against endogenous *her6* and *elavl3* expression; Rostral on the right and caudal on the left.

(A) At 11hpf, *her6* is expressed in presumptive forebrain where there is little to no *elavl3* expression.

(B) At 13hpf, the diencephalon and the telencephalon start to separate and *her6* is expressed in both; no *elavl3* expression is present in the diencephalon or the telencephalon but expression is starting to appear in more caudal regions (arrow).

(C) At 17hpf, the diencephalon and the telencephalon are fully separated and *her6* continues to be expressed in both; *elavl3* starts to appear at this stage in telencephalon (arrow) and strengthens in the more caudal regions.

(D) By 22hpf, the *her6* domain in the telencephalon is reduced and localised to the rostral tip (thin arrow) while *elavl3* expression strengthens in the telencephalon with the exception of the *her6* expression domain (arrow).

(E) At 25hpf, the *her6* expression in the telencephalon remains localised to the rostral tip (thin arrow) similar to 22hpf while *elavl3* is still strongly expressed across the telencephalon.

(F) By 30hpf, the *her6* expression domain in telencephalon is very small (thin arrow) and not overlapping with *elavl3* (arrow). Abbreviations denote F: Forebrain, D: Diencephalon, T: Telencephalon, M: Midbrain, H: Hindbrain. Scalebar = 100 µm.

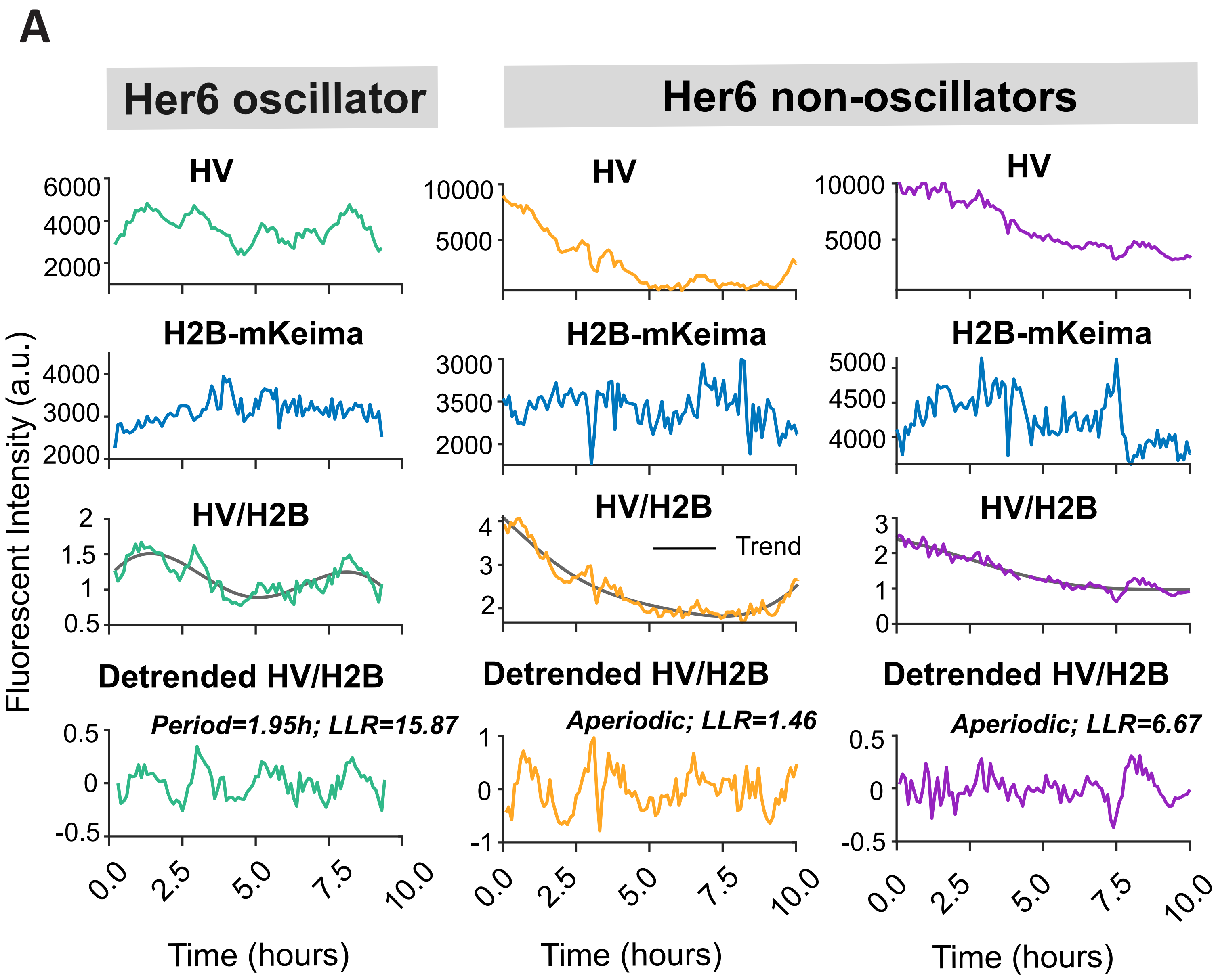
A
Her6 oscillator
Her6 non-oscillators
HV
HV
HV
H2B-mKeima
H2B-mKeima
H2B-mKeima
HV/H2B
HV/H2B
HV/H2B
Trend
Fluorescent Intensity (a.u.)
Detrended HV/H2B
Detrended HV/H2B
Detrended HV/H2B
Period=1.95h; LLR=15.87
Aperiodic; LLR=1.46
Aperiodic; LLR=6.67
Time (hours)
Time (hours)
Time (hours)

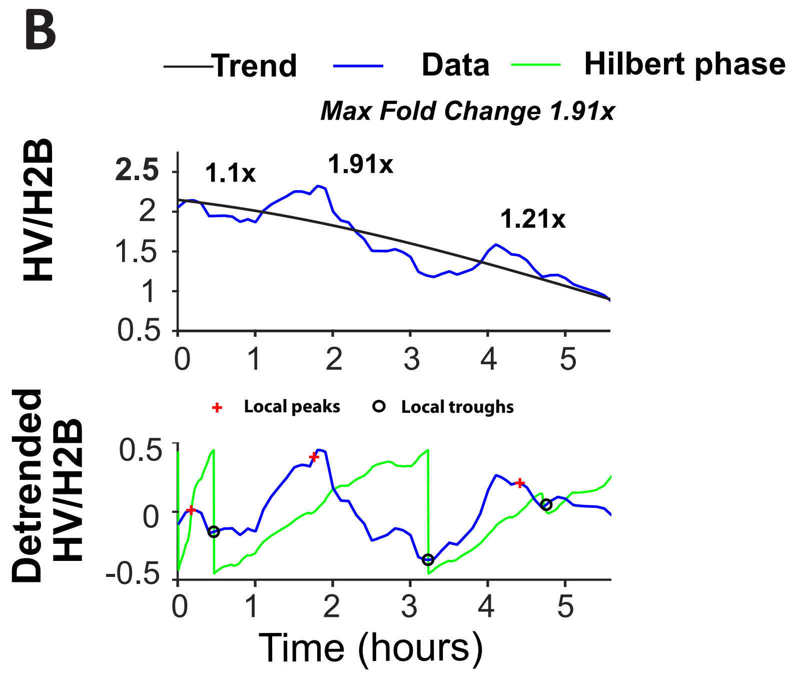
B
Trend
Data
Hilbert phase
Max Fold Change 1.91x
HV/H2B
1.1x
1.91x
1.21x
Local peaks
Local troughs
Detrended HV/H2B
Time (hours)

Fig. S2. Single cell Her6:Venus dynamics observed in *HV* (Related to Figure 2).

(A) Representative examples additional to **Fig. 2G** showing oscillators and non-oscillators detected in *HV* embryos; each row corresponds to a specific cell with columns indicating raw HV, raw nuclear marker H2B-mKeima, HV/H2B and detrended HV/H2B; detrending is performed with Gaussian Processes (Materials and methods) with trend indicated in each example; LLR denotes log-likelihood ratio with high values indicative of periodic activity.

(B) Method of quantifying peak to trough fold change in amplitude; peaks and troughs are identified at the zero-crossing of reconstructed Hilbert phase; sequential peaks and troughs are paired and the maximum peak to trough value is reported.

Development • Supplementary information

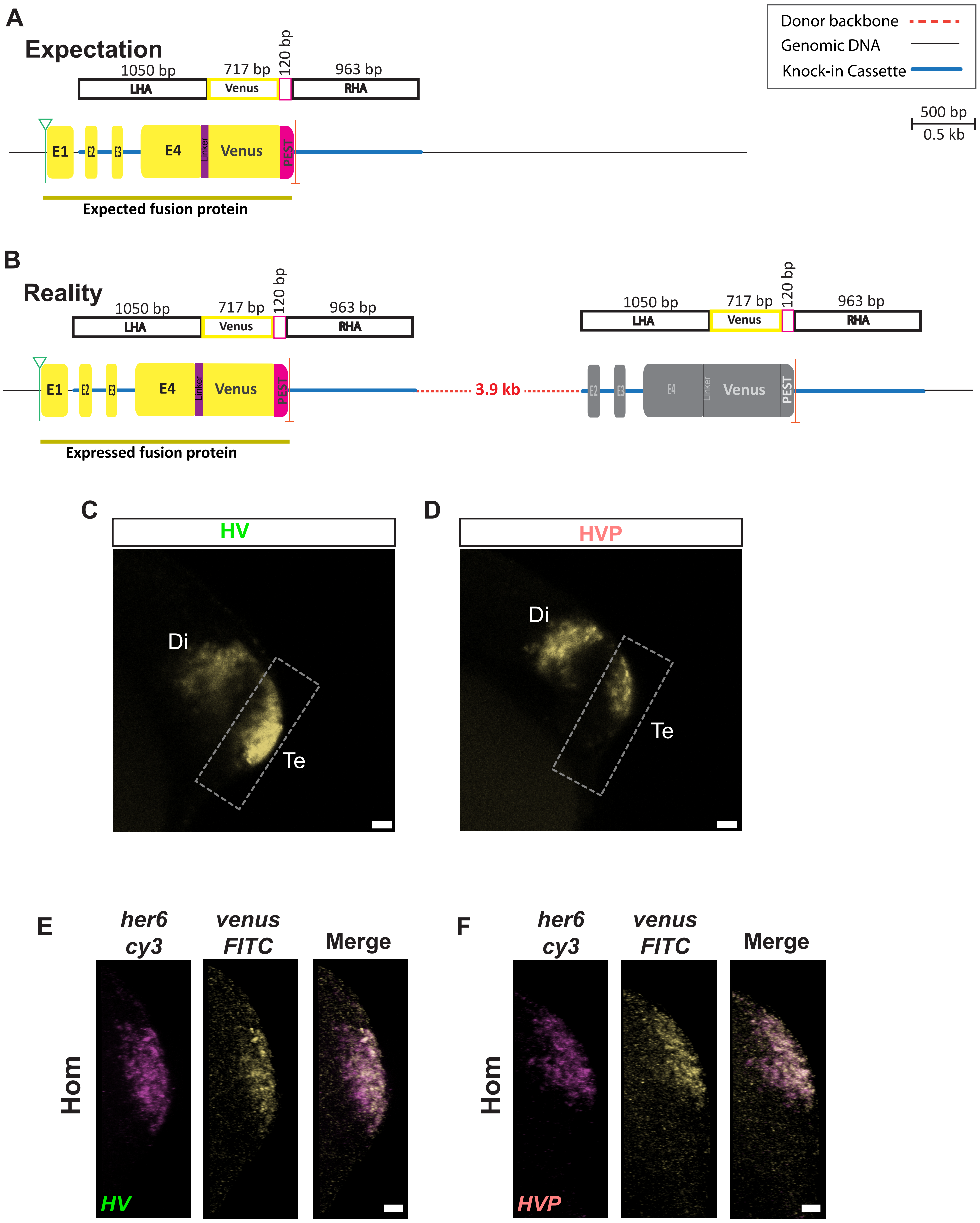

Development • Supplementary information

Fig. S3. The genomic features of the *HVP* and its expression in the telencephalon (Related to Figure 3).

(A) The expected structure of the *HVP* knock-in locus to scale where the LHA-Venus-RHA are inserted in frame with Exon 1; the RHA is followed by zebrafish genomic sequence. RHA: right homology arm; LHA: left homology arm.

(B) The real structure of the *HVP* knock-in locus to scale (except for the donor backbone which is not shown) where the correct expected sequence is present and expected to express the intended fusion protein; the additional ~6.8kb which includes the donor backbone, repeated Exons 2-4, Venus and PEST are not expected to be translated due to the absence of Exon 1 and any other start codons.

(C,D) Confocal images from a single Z plane of live 17-18hpf Hom *HV* and Hom *HVP* knock-in embryos showing expression of Venus in the diencephalon (Di) and the telencephalon (Te); rostral to the right and caudal to the left; scale bar =30µm.

(E) Maximum intensity projection image of WM FISH in 20hpf Hom *HV* embryo showing co-localised expression of *her6* and *venus* mRNA in the Te with no ectopic expression, roughly corresponding to the region outlined in (**C**- dashed box); rostral to the right and caudal to the left; scale bar =20µm.

(F) Maximum intensity projection image of WM FISH in 20hpf Hom *HVP* embryo showing co-localised expression of *her6* and *venus* mRNA in the Te with no ectopic expression, roughly corresponding to the region outlined in (**D**-dashed box); rostral to the right and caudal to the left; scale bar =20µm.

Development • Supplementary information

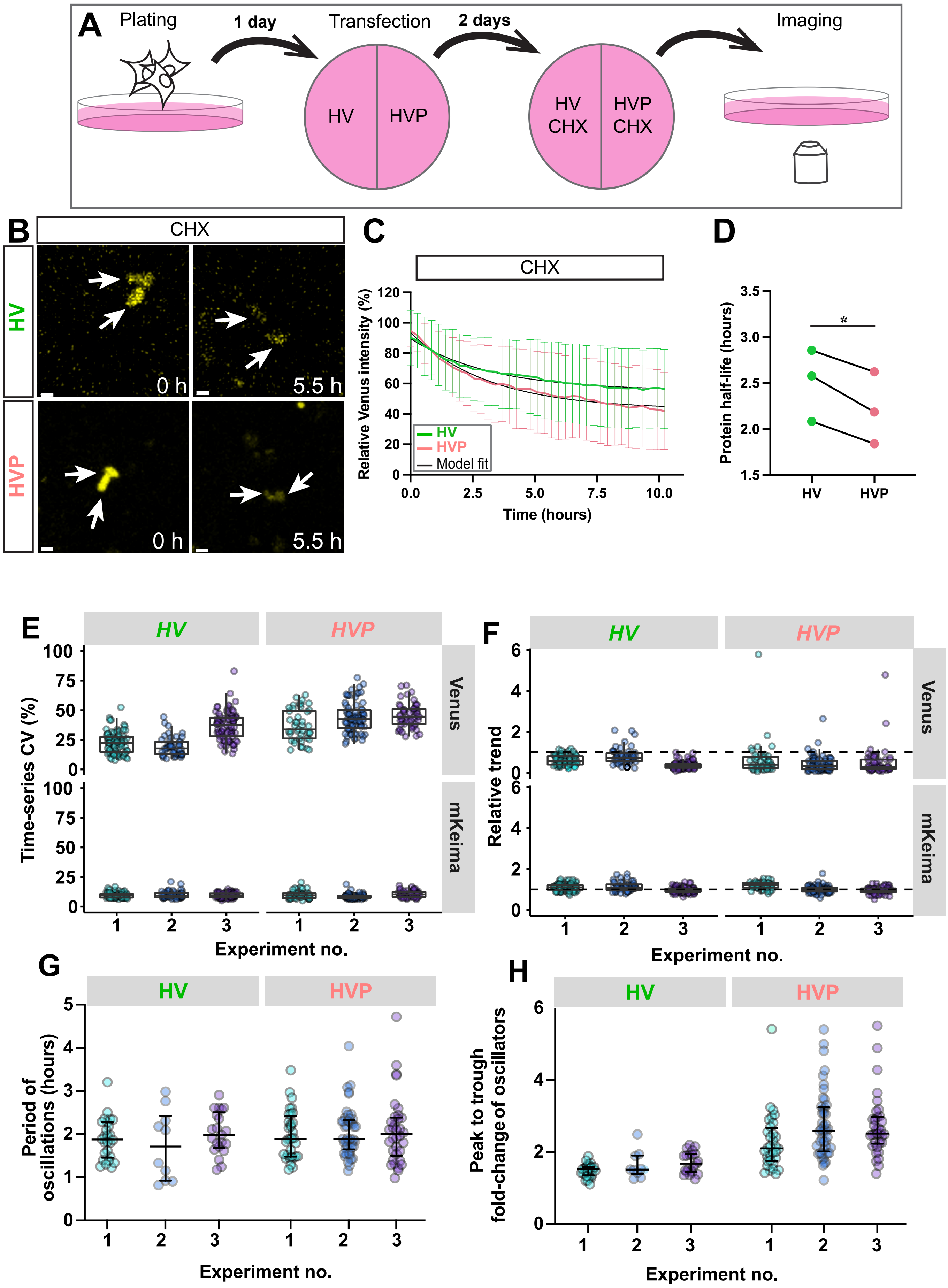
A
Plating
1 day
Transfection
HV
HVP
2 days
HV CHX
HVP CHX
Imaging
B
CHX
HV
HVP
0 h
5.5 h
C
CHX
Relative Venus intensity (%)
Time (hours)
HV
HVP
Model fit
D
Protein half-life (hours)
*
HV
HVP
E
Time-series CV (%)
Venus
mKeima
Experiment no.
F
Relative trend
G
Period of oscillations (hours)
H
Peak to trough fold-change of oscillators

Figure S4. Comparison of HV and HVP half-lives measured by cycloheximide (CHX) chase in transiently transfected MCF7 cells (Related to Figure 3).

(A) The experimental pipeline for CHX chase in MCF7 cells; MCF7 cells were plated and incubated to reach ~60-70% confluency for approximately 24 hours; they were then transfected with either HV or HVP plasmids; 48 hours post transfection, they were treated with CHX and imaged straight after.

(B) Representative images of MCF7 cells transfected with HV or HVP at 0h and 5.5h post treatment with CHX; scale bars = 10µm.

(C) Mean Venus intensity of all HV and HVP MCF7 transfected cells following CHX treatment (n=3 biological repeats, HV=98 cells & HVP=86 cells, error bars represent SD); a one phase exponential decay model was fitted in both data sets; the Venus intensity presented is relative to the highest Venus expression value per track (with the highest expression value corresponding to 100%).

(D) Protein half-life estimation of HV and HVP proteins in MCF7 cells (3 biological repeats, HV=98 cells & HVP=86 cells, HV mean =2.5h, HVP mean = 2.2h, paired two-tailed t-test, *p=0.03).

(E) Comparison of time-series CV for Her6::Venus and H2B-mKeima between HV and HVP; boxes indicate median and interquartile range in each biological repeat; three *HV* and *HVP* pairs (6 embryos) analysed in total; each dot indicates a single cell; sample size *HV* (n=73, 39, 76 cells) and *HVP* (n=40, 65 and 49 cells).

(F) Comparison of time-series relative trend (intensity at last timepoint/intensity at first timepoint) for Her6::Venus and H2B-mKeima between HV and HVP; values > 1 refers to upregulation, values = 1 refers to steady expression and values < 1 refers to downregulation; boxes indicate median and interquartile range in each biological repeat; three *HV* and *HVP* pairs (6 embryos) analysed in total; each dot indicates a single cell; sample size *HV* (73, 39, 76 cells) and *HVP* (40, 65 and 49 cells).

(G) Estimated period in oscillatory HV and HVP; boxes indicate median and interquartile range from 3 biological repeats; each dot represents a single cell; sample size *HV* (26 ,10 and 21 cells) and *HVP* (32, 45 and 34 cells).

(H) Comparison of peak to trough fold-change in oscillatory HV and HVP; boxes indicate median and interquartile range in each biological repeat; three *HV* and *HVP* pairs (6 embryos) analysed in total; each dot represents a single cell; sample size *HV* (n=26 ,10 and 21 cells) and *HVP* (n=32, 45 and 34 cells).

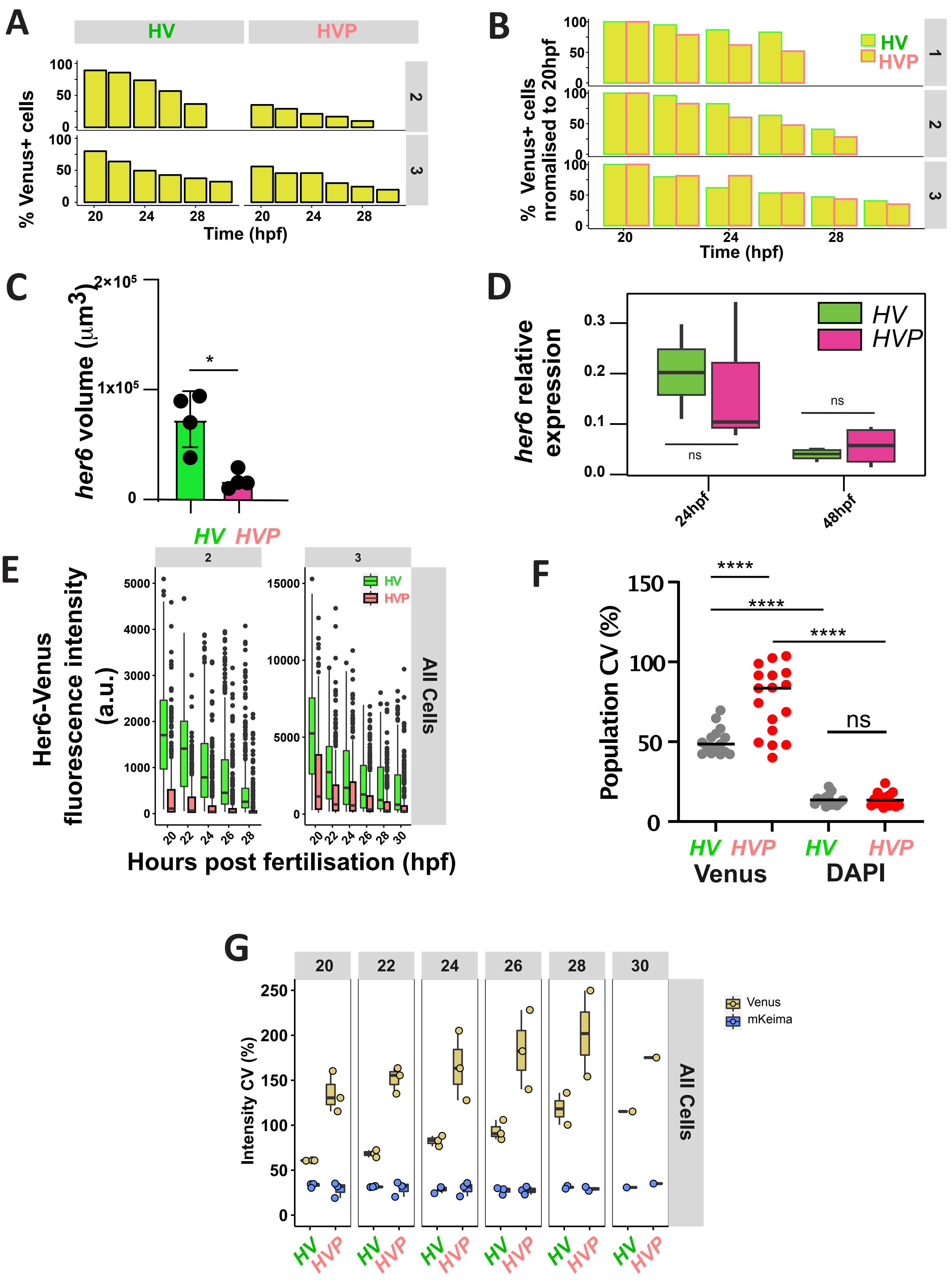
A
HV
HVP
% Venus+ cells
Time (hpf)
B
% Venus+ cells nromalised to 20hpf
Time (hpf)
C
her6 volume (µm3)
HV HVP
D
her6 relative expression
24hpf
48hpf
ns
E
Her6-Venus fluorescence intensity (a.u.)
Hours post fertilisation (hpf)
All Cells
F
Population CV (%)
Venus
DAPI
ns
G
Intensity CV (%)
Venus
mKeima
All Cells

Fig. S5. Exploration of *HV* and *HVP* population heterogeneity in Her6::Venus expression between 20 to 30hpf (Related to Figure 4).

(A) Percentage of Venus(+) nuclei expression observed in HV and HVP over time in two biological repeats (experiment 2: 20-28hpf, experiment 3: 20-30hpf). These are additional to the experiment in **Fig. 4B**.

(B) Percentage of Venus(+) cells observed in HV and HVP over time shown relative to the first time point; bars indicate the percentage of Venus(+) cells from 3 biological repeats; across all repeats, simple linear regression shows no significant difference between the slope of decline in HV and HVP ns: P=0.4072.

(C) 3D volumetric comparison of the *her6* mRNA expression domain in *HV* versus *HVP* embryos. Bars indicate mean and SD, dots indicate domain volume per embryo; unpaired two tailed t-test.

(D) Comparison of *her6* mRNA expression in *HV* (green) and *HVP* (pink) in dissected telencephalon of 24 and 48hpf embryos using qPCR. Boxes indicate median and interquartile range; statistical test is 2-way ANOVA with Sidak multiple comparison correction.

(E) Distributions of nuclear HV and HVP intensities observed in telencephalic progenitors over time from 2 biological repeats additional to the example in **Fig 4C**; boxes indicate median and interquartile range sample size: *HV* (442, 373 and 376 cells) and *HVP* (450, 428 and 297 cells); the dots indicate outliers.

(F) Comparison of population CV computed from Her6:Venus and corresponding DAPI intensities acquired from untreated HV and HVP embryos fixed at 24hpf. Markers indicate embryos; sample size: HV (3 experiments, 15 embryos, 2354 cells) and HVP (3 experiments, 17 embryos, 1358 cells); statistical test is 1 way ANOVA with Sidak multiple comparisons correction. Ns: P=0.9971.

(G) Population CV (Materials and methods) of Venus and H2B-mKeima in HV versus HVP intensity values calculated from data in **(C)**; boxes indicate median and interquartile range of 3 biological repeats; dots represent the mean CV per embryo; statistical testing using mixed-effects analysis with fixed effects (type III) identified: significant differences were observed in HV and HVP versus H2B-mKeima (P=0.0001 and P=0.0017, respectively) and HVP versus HV in all stages (P=0.0122); HV versus H2B-mKeima was significantly influenced by time (P=0.0442) while this was not the case for HVP (P=0.1656).

Development • Supplementary information

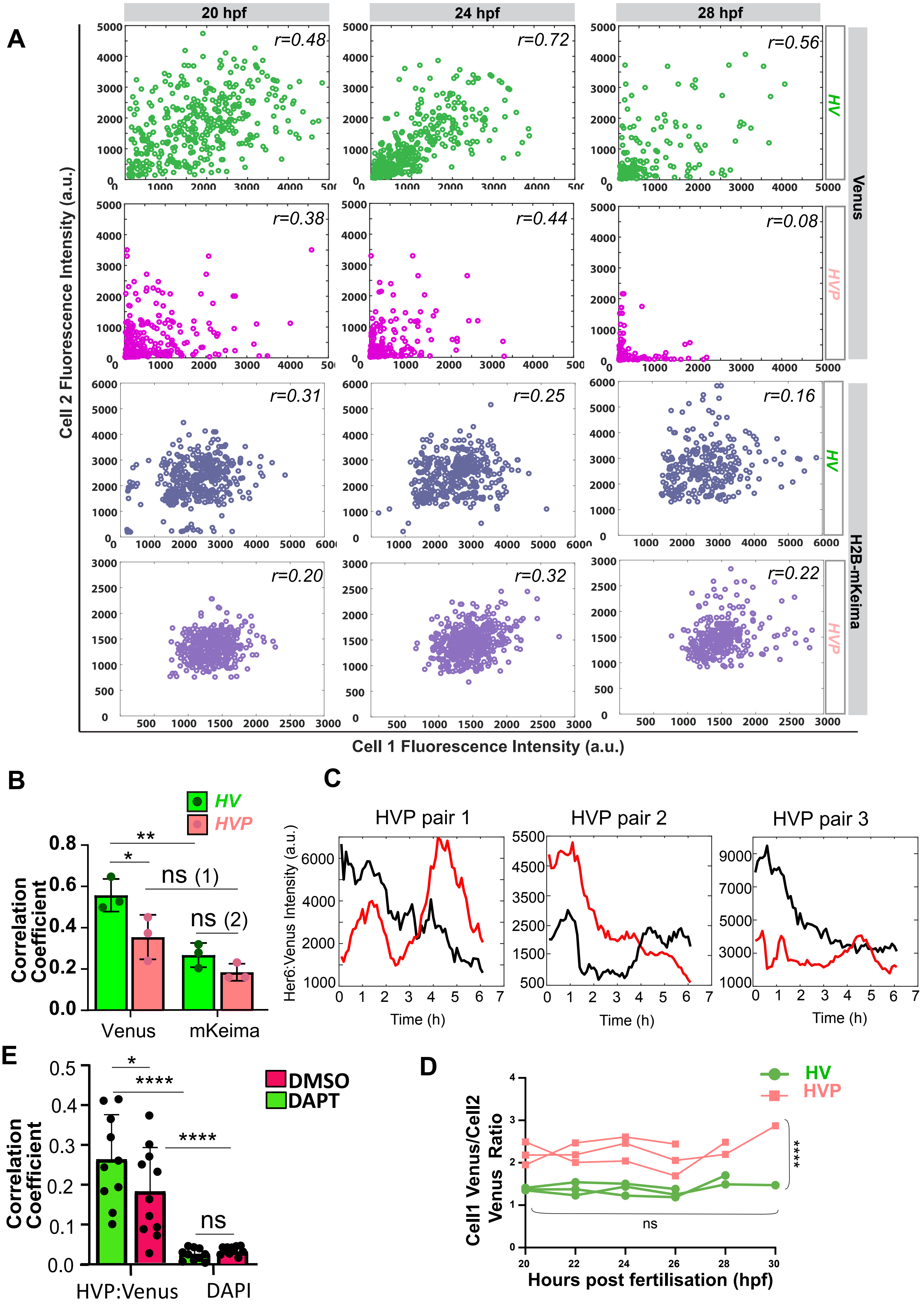

A
20 hpf
24 hpf
28 hpf
r=0.48
r=0.72
r=0.56
r=0.38
r=0.44
r=0.08
r=0.31
r=0.25
r=0.16
r=0.20
r=0.32
r=0.22
HV
HVP
Venus
H2B-mKeima
Cell 2 Fluorescence Intensity (a.u.)
Cell 1 Fluorescence Intensity (a.u.)
B
HV
HVP
Correlation Coefficient
ns (1)
ns (2)
Venus
mKeima
C
HVP pair 1
HVP pair 2
HVP pair 3
Her6:Venus Intensity (a.u.)
Time (h)
E
DMSO
DAPT
Correlation Coefficient
ns
HVP:Venus
DAPI
D
HV
HVP
Cell1 Venus/Cell2 Venus Ratio
ns
Hours post fertilisation (hpf)

Fig. S6. Exploration of cell-cell Her6:Venus intensity relationships in telencephalic tissue compared between HV and HVP (Related to Figure 4).

(A) Local intensity mapping of Her6::Venus (top two rows) and H2B-mKeima (bottom two rows) in neighbouring cells observed in HV and HVP between 20-28hpf in a single experiment; dots indicate intensities in individual nuclei (Cell 1) that were paired with their nearest neighbour (Cell 2) based on 3D Euclidean distance (Materials and Methods); *r* values indicate the Pearson correlation coefficient for each panel.

(B) Pearson correlation coefficient computed from HV local intensity mappings observed between 20-26hpf in *HV* versus *HVP* embryos. Bars indicate mean and SD, dots represent mean per embryo; 3 biological replicates with HV (4892 cells) and HVP (4728 cells); statistical testing is 2-way ANOVA with Tuckey's multiple comparisons. ns (1) P=0.0936 and ns (2) P=0.5588.

(C) Representative examples of Her6:Venus expression in neighbouring *HVP* cells over time.

(D) Average intensity ratios observed in neighbouring cells showing Her6::Venus and H2B-Keima in HVP over time; statistical test is mixed effects (type III) with significant differences in HVP versus corresponding H2B-mKeima (P<0.0001) but no significant changes over time (P=0.3816).

(E) Pearson correlation coefficient computed from local intensity mappings in DAPT versus DMSO conditions (see Fig. 4 **H,I**) observed in HVP embryos fixed at 24hpf. Bars indicate mean and SD, dots represent mean per embryo; statistical testing is 1-way ANOVA with ns: P=0.8901.

Development • Supplementary information

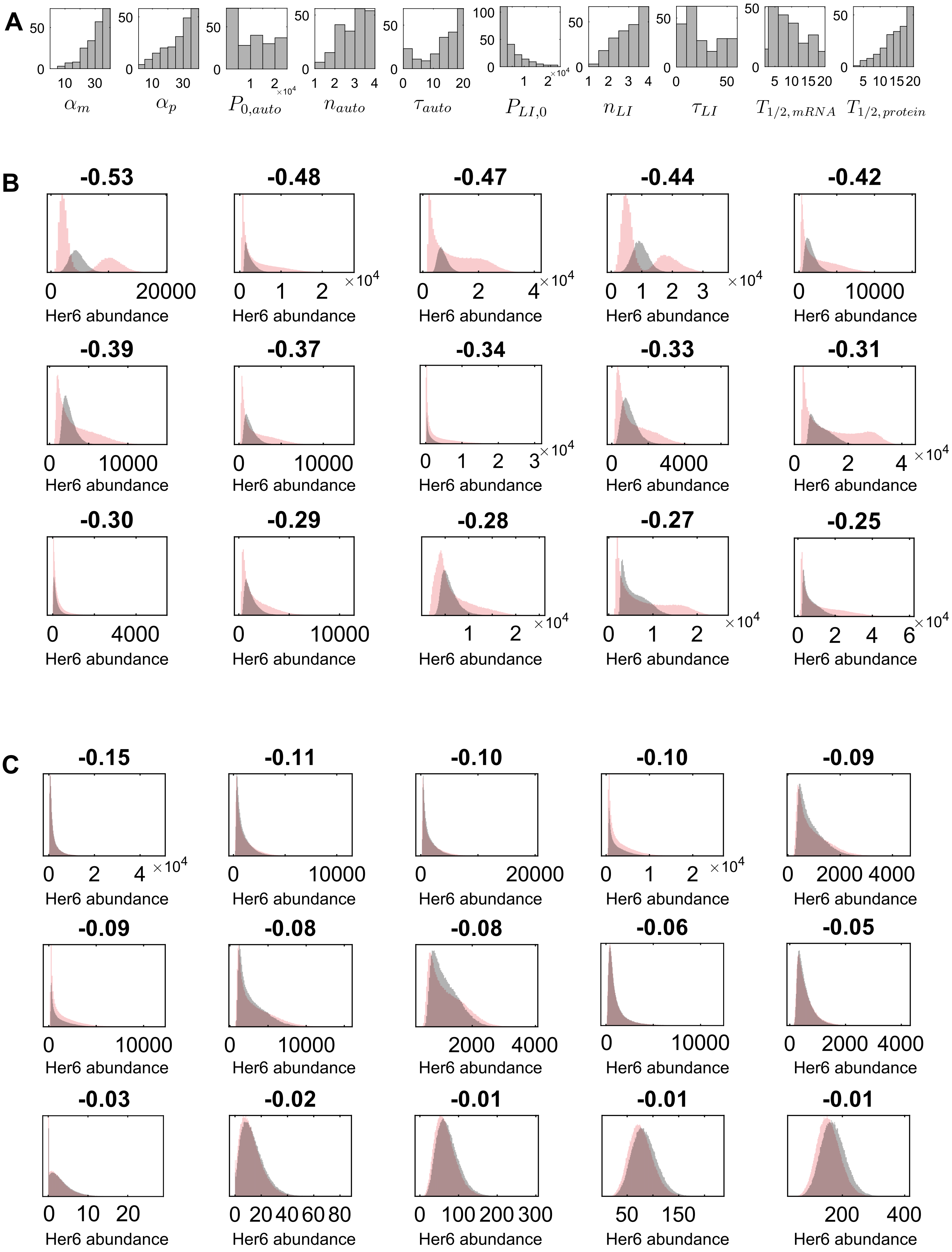

Development • Supplementary information

Fig. S7. Parameters and performance measures from the mathematical models (Related to Figure 5).

(A) Histograms for each model parameter in the 207 accepted parameter sets filtered down from the 6000 runs of the optimiser using *Model 2* (see **Table S1** for biological definitions of all parameters and symbols).

(B) A randomly selected sample of histogram plots from the accepted optimiser parameter sets. Each histogram is a distinct parameter set, where the grey corresponds to the Her6 expression distribution at 1x protein degradation rate, and pink shows 1.1x degradation rate. The histograms are ordered from most negative optimiser error value (where the number above each graph is the error) to the least negative.

(C) A random sample of histogram plots from the unaccepted Model 1 optimiser parameter sets (no parameter sets were accepted). Degradation rates 1x and 1.1x shown in grey and pink respectively. The error rates shown above each graph have values higher than ones in **(B)**.

Development • Supplementary information

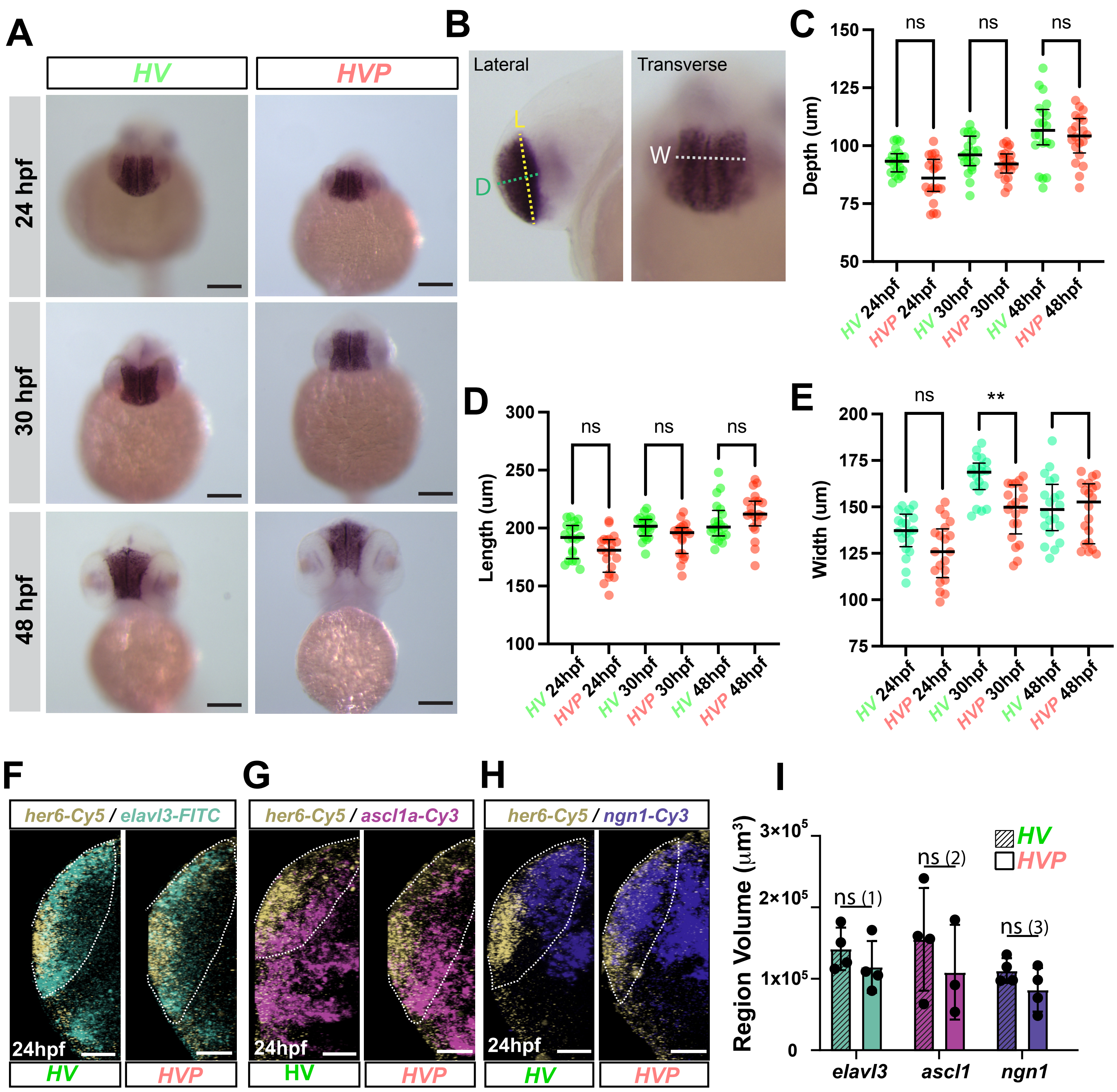

Fig. S8. Comparing gene expression in *HV* and *HVP* telencephalon in a transversal view (Related to Figure 6):

(A) WM chromogenic in-situ hybridisation (ISH) showing *foxg1* mRNA expression in *HV* and *HVP* at 24, 30 and 48hpf in transversal view; scale bars 150µm.

(B) Images denote the measurement of length (L), depth (D) and width (W) of the *foxg1* expression domain in the telencephalon which were used for estimating the telencephalic volume.

Development • Supplementary information

(C-E) Comparison of depth, length and width measured from foxg1 data (**A,B**) in *HV* versus *HVP* at 24, 30 and 48hpf; bars indicate median with interquartile range of 20 embryos per timepoint per condition; statistical test is 2-way ANOVA with Sidak multiple comparison correction testing.

(F-H) Maximum intensity projection image of double whole mount (WM) fluorescent in situ hybridisation (FISH) showing *her6/elavl3***(F)**, *her6/ascl1***(G)** and *her6/ngn1***(H)** mRNA expression in the telencephalon of 24hpf *HV* versus *HVP* embryos; manual annotation denotes telencephalon boundary in transversal view; scale bars 40µm.

(I) 3D volumetric comparison of the *elavl3*, *ascl1* and *ngn1* mRNA expression domain in *HV* versus *HVP* embryos (obtained from data **F-H**). Bars indicate mean and SD, dots indicate domain volume per embryo from 3-4 embryos per condition; statistical testing is 2-way ANOVA with Sidak multiple comparison correction; ns (1): P=0.2000, ns (2) P=0.6286 and ns (3) P= 0.4857.

Table S1. Model parameter values

Symbol	Range explored	Biological definition	Reference
α_m	0.1 – 40min^{-1}	Transcription rate	(Singh and Padgett, 2009; Suter et al, 2011)
α_p	1 – 40 min^{-1}	Translation rate	(Alberts et al., 2008; Boström et al., 1986; Ingolia et al., 2011; Schwanhäusser et al., 2011)
u_m	$ln(2)/2 - ln(2)/20$ min^{-1}	mRNA degradation rate	(Bonev et al., 2012)
u_p	$ln(2)/2 - ln(2)/20$ min^{-1}	Protein degradation rate	(Soto et al., 2020)
$P_{0,auto}$	100 – 25,000 proteins	Her6 autoinhibition repression threshold	(Monk, 2003)
$P_{0,LI}$	1 – 25,000 proteins	LI coupling repression threshold	
n_{auto}	1 – 4	Her6 autoinhibition Hill coefficient	(Galla, 2009; Monk, 2003; Philips et al., 2016)
n_{LI}	1 – 4	Lateral inhibition Hill coefficient	(Collier et al, 1996; Sprinzak et al., 2010; Sprinzak et al, 2011)
τ_{auto}	0 – 20 mins	Her6 autoinhibition time delay	(Lewis, 2003; Monk 2003; Philips et al., 2016)
τ_{LI}	0 – 60 mins	Lateral inhibition time delay	

Development • Supplementary information

Table S2. List of PCR primers

#	Binding location	Direction	Sequence (5'-3')
**1**	3' end of the LHA	Forward	TCCAGTCTACGCAAACAATTCCAAC
**2**	5' end of the RHA	Reverse	ACGCTGAACAAAGAAAACAAGTGTC
**3**	Before the LHA (5' genomic sequence)	Forward	AAACTCCTCTTCTCCGGTCG
**4**	3' end of the RHA	Forward	AGCCTAAGTTCAAAGCAGGT
**5**	3' end of the NeoR/KanR gene (in CRISPR donor vector)	Reverse	AGCCAACGCTATGTCCTGATAGC
**6**	Middle of the NeoR/KanR gene (in CRISPR donor vector)	Forward	TCTTGTCGATCAGGATGATCTGG
**7**	Middle of AmpR gene (in CRISPR donor vector)	Reverse	TTTATCCGCCTCCATCCAGTC
**8**	3'-most end of the CRISPR donor vector	Forward	ATTCATTAATGCAGCTGGCAC
**9**	After the RHA (3' genomic sequence)	Reverse	TCCTGAACTTTTGGCGCTGG
**10**	Middle of LHA	Reverse	ACTTACCGGTCATTTGTGCG

Development • Supplementary information

Table S3. Primers used for amplifying Venus with addition of Eco-RI restriction sites

Description	Direction	Sequence (5'-3')
Venus F with incorporated EcoRI site	Forward	taggagGAATTCGTGAGCAAGGGCGAGGAGCT
Venus R with incorporated EcoRI site without a stop codon	Reverse	accgacGAATTCCTTGTACAGCTCGTCCATGCCG

Table S4. Primers used for amplifying Venus in colony PCR

Name	Direction	Description	Sequence (5'-3')
Venus F	Forward	Internal primer binding in the Venus sequence in forward direction	ACCCTGAAGCTGATCTGCAC
Venus R	Reverse	Internal primer binding in the Venus sequence in reverse direction	GGGTCTTGTAGTTGCCGTCGT

Table S5. Taqman probes used for quantitative PCR

Gene	Symbol	Thermo Fisher Assay ID
actin, beta 1	actb1	Dr03432610_m1
achaete-scute family bHLH transcription factor 1a	ascl1a	Dr03093273_g1
cyclin-dependent kinase inhibitor 1Bb	cdkn1bb (p27)	Dr03101118_m1
distal-less homeobox 5a	dlx5a	Dr03150313_m1
ELAV like neuron-specific RNA binding protein 3	elavl3	Dr03131531_m1
empty spiracles homeobox 3	emx3	Dr03086733_m1
forkhead box G1a	foxg1a	Dr03200829_s1
glutamate decarboxylase 1b	gad1b	Dr03080468_m1
glutamate decarboxylase 2	gad2	Dr03141402_g1
hairy-related 6	her6	Dr03176397_s1
neurogenin 1	neurog 1 (ngn1)	Dr03432616_g1
solute carrier family 17 member 6b	slc17a6b (vglut2a)	Dr03194447_s1
T-box brain transcription factor 1b	tbr1b	Dr03436735_m1
transmembrane protein 50A	tmem50a	Dr03108070_m1

Development • Supplementary information

Table S6. Statistics Summary

§ Statistical significance P >0.05[ns], P < 0.05*, P<0.01**, P < 0.001***, P < 0.0001****.

Figure number	Graph Type	Lines Representing	Biological repeat number	Total cell number	Statistical test and post hoc §
Fig. 2B	boxplot	Median with interquartile	3	1768, 1595, 1529 nuclei per embryo<br>N= 4892	two-way ANOVA with Sidak multiple comparisons
Fig. 2E	boxplot	Median with interquartile	4	244	two-tailed Wilcoxon matched-pairs non-parametric signed rank test
Fig. 2F	scatter dot plot	Median with interquartile	4	244	Mann-Whitney test, unpaired-nonparametric
Fig. 2H	dot plot	Median with interquartile	4	244	Student t-test, paired one-tailed
Fig. 2J-L	dot plot	Median	4	244	Student t-test, paired one-tailed
Fig. 3C	boxplot	Median with interquartile	3	*HV* N=188, *HVP* N=154	Student t-test, paired two-tailed
Fig. 3D	boxplot	Median with interquartile	3	*HV* N=188, *HVP* N=154	Mann-Whitney test unpaired-nonparametric
Fig. 3E	boxplot	Median with interquartile	3	*HV* N=188, *HVP* N=154	Student t-test, paired two-tailed
Fig. 3F	boxplot	Median with interquartile	3	*HV* N=188, *HVP* N=154	Student t-test, paired two-tailed
Fig 3 G	scatter dot plot	Median	3	*HV* N=188*, HVP* N=154	Kruskal-Wallis with Dunn's multiple comparison
Fig. 3H	boxplot	Median with interquartile	3	*HV* N=57, *HVP* N=111	Student t-test, paired two-tailed
Fig. 3I	boxplot	Median with interquartile	3	*HV* N=57, *HVP* N=111	Student t-test, paired two-tailed
Fig. 4C	boxplot	25th to 75th percentile	1	*HV=4892, HVP=4728*	Mann-Whitney, two-tailed
Fig. 4D	Dot plot	Mean	3	*HV* N=4892, *HVP* N=4728	2-way ANOVA with Tukey's multiple comparisons
Fig. 4G	Bar plot	Mean with SD	3	*HV* N=4892, *HVP* N=4728	2-way ANOVA with Tukey's multiple comparisons
Fig. 4J	Bar plot	Mean with SD	*4*	*HV* N=11 embryos*,1241 nuclei*; *DMSO* N=10 embryos, 1632 nuclei	1-way ANOVA with Tukey's multiple comparisons
Fig. 6B	Scatter dot plot	Mean with SD	*HV*=20, *HVP*=20	*HV*=60, *HVP*=60	2-way ANOVA with Sidak multiple comparison correction
Fig. 6C	Dot plot	Mean with SD	*HV*=20, *HVP*=20	*HV*=60, *HVP*=60	2-way ANOVA with Sidak multiple comparison correction
Fig. 6E	Scatter dot plot	Median with 95% CI	*HV*=28, 19, 36<br>*HVP*=29, 33, 30		1-way ANOVA with Kruskal-Wallis multiple comparation
Fig. 6F	Dot blot	Mean with SD	*HV*=28, 19, 36<br>*HVP*=29, 33, 30		1-way ANOVA with Kruskal-Wallis multiple comparation

Fig. 7A-C	boxplot	Median with interquartile	HV: 3, 3<br>HVP: 4, 4		2-way ANOVA with Sidak multiple comparison correction
Fig. 7F	Violin plot		HV: 7, 11<br>HVP: 10, 6		1-way ANOVA with Tukey's multiple comparison correction
Fig. 7G	Violin plot		*HV*: 8, 9<br>*HVP*: 5, 4		1-way ANOVA with Tukey's multiple comparison correction
Fig. 7H-K	boxplot	Median with interquartile	HV: 3, 3<br>HVP: 4, 4		2-way ANOVA with Sidak multiple comparison correction
Fig. 7N	Violin plot		*HV*= 4, 3<br>*HVP*= 5, 4		1-way ANOVA with Tukey's multiple comparison correction
Fig. 70	Violin plot		*HV*= 8, 6<br>*HVP*= 8, 10		1-way ANOVA with Tukey's multiple comparison correction

Development • Supplementary information

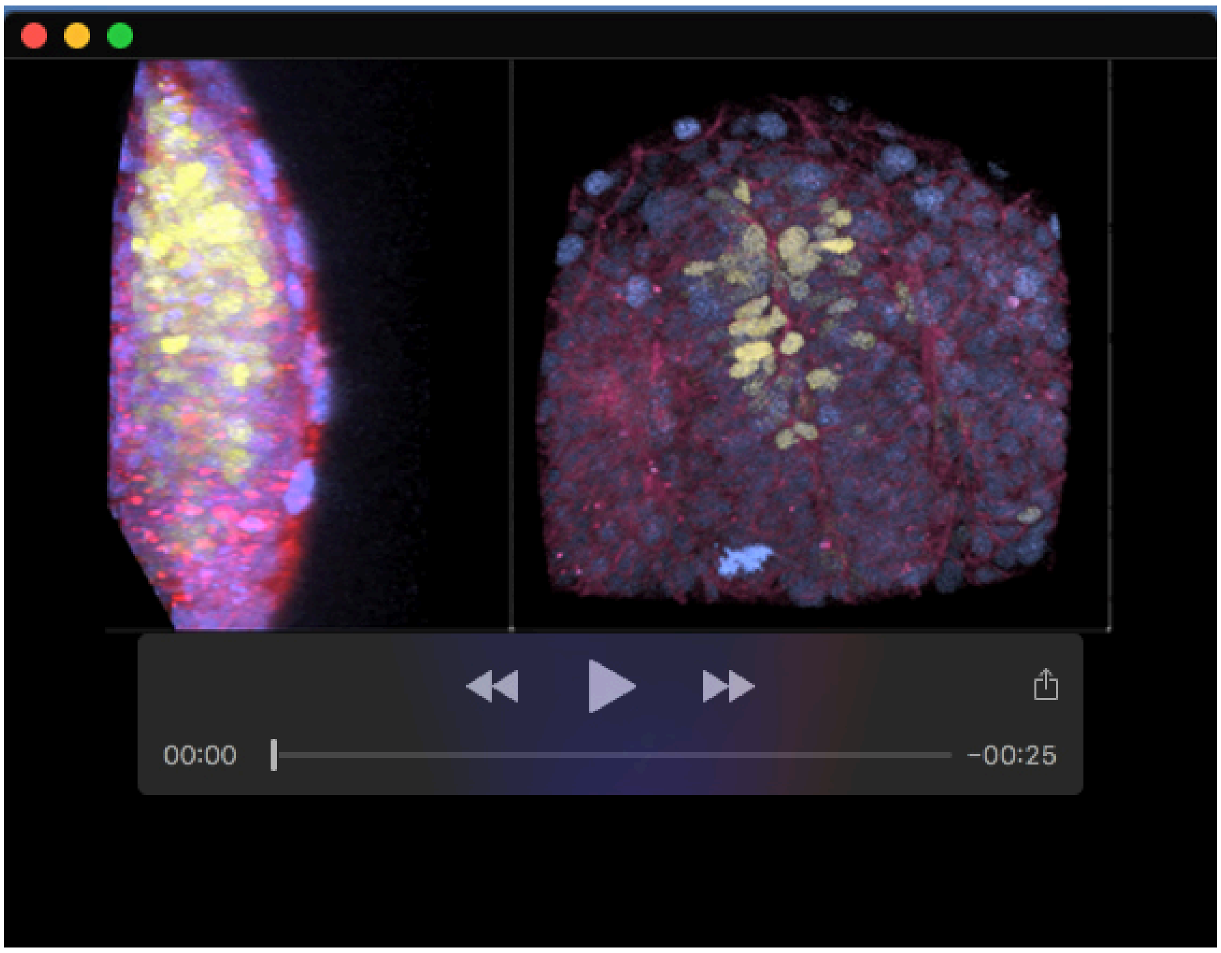

Movie 1. 3D movie of transversal view of the telencephalon in *Her6:Venus* (*HV*) and *Her6:Venus-PEST* (*HVP*) embryos. time point 20hpf. Scale bar 30µm. Video corresponds to still included in **Fig. 4A-20hpf**. Venus is shown in yellow, the nuclear marker H2B-mKeima shown in blue and the membrane markers Caax-RFP shown in red.

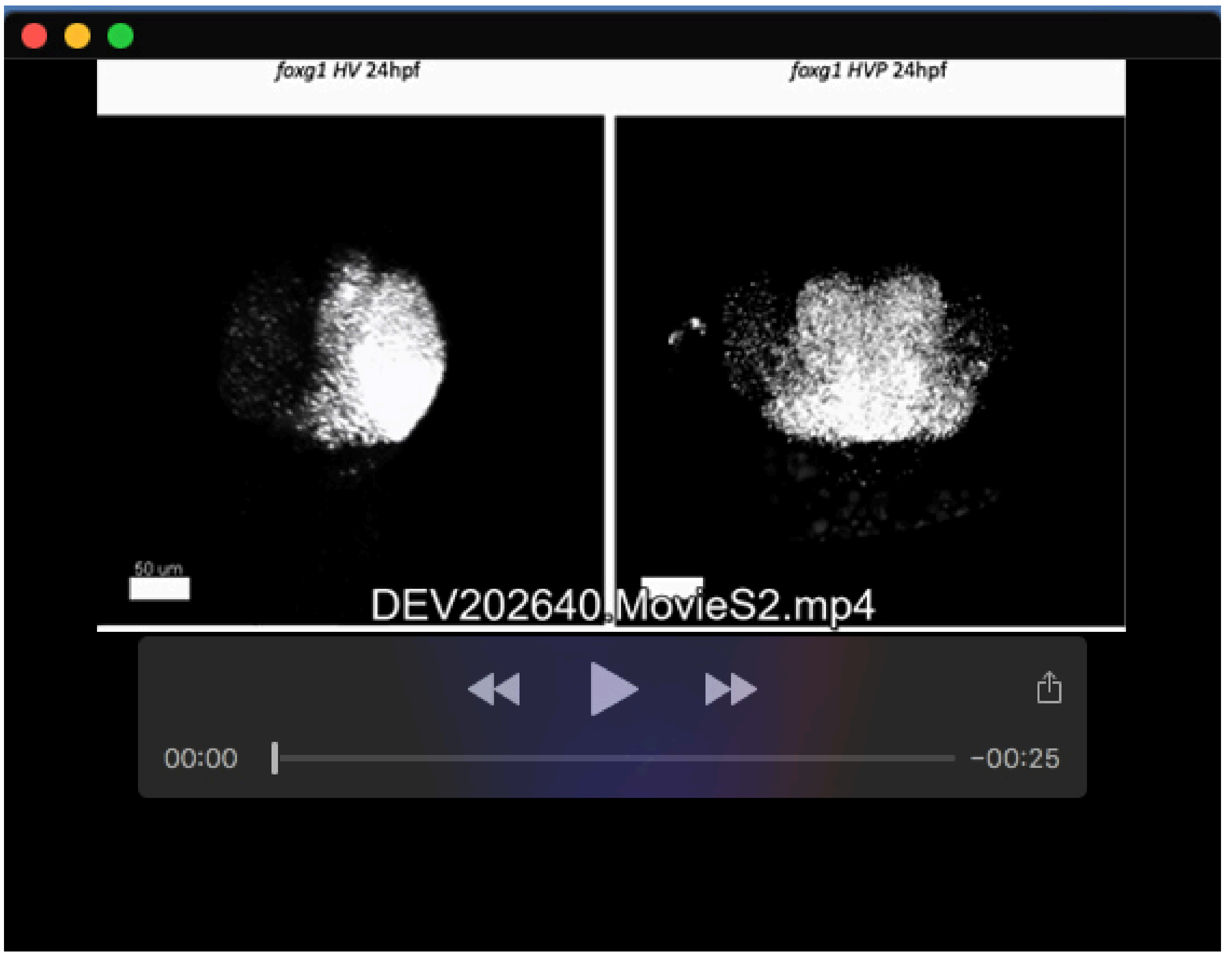

Movie 2. 3D movie of transversal view of the forebrain-telencephalon in *Her6:Venus* (*HV*) and *Her6:Venus-PEST* (*HVP*) embryos at 24hpf, stained for *foxg1* using HCR. Scale bar 50µm. Video corresponds to still included in **Fig. 6D-24hpf.**

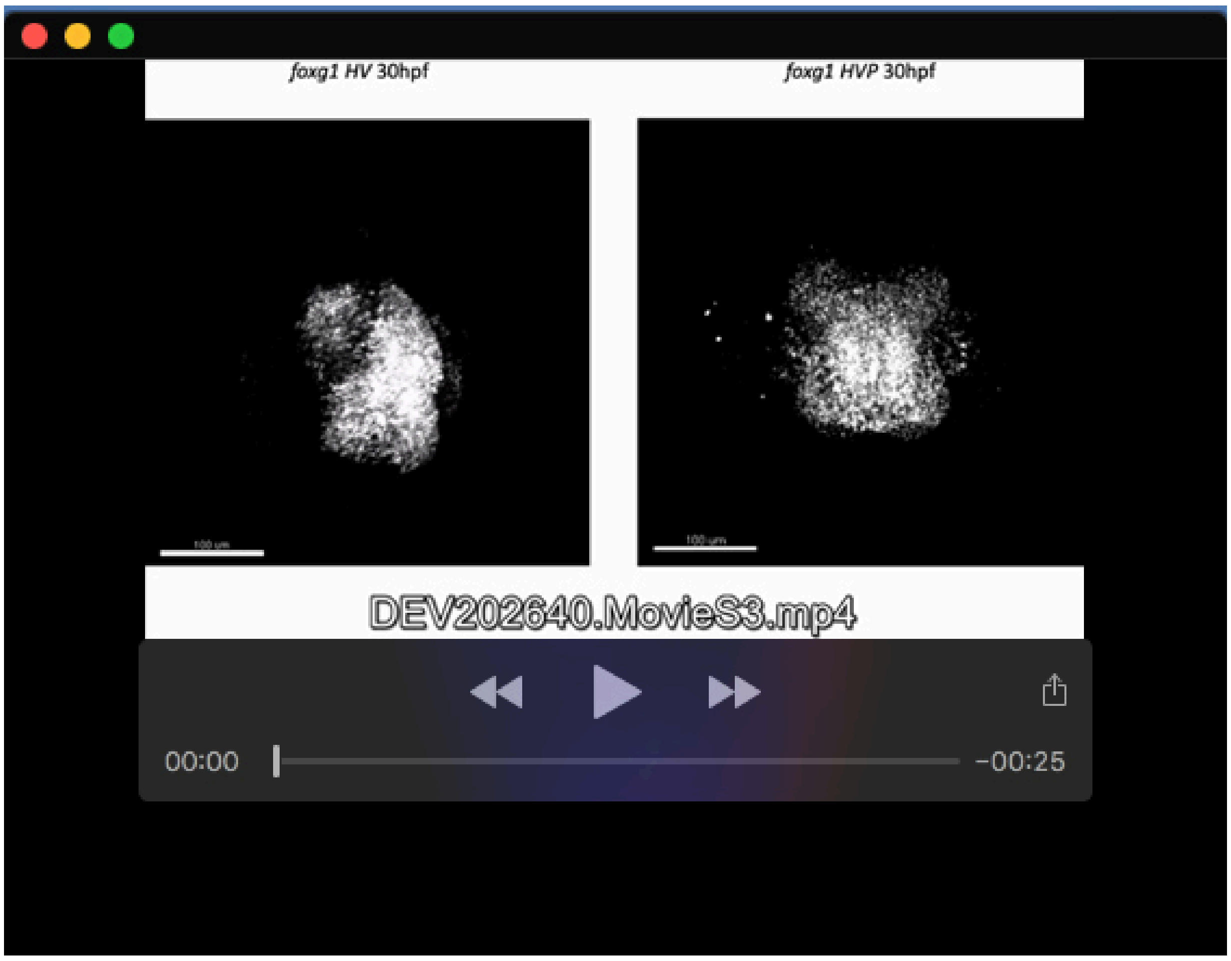

Movie 3. 3D movie of transversal view of the forebrain-telencephalon in *Her6:Venus* (*HV*) and *Her6:Venus-PEST* (*HVP*) embryos at 30hpf, stained for *foxg1* using HCR. Scale bar 50μm. Video corresponds to still included in **Fig. 6D-30hpf.**

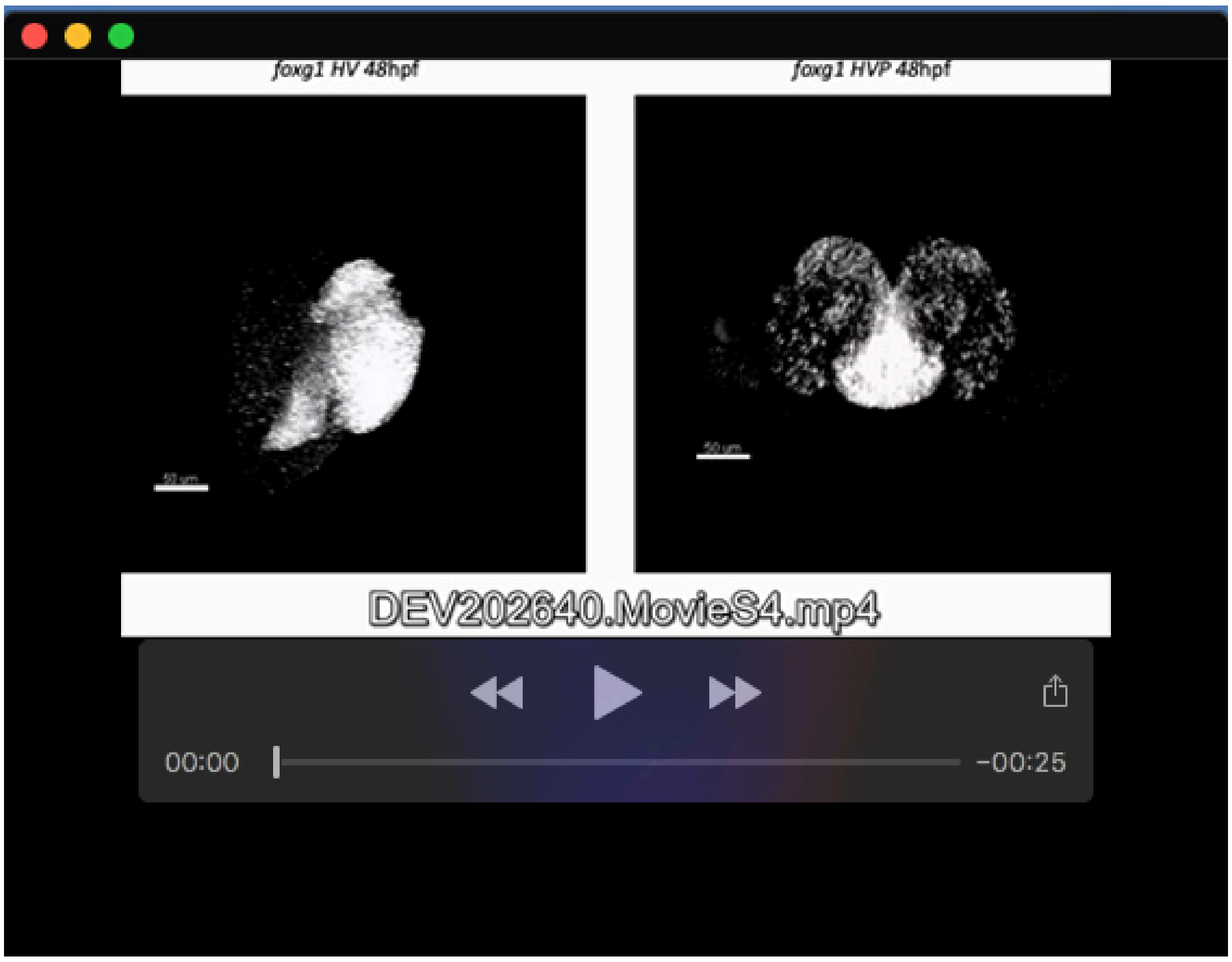

Movie 4. 3D movie of transversal view of the f forebrain-telencephalon in *Her6:Venus* (*HV*) and *Her6:Venus-PEST* (*HVP*) embryos at 48hpf, stained for *foxg1* using HCR. Scale bar 50μm. Video corresponds to still included in **Fig. 6D-48hpf.**

Development • Supplementary information

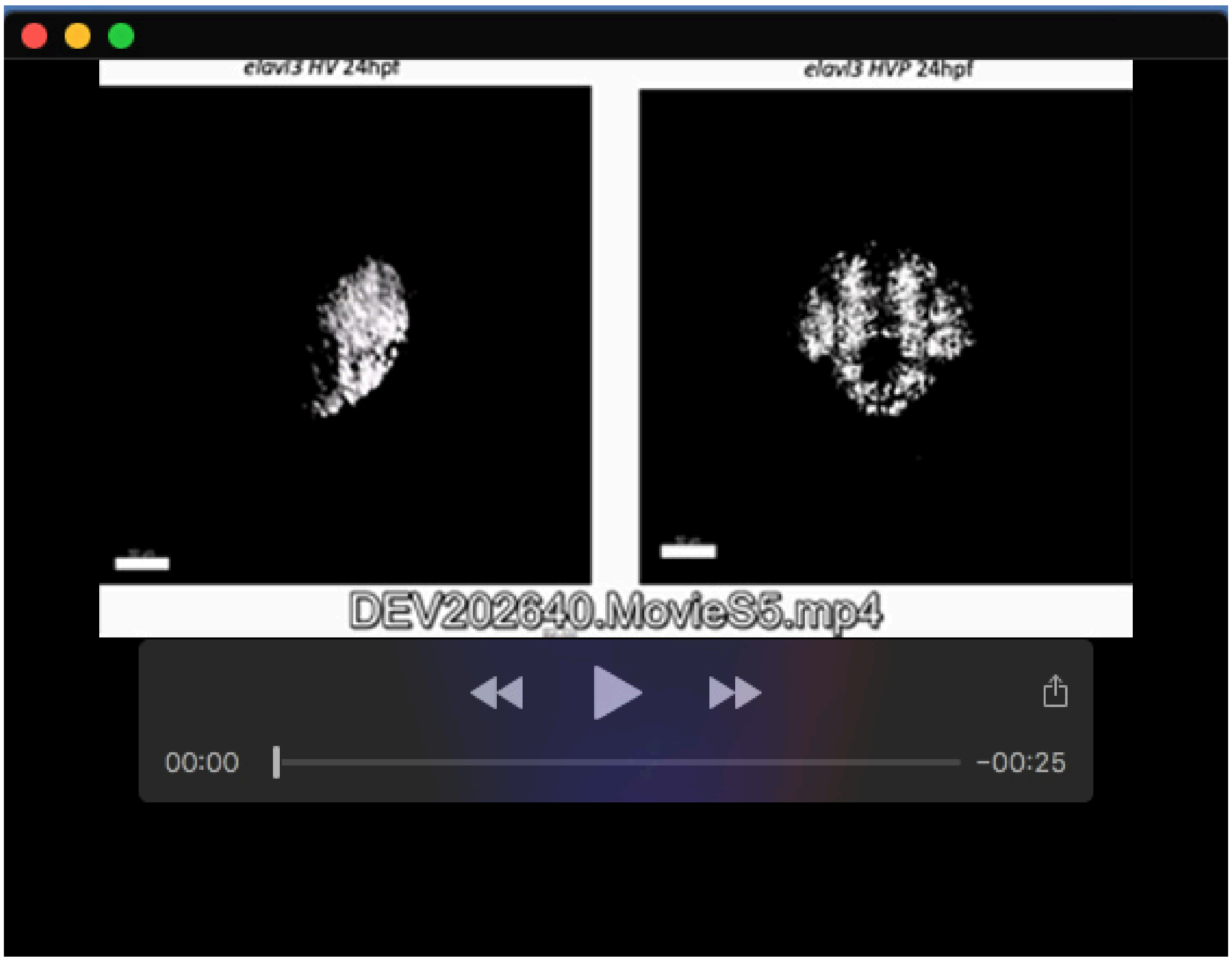

Movie 5. 3D movie of transversal view of the forebrain-telencephalon in *Her6:Venus* (*HV*) and *Her6:Venus-PEST* (*HVP*) embryos at 24hpf, stained for *elavl3* using HCR. Scale bar 50µm. Video corresponds to still included in **Fig. 7D-24hpf.**

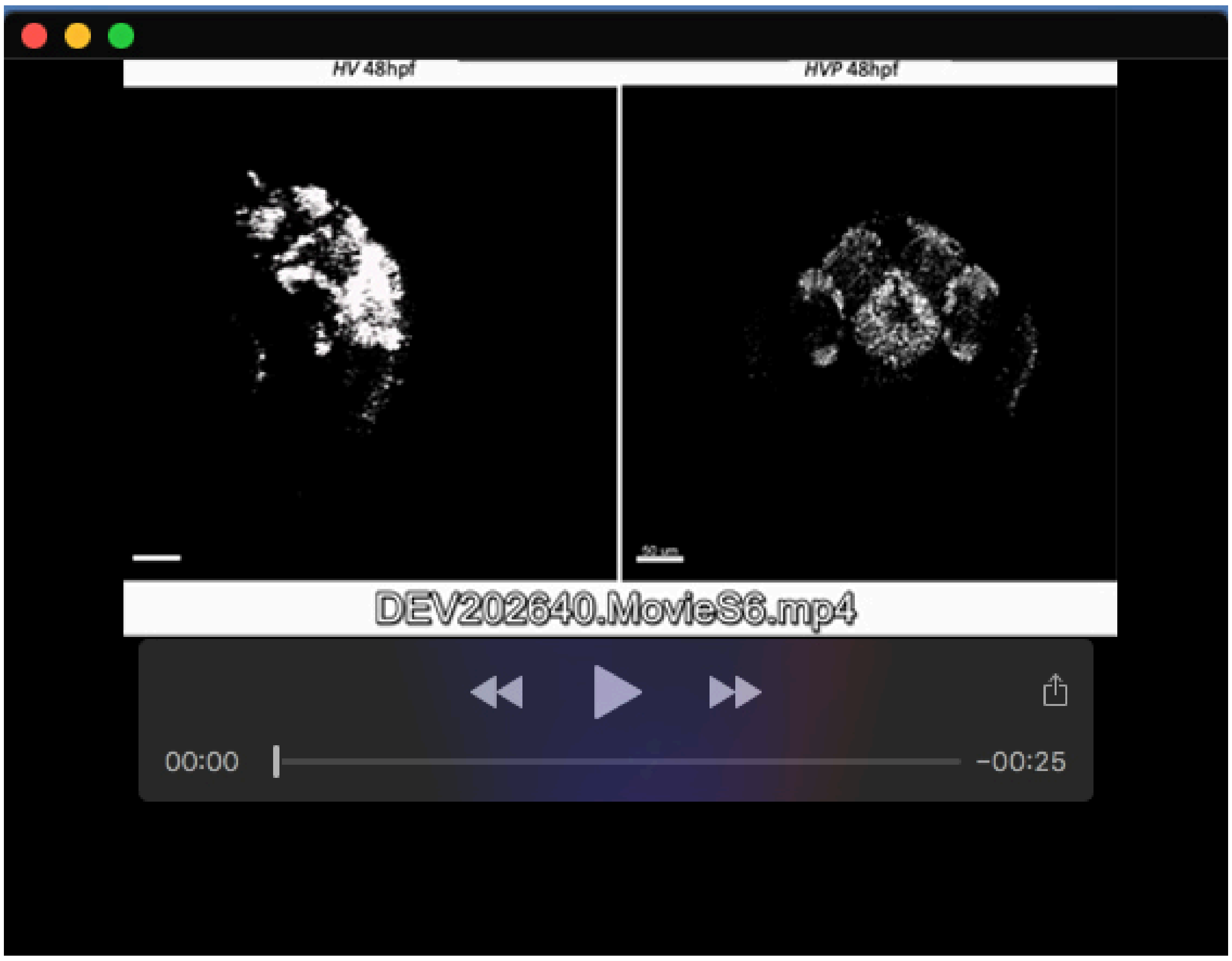

Movie 6. 3D movie of transversal view of the forebrain-telencephalon in *Her6:Venus* (*HV*) and *Her6:Venus-PEST* (*HVP*) embryos at 48hpf, stained for *elavl3* using HCR. Scale bar 50µm. Video corresponds to still included in **Fig. 7D-48hpf**.

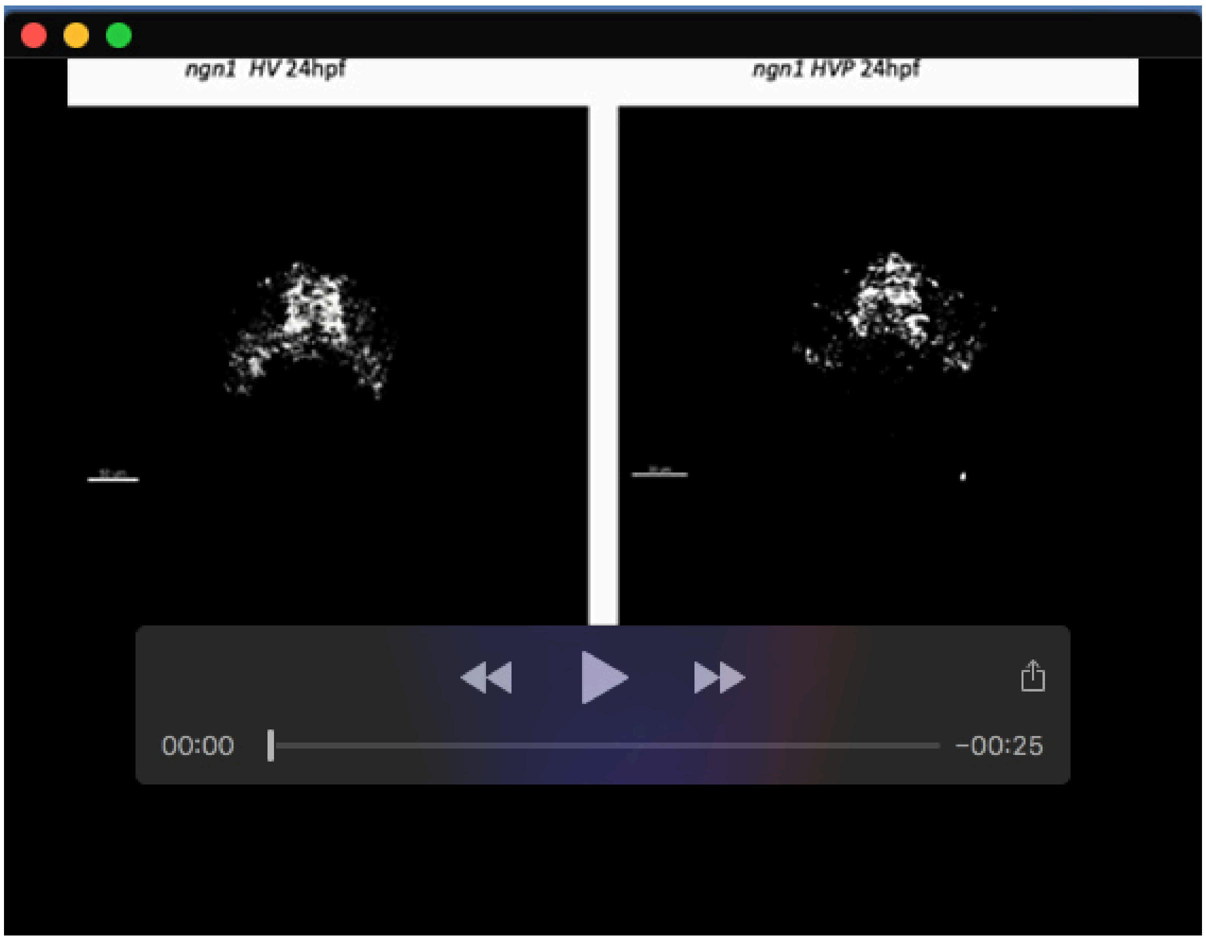

Movie 7. 3D movie of transversal view of the forebrain-telencephalon in *Her6:Venus* (*HV*) and *Her6:Venus-PEST* (*HVP*) embryos at 24hpf, stained for *ngn1* using HCR. Scale bar 50µm. Video corresponds to still included in **Fig. 7E-24hpf**.

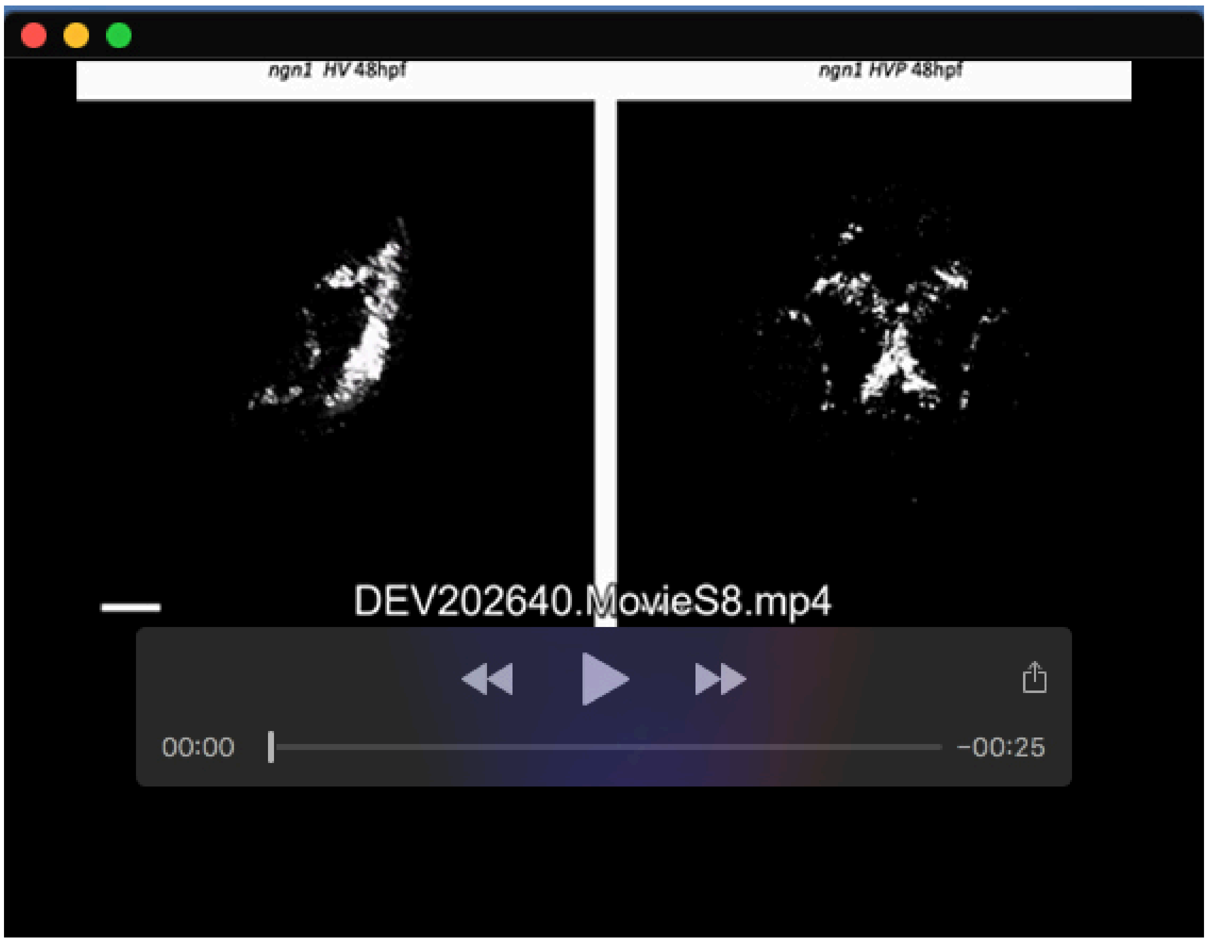

Movie 8. 3D movie of transversal view of the forebrain-telencephalon in *Her6:Venus* (*HV*) and *Her6:Venus-PEST* (*HVP*) embryos at 48hpf, stained for *ngn1* using HCR. Scale bar 50µm. Video corresponds to still included in **Fig. 7E-48hpf**.

Development • Supplementary information

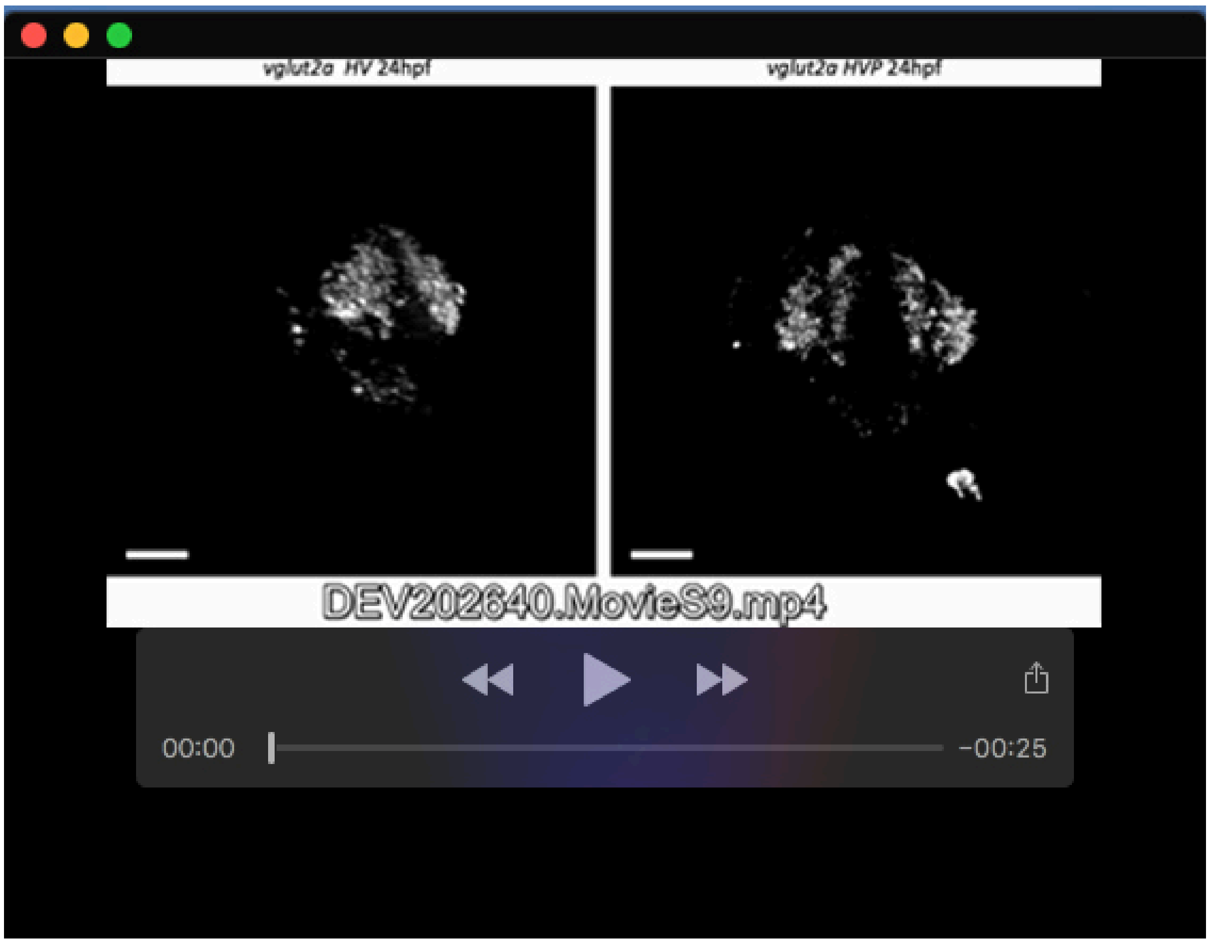

Movie 9. 3D movie of transversal view of the forebrain-telencephalon in *Her6:Venus* (*HV*) and *Her6:Venus-PEST* (*HVP*) embryos at 24hpf, stained for *vglut2a* using HCR. Scale bar 50µm. Video corresponds to still included in **Fig. 7L-24hpf**.

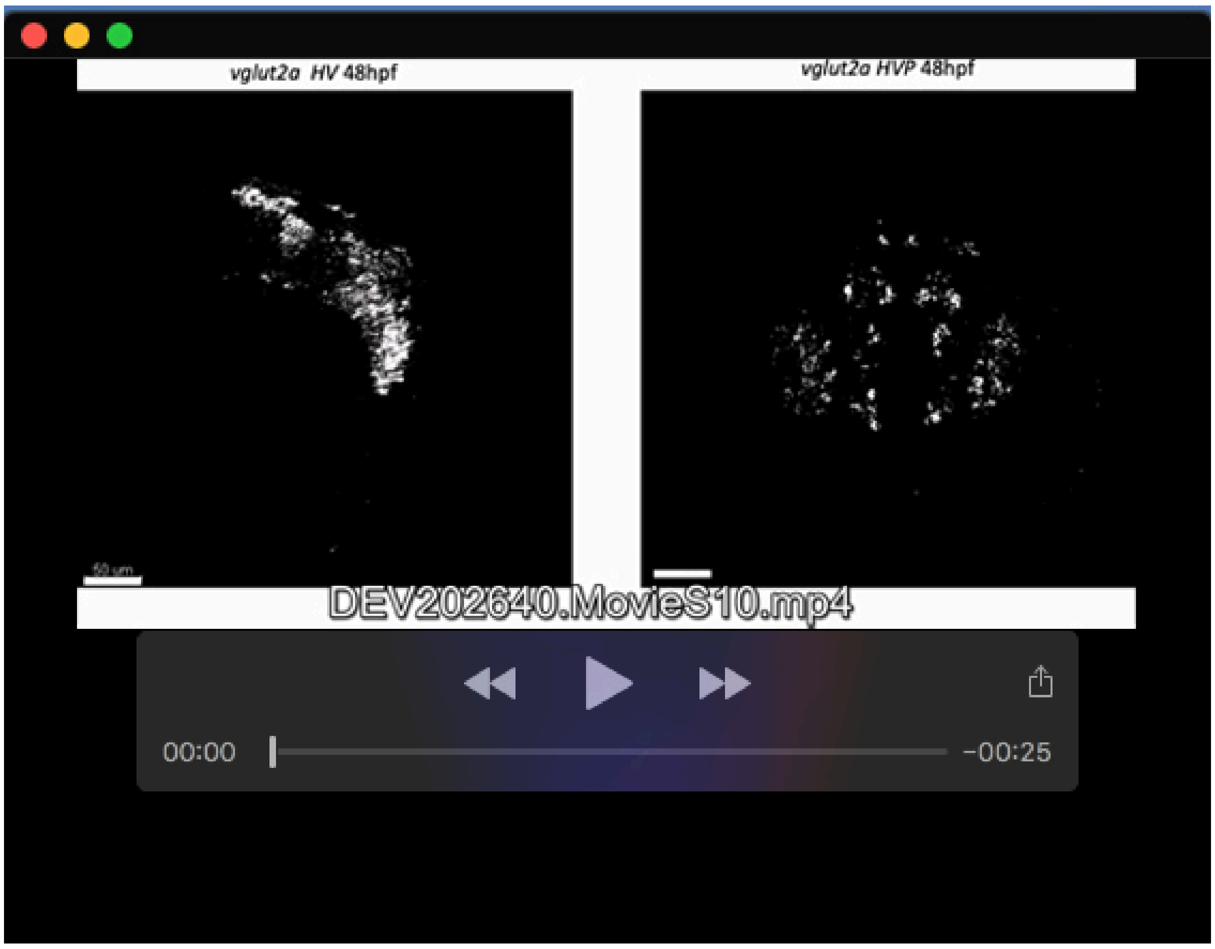

Movie 10. 3D movie of transversal view of the forebrain-telencephalon in *Her6:Venus* (*HV*) and *Her6:Venus-PEST* (*HVP*) embryos at 48hpf, stained for *vglut2a* using HCR. Scale bar 50µm. Video corresponds to still included in **Fig. 7L-48hpf**.

Development • Supplementary information

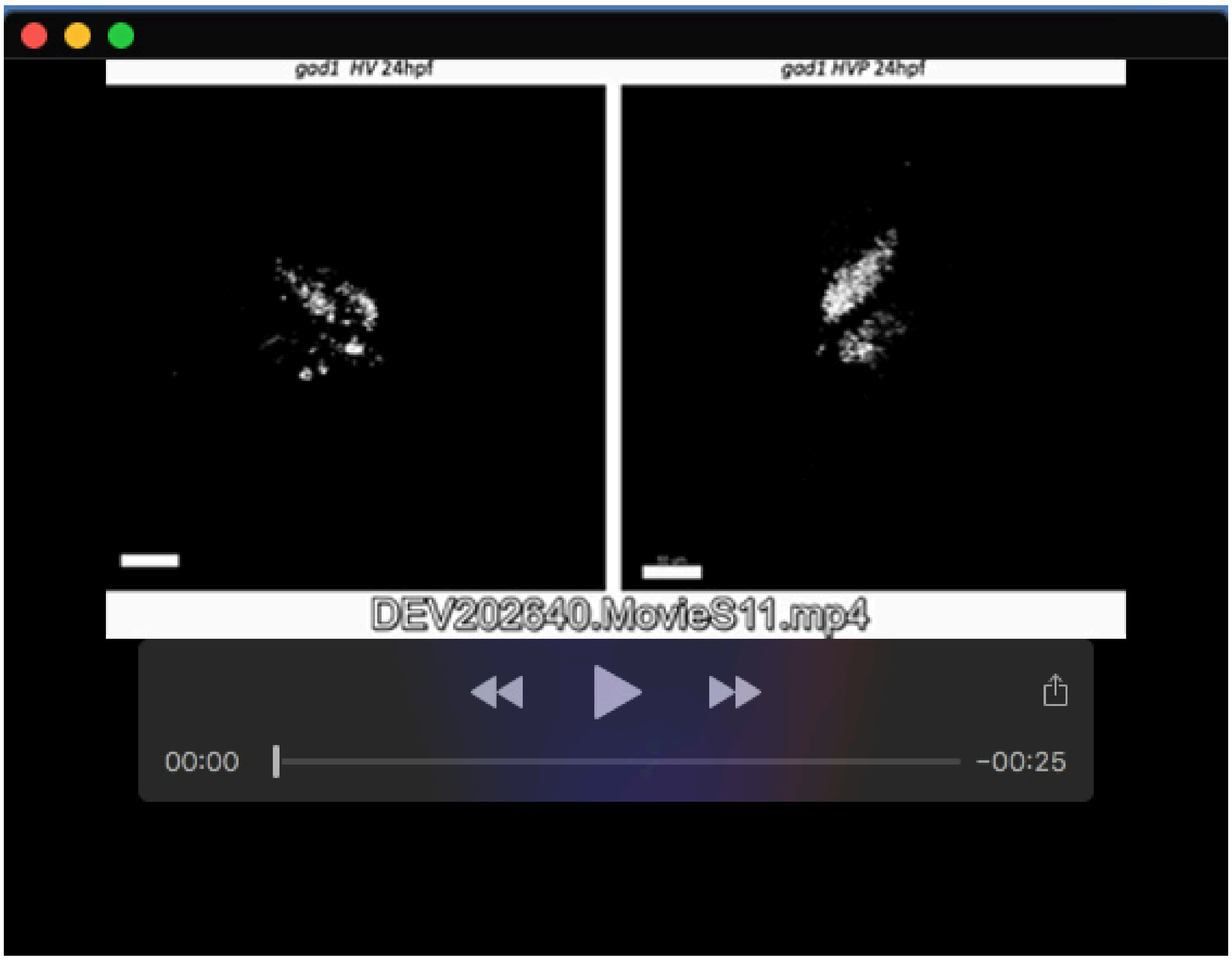

Movie 11. 3D movie of lateral view of the forebrain-telencephalon in *Her6:Venus* (*HV*) and *Her6:Venus-PEST* (*HVP*) embryos at 24hpf, stained for *gad1* using HCR. Scale bar 50µm. Video corresponds to still included in **Fig. 7M-24hpf**.

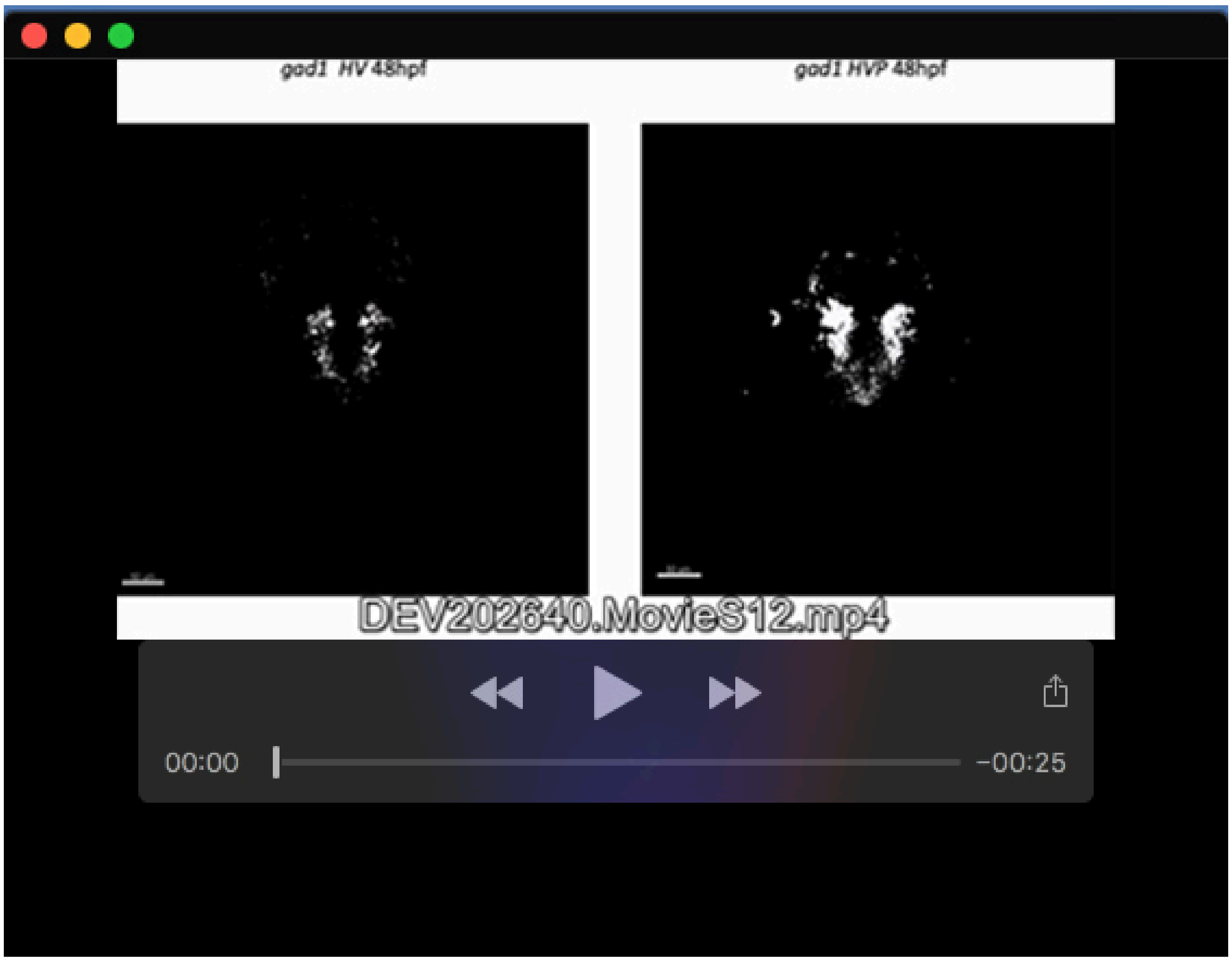

Movie 12. 3D movie of transversal view of the forebrain-telencephalon in *Her6:Venus* (*HV*) and *Her6:Venus-PEST* (*HVP*) embryos at 48hpf, stained for *gad1* using HCR. Scale bar 50µm. Video corresponds to still included in **Fig. 7M-48hpf**.

Development • Supplementary information
