# Supplementary material for: Cell coupling compensates for changes in single-cell Her6 dynamics and provides phenotypic robustness
Source: Development. 2024 May 20;151(10):dev202640. doi: 10.1242/dev.202640 (PMC11190438; doi:10.1242/dev.202640)
Supplement: Supplementary file 1 [file develop-151-202640_review_history.pdf]

Cell coupling compensates for changes in single-cell Her6 dynamics and provides phenotypic robustness

Parnian Doostdar, Joshua Hawley, Kunal Chopra, Elli Marinopoulou, Robert Lea, Kiana Arashvand, Veronica Biga, Nancy Papalopulu and Ximena Soto
DOI: 10.1242/dev.202640

Editor: James Briscoe

Review timeline
Original submission: 9 December 2022
Editorial decision: 9 February 2023
Resubmission: 20 December 2023
Editorial decision: 29 February 2024
First revision received: 3 April 2024
Accepted: 17 April 2024

Original submission

First decision letter

MS ID#: DEVELOP/2022/201508

MS TITLE: Cell coupling compensates for changes in single-cell Her6 dynamics and provides phenotypic robustness

AUTHORS: Parnian Doostdar, Joshua Hawley, Elli Marinopoulou, Robert Lea, Veronica Biga, Nancy Papalopulu, and Ximena Soto

I have now received all the referees reports on the above manuscript, and have reached a decision. The referees' comments are appended below, or you can access them online: please go to BenchPress and click on the 'Manuscripts with Decisions' queue in the Author Area.

As you will see from their reports, the referees recognise the potential of your work, but they also raise significant concerns about it. Given the nature of these concerns, I am afraid I have little choice other than to reject the paper at this stage. The referees question whether the analysis is sufficient to support the conclusion. Experimentally, further evidence would be needed to strengthen the comparison of HV and HVP embryos in order to conclude that there is no early phenotype. In addition, the experiment suggested by referee 1, using Notch inhibition, would test the importance of cell coupling for robustness. The modelling would also need to be better integrated into the study and the concerns raised by Referee 3 addressed.

Having evaluated the paper, I do recognise the potential importance of this work. I would therefore be prepared to consider as a new submission an extension of this study that contains new experiments, data and discussions and that address fully the major concerns of the referees. The work required goes beyond a standard revision of the paper. Please bear in mind that the referees (who may be different from the present reviewers) will assess the novelty of your work in the context of all previous publications, including those published between now and the time of resubmission.

© 2024. Published by The Company of Biologists under the terms of the Creative Commons Attribution License (https://creativecommons.org/licenses/by/4.0/).

Reviewer 1

Advance summary and potential significance to field

The authors analyzed the effect of the protein stability on Her6 expression patterns during telencephalic neurogenesis in zebrafish. They found that destabilization of Her6 protein by adding PEST at the C terminus leads to more variability in the expression levels, increasing the proportion of cells with oscillatory expression and the peak-to-trough fold changes. Furthermore, Her6 expression levels are lower and prematurely decreasing during development, and their correlation between neighboring cells also becomes lower, when the protein is destabilized. However, the telencephalic phenotype is mild and almost normal at an early stage. With mathematical modeling, the authors indicated that cell coupling controls Her6 expression heterogeneity and compensates the brain abnormality caused by destabilization of Her6 protein.

This is an important study for understanding the role of protein stability in gene expression dynamics and the mechanism of a compensation strategy during neural development and of general interest for the readers of the Journal.

Comments for the author

I have the following comments to strengthen the study.

1. Her6:Venus:PEST could be a hypomorphic mutant because the C-terminal WRPW domain, which is important for the repressor activity, might be hidden by Venus-PEST fusion. Thus, the increased Her6 expression heterogeneity observed in the HVP mutant zebrafish could be due to weaker repressor activity rather than due to the protein destabilization. The authors should compare the repressor activity of this mutant with that of wild-type Her6.
2. In HVP mutants, neighboring cells show a weak correlation in expression levels at 20-24 hpf but anti-phase correlation/oscillations at 28 hpf (Figure S6A). It would be informative to include movies of Her6 protein expression and its tracing data of two or more neighboring cells at these stages.
3. The authors indicated that Model 3 with cell coupling is likely to explain the increased Her6 expression heterogeneity of HVP. This is a very important point, and I think that the authors should test this model by comparing Her6 expression patterns in zebrafish embryos in the presence or absence of a Notch inhibitor to see whether heterogeneity is reduced by inhibition of Notch signaling. Alternatively, they can compare Her6 expression patterns between HV and HVP using low-density dissociated cell cultures where cell coupling is absent.
4. It is curious that Her6 protein expression oscillates with 1- to 3-h periodicity in telencephalic progenitors while Her1 protein expression oscillates with a 30-min periodicity during somite segmentation. It would be interesting to discuss why the periodicity is different between Her1 and Her6 or between presomitic mesoderm cells and telencephalic progenitors.

Reviewer 2

Advance summary and potential significance to field

The manuscript entitled ‘Cell coupling compensates for changes in single-cell Her6 dynamics and provides phenotypic robustness’ is using state-of-the-art genome editing approach in zebrafish to assess how changes in protein stability influences oscillatory expression of her6, a member of the core Hes-family transcription factors controlling progenitor division. To follow Hes6 protein over time in zebrafish, they endogenously fuse hes6 ORF with the coding sequence of the fluorescent protein Venus coupled or not with a PEST motif. They show that reducing the stability of Hes6-Venus (HV) protein (by adding PEST domain, HVP) has some substantial impact on Hes6 protein dynamics over time in telencephalic progenitors. Following a rather reduced phenotypic analysis of the HVP embryos they conclude that the changes in protein dynamics do not affect ‘early’ telencephalic development, but they observe an increase in expression of neurotransmitters at much later (48hpf) stages. Although the analysis of Hes6-Venus HV and HVP in these sophisticated lines is of great interest and done rigorously, the subsequent phenotypic analysis of the two lines is rushed and very partial, therefore failing to support the conclusions given. More work analysing telencephalon development in these two lines is therefore required. I am not the best reviewer to

© 2024. Published by The Company of Biologists under the terms of the Creative Commons Attribution License (https://creativecommons.org/licenses/by/4.0/).

comment on the modelling part of the manuscript, but it seems to be too limited to test fully the idea of robustness of population in highly fluctuating individuals.

Comments for the author

Major points:
- Figure 1D shows quite a bit of variability in the FISH detection of her6 mRNA and partial lack of expected co-localisation with pH3 staining. The authors need to show, in the HV homozygous embryos, her6 and venus mRNA staining on DAPI and quantify the level of nuclear expression of both across DV and AP axis of the telencephalon at the stages of interest (I would suggest 5 to 20 somite stage).
- The classification of expression profiles into non-oscillatory and oscillatory in Fig. 3B is by far not as clear as in Fig. 2G. Especially, temporal changes in HV expression does not seem to be convincingly oscillatory in any of the 6 traces (3 deemed 'osc' + 3 'non-osc'). And variation across traces suggest that more exemplar measurements need to be plotted. Difference between the traces shown in 2G and 3B (2G clearly showing oscillation and 3B not really showing any clear ones) for the same HV homozygous line need to be explained.
- In the same vein, the HVP protein expression seems to be always oscillatory. The traces shown in Fig 3B for HVP 'non-oscillatory' show a couple of peaks and troughs more convincing than the 'oscillatory' HV traces in the same figure. Please define very precisely the criteria used to define oscillation and explain how each nucleus is allocated to one or the other category.
- In Fig. 4, the difference in expression level is very convincing between HV and HVP. However, these may be due to change in cell types rather than same cell type expressing differently. And the claim about reduction in number of positive cells is not properly illustrated. The z-projection of the two stacks shown in Figure 4A are not identical with HV sample imaged from tip of olfactory area (anterior-most tip, with skin cells included) and the HVP stack starting deeper (no superficial population and no skin). This difference in volume analysed by itself would introduce a reduction in positive cells in HVP as it does not include the antero-medial her6 high-expressing population. The authors need to show z-projection of whole forebrain in each case. This said, the lateral views (shown in Fig. S3c,d) if it z-projections would strongly support the absence of the anterior-most population. However, this lateral view is of only one z plane.
The data needs to show more than one focal plane to support the statement (difference in depth of the chosen z plane could provide the same difference because the high-expressing population is very medial and can be missed if not at the same z). A set of z planes need to be shown and whole telencephalon (volume including the entire structure) need to be used in the quantification given in Fig. 4 to avoid artificial biases.
- This lack of high-expressing antero-medial population, if verified, may be an exciting phenotype (see next point). A time series needs to be done from 5 to 20 somite stage to see when this difference emerges.
- The absence of impact of the changes in oscillation in HVP on 'early' telencephalic development is not supported by solid evidence. The analysis of the telencephalon is too rudimentary. The authors need to compare dlx2 (subpalium) and tbr1 (pallium) expression (any time between 20 to 24hpf as long as they are sure the stage collected is identical between the two lines) to check possible change in DV identity (suggested by Fig. S8a where ngn1 is expanded in HVP – contradicting the statement in the main text) and use an early olfactory bulb marker (eg. zns2 antibody) to evaluate difference in olfactory bulb development.
The data shown in Fig.S3 suggests there may be an olfactory defect or a more general anterio-posterior issue (Fgf3 ISH at 18 somite stage should be checked too).
- The 'mRNA abundance' shown for neurotransmitters at 24 and 48hpf is poorly explained. Are neuronal domains bigger or cells in same number but expressing higher level of RNA? Smaller comments:
- In zebrafish, her6 is expressed in most/all telencephalic progenitors from 5 to 18 somite stage but seems to restrict progressively to anterior progenitors by 24hpf (ZFIN and Fig. S1). This restriction over-time may cloud some of the analysis done here (eg. colocalization with p-H3 posterior to the anterior-most cells and quantification of expression level across nuclei). Co-localisation with progenitors would have been easier to ascertain at 15-20 somite stage, across the whole telencephalon, while neurogenesis has already started.
- Fig S1 is gorgeous and would have been perfect if adding forebrain frontal. Lateral view makes it is extremely tricky to distinguish progenitors from neurons as they are located on top of each other.

© 2024. Published by The Company of Biologists under the terms of the Creative Commons Attribution License (https://creativecommons.org/licenses/by/4.0/).

- In Fig 6, the telencephalon size measure based on colorimetric staining is prone to interpretation. Use of a fluorescent staining would be more appropriate to calculate telencephalon volume.

Reviewer 3

Advance summary and potential significance to field

In “Cell coupling compensates for changes in single-cell Her6 dynamics and provides phenotypic robustness”, Doostdar and co-authors build a zebrafish line where Her6 is tagged to a Venus florescent protein in order to observe its dynamics (HV), and a line where additionally the Her6 has a faster degradation rate by adding destabilizing PEST domain (HVP). Observing the telencephalon, some changes in HVP dynamics are found with respect to HV. However, phenotypic change is weak, reducing mainly to changes in the late expression of some neuronal differentiation markers. The main criterion for publication of a Research Article or Report in Development is that a paper should make a significant and novel contribution to our understanding of developmental mechanisms. Sincerely, finding that inducing faster degradation changes the dynamics is not surprising, and we do not increase much our understanding on the development of the telencephalon from these observations: this is almost a negative result manuscript (which is fine, and I support publishing in the appropriate venue). The authors claim that the weak phenotype is because cell coupling adds robustness. But, at the same time, they claim that the single cell changes in Her6 dynamics are caused by this cell coupling. Both contradicting claims may be right (or not), but while the first is pure hypothesis, the rationale for the second it comes from a flawed mathematical analysis, as I will detail below. Therefore, I cannot recommend this manuscript for publication in Development.

Comments for the author

About the mathematical analysis: first I have to discuss some of the experimental findings. The authors find that a much lower fraction of the cells in the telencephalon express Venus in the case of PEST mutants, but the variability of the population is greater. A diminishing Venus intensity trend over time is observed in both HV and HVP, and superimposed to it, some cells (around 25% of cells expressing Venus in HV, 70% in HVP) show two cycles of what could be termed “oscillations” (quotation marks because, with only two cycles, we could be talking of a transient phenomenon, although the manuscript does a statistical effort to show that, transient or not, this effect is real; also, I cannot avoid but observing how similar figure 2D is to figure 8E in Schröter et al. PLoS Biology 2012, where the position axis could be translated into time). These oscillations have similar period in HV and HVP, but amplitude is increased in HVP, and, spatially neighboring cells show more uncorrelated fluorescence in HVP (in agreement with the observation of greater variability in the whole population). Of all these observations, the mathematical models only pretend to find a rationale for the increased difference between neighbors in HVP: no comparisons are made to the change in the fraction of oscillators, to the change in the amplitude of the oscillations, or to the equal value of the period. Just for that, I find the analysis rather unsatisfactory.
Note: in the mathematical modeling section the authors freely use the word “exponential” for behaviors for which they give the analytical formula which are actually not exponential, this is a bit disturbing, to say the least.
That said, the manuscript presents three possible models to interpret the experiments. Model 1 is an unregulated gene constitutively expressed. Unsurprisingly, the authors find that this model cannot recapitulate their observations, that involve oscillating gene expression. Model 2 is a negative feedback loop, modeled as a single equation for protein concentration, with no delay of any kind, direct or indirect. Two things can be said about this model: first, I know no evidence of Her6 directly regulating its own expression (none is cited in the manuscript), but for the sake of example I could accept it as a simplified phenomenological model of the dynamics of a more complex network of her genes (although this should be discussed). Second: this model does not fulfill the conditions of the Poincaré-Bendixon theorem: therefore it does not have limit cycle oscillations as solutions. Again, unsurprisingly, it does not reproduce experimental observations: only for very high, unrealistic cooperativity, where damped (not sustained) oscillations of some amplitude may appear, there is a chance of finding some resemblance.
Now, let’s recapitulate. Model 2 is a negative feedback loop with no delay. Model 3 is a negative feedback loop with delay (therefore, allowing for sustained oscillations)… together with delayed

© 2024. Published by The Company of Biologists under the terms of the Creative Commons Attribution License (https://creativecommons.org/licenses/by/4.0/).

intercellular coupling. This model has some properties, when increasing the degradation rate, that compare better with the increased difference between neighbors in HVP. This leads the authors to the conclude "that the increase in population Her6 expression heterogeneity is an emergent property of finely tuned Notch signalling coupling between single cells". However, Model 3 is not the next logical step in complexity from Model 2: that would be a negative feedback loop with delay, a model that could sustain oscillations. It is against this delayed model that Model 3 should have been tested: Model 2 without delay is a straw man, and claims that isolated cell dynamics cannot reproduce the dynamics are unsubstantiated without having studied the relevant single cell model. There is yet another model, simpler than Model 3, that also makes sense testing: a model only with lateral inhibition. This, however, attending to the sentence "our 2-cell model (Model 3) fails to recapitulate the HVP phenotype when it is only run in the presence of lateral inhibition but without autoinhibition" has been tested, although no further information is given.
Finally, the hypothesis that "cell coupling compensates for changes in single-cell Her6 dynamics and provides phenotypic robustness" is just that, a hypothesis with no support by any experimental or modeling data.
Moreover, it is in contrast with the the conclusion that cell coupling is the responsible for the increase in population Her6 expression heterogeneity. Other hypothesis are equally compelling (and untested), just to cite one, it is possible that Her6 is forming a network of post-transcriptional regulations with other Her proteins that somehow mitigates the phenotypic effect of changes in the dynamics of just one element of the network.
Summarizing: I find the single cell and tissue dynamics reported alluring, but the mathematical modeling flawed and the conclusions unsupported. Under this conditions, I cannot recommend publication in Development. The mathematical modeling would be helpful if analyzing a logically complete set of models to really exclude single cell phenomena as an explanation, while at the same time comparing with a larger set of experimental observations (fraction of oscillating cells; amplitude, not only relative, but also absolute, of oscillations). Also, there is previous work on dynamics of Her6 in relation to other Her genes that could be used to inform the model. This is such an amount of work that it goes beyond the scope of a revision, leaving for two possibilities: publish the experimental findings and leave the theory for a follow up; or keep it all together in a broadly different version of the manuscript. Also note that neither modeling nor experiments support the title. Experimentally, disruption of Notch signaling is a possibility to test this hypothesis.

First revision

Author response to reviewers' comments

We would like to thank the reviewers for the thorough review of our manuscript and their constructive comments. We have taken great care to address the vast majority of the comments, we have performed additional experiments and in certain places, we have gone over and above what we have been asked to do. As a result, we are pleased to say that our paper has been greatly improved and the support for our conclusions is now stronger. Below we provide a detailed point-by-point response to the reviewers comments.

Reviewer 1

Advance Summary and Potential Significance to Field:
The authors analyzed the effect of the protein stability on Her6 expression patterns during telencephalic neurogenesis in zebrafish. They found that destabilization of Her6 protein by adding PEST at the C terminus leads to more variability in the expression levels, increasing the proportion of cells with oscillatory expression and the peak-to-trough fold changes.
Furthermore, Her6 expression levels are lower and prematurely decreasing during development, and their correlation between neighboring cells also becomes lower, when the protein is destabilized. However, the telencephalic phenotype is mild and almost normal at an early stage. With mathematical modeling, the authors indicated that cell coupling controls Her6 expression heterogeneity and compensates the brain abnormality caused by destabilization of Her6 protein.

© 2024. Published by The Company of Biologists under the terms of the Creative Commons Attribution License (https://creativecommons.org/licenses/by/4.0/).

This is an important study for understanding the role of protein stability in gene expression dynamics and the mechanism of a compensation strategy during neural development and of general interest for the readers of the Journal.

Thank you for the accurate summary and your supportive comments.

Reviewer 1 Comments for the Author:
I have the following comments to strengthen the study.

1. Her6:Venus:PEST could be a hypomorphic mutant because the C-terminal WRPW domain, which is important for the repressor activity, might be hidden by Venus-PEST fusion. Thus, the increased Her6 expression heterogeneity observed in the HVP mutant zebrafish could be due to weaker repressor activity rather than due to the protein destabilization. The authors should compare the repressor activity of this mutant with that of wild-type Her6.

We have addressed this comment in several ways. Firstly, we have looked at the level of expression of downstream targets of Her6, such as the proneural Ngn and Ascl1a. No significant difference at their level of expression was detected, suggesting that the transcriptional repression activity of Her6 has not been affected. Her6 is negatively auto-repressed but the abundance of her6 mRNA also does not change overall. These new data have been added in Fig. 7 (for ngn1 and ascl1a mRNA) and Fig. S5D (for her6 mRNA).

Secondly, the new multicellular, coupled, mathematical modelling shows that the Her6 expression heterogeneity into low expressing and high expressing cells can be reproduced by differences in protein stability that are aligned to the experimental values (new Fig. 5). Thirdly, although not new data, we have verified the effect of PEST-Venus fusion on Her6 protein stability (Fig. S4A-D).

Finally, in our own previous work we have used and thoroughly characterised the Her6 Venus fusion reporter. A Venus fusion reporter has also been used before in zebrafish somitogenesis by Delaune at al, 2012 (https://doi.org/10.1016/j.devcel.2012.09.00) and a Venus-PEST domain fusion (has been used to destabilise Lfng to image Lfng oscillations in the mouse (Aulehla et al., 2008 DOI: 10.1038/ncb1679). These references are included in the manuscript and add confidence to our methodology.

Nevertheless, we can't totally exclude some other subtle effect on protein function, and we now make this clear in the discussion on page 14.

2. In HVP mutants, neighboring cells show a weak correlation in expression levels at 20-24 hpf but anti-phase correlation/oscillations at 28 hpf (Figure S6A). It would be informative to include movies of Her6 protein expression and its tracing data of two or more neighboring cells at these stages.

Thank you for raising this point which gives an opportunity to add a clarification. The correlations now shown in Figure S6A,B are all correlations in expression levels not in the phase of oscillations. Thus, the reduction in correlation shown at 28hpf refers to levels of expression between neighbouring cells. Cells from HVP embryos are less correlated at all time points (20, 24, 28hpf) and this difference increases at 28hpf (shown with values in Figure S6A).

We have included some example traces of Her6 protein expression in neighbouring cells from live movies of HVP mutants, in new Figure S6C, as requested. It can be seen that in HVP the level of expression is very different between neighbouring cells, but the phase is variable and sometimes neighbouring cells are in phase. A complete phase analysis in the tissue is outside the scope of this study and it is probably a paper in its own right (as we have done in the mouse spinal cord, see Biga et al., 2021. Mol Syst Biol).

3. The authors indicated that Model 3 with cell coupling is likely to explain the increased Her6 expression heterogeneity of HVP. This is a very important point, and I think that the authors should test this model by comparing Her6 expression patterns in zebrafish embryos in the presence or absence of a Notch inhibitor to see whether heterogeneity is reduced by inhibition of Notch

© 2024. Published by The Company of Biologists under the terms of the Creative Commons Attribution License (https://creativecommons.org/licenses/by/4.0/).

signaling. Alternatively, they can compare Her6 expression patterns between HV and HVP using low- density dissociated cell cultures where cell coupling is absent.

This was an excellent suggestion and one that we have taken fully on board. In the revised manuscript, we have compared Her6 heterogeneity of neighbouring cells in embryos expressing the Her6-Venus-PEST fusion (HVP), with and without inhibition of Notch (by DAPT and DMSO respectively). We found that the increase in heterogeneity observed in neighbouring cells of HVP embryos is reduced when Notch signalling is reduced. This finding supports our concept that cell-cell coupling via Notch is responsible for the increased heterogeneity when Her6 protein is destabilised. These new results are included in Figure 4 new panels H-J and Figure S6 new panel E.

In addition, we have improved Model 3 so that it is now fully a coupled multicellular model rather than 2 interacting cells and we were pleased to see that it supports our experimental interpretations. For the new modelling results see new Figure 5 and Figure S7.

4. It is curious that Her6 protein expression oscillates with 1- to 3-h periodicity in telencephalic progenitors while Her1 protein expression oscillates with a 30-min periodicity during somite segmentation. It would be interesting to discuss why the periodicity is different between Her1 and Her6 or between presomitic mesoderm cells and telencephalic progenitors.

This is an interesting difference which could be due to differences in cell-cell coupling parameters and time delays in the 2 tissues. As Julian Lewis has shown for her1/7 in 2003, (DOI: 10.1016/s0960-9822(03)00534-7), such differences can elongate the periodicity of Her protein oscillations. We made a note of this on page 8 where the Figure 2 results are presented.

Comment for the reviewer only: As this is an interesting point, we have looked into the reported periodicity of Her1 in the somite segmentation in some detail. It is true that Delaune et al 2012 (https://doi.org/10.1016/j.devcel.2012.09.00) reported a periodicity of approx. 30 mins for Her1 protein reporter in somitogenesis and this reference is now included in the text. However, the recent Rohde et al biorxiv https://www.biorxiv.org/content/10.1101/2021.05.29.446196v2.full article from Andy Oates's lab reports that Her1 period is variable, increases as differentiation proceeds and at its highest amplitude it is approx. 1.2h in the embryo and 1.3h in-vitro (panel J in the attached figure). This is within our range of reported periodicity so perhaps the situation is not very different. In addition, there appears to be some meaningful variability which is outside the scope of this paper.

NOTE: Figure provided for reviewer has been removed. It showed Figure 1 from Rohde, L. A., Bercowsky-Rama, A., Valentin, G., Naganathan, S. R., Desai, R. A., Strnad, P., Soroldoni, D. and Oates, A. C. (2023). Cell-autonomous timing drives the vertebrate segmentation clock's wave pattern. *bioRxiv* doi:10.1101/2021.05.29.446196. We have removed unpublished data that had been provided for the referees in confidence.

Reviewer 2

Advance Summary and Potential Significance to Field:

The manuscript entitled 'Cell coupling compensates for changes in single-cell Her6 dynamics and provides phenotypic robustness' is using state-of-the-art genome editing approach in zebrafish to assess how changes in protein stability influences oscillatory expression of her6, a member of the core Hes-family transcription factors controlling progenitor division. To follow Hes6 protein over time in zebrafish, they endogenously fuse hes6 ORF with the coding sequence of the fluorescent protein Venus coupled or not with a PEST motif. They show that reducing the stability of Hes6-Venus (HV) protein (by adding PEST domain, HVP) has some substantial impact on Hes6 protein dynamics over time in telencephalic progenitors. Following a rather reduced phenotypic

© 2024. Published by The Company of Biologists under the terms of the Creative Commons Attribution License (https://creativecommons.org/licenses/by/4.0/).

analysis of the HVP embryos they conclude that the changes in protein dynamics do not affect ‘early’ telencephalic development, but they observe an increase in expression of neurotransmitters at much later (48hpf) stages. Although the analysis of Hes6- Venus HV and HVP in these sophisticated lines is of great interest and done rigorously, the subsequent phenotypic analysis of the two lines is rushed and very partial, therefore failing to support the conclusions given. More work analysing telencephalon development in these two lines is therefore required. I am not the best reviewer to comment on the modelling part of the manuscript, but it seems to be too limited to test fully the idea of robustness of population in highly fluctuating individuals.

Thank you for your supporting comments and you fair criticisms. We have deepened the phenotypic analysis as outlined in detail below. Overall, the new analysis supports our initial conclusions.

Reviewer 2 Comments for the Author:
Major points:
-Figure 1D shows quite a bit of variability in the FISH detection of her6 mRNA and partial lack of expected co-localisation with pH3 staining.

Some variability is not unexpected in snapshots of a gene with dynamic gene expression. We have added colocalization of her6 expression, pH3 staining and DAPI in Figure 1E. These data show that the co-localisation of pH3 with her6 is indeed partial; pH3 staining is confined to the apical zone, close to the ventricle, while her6 expression includes medial pH3 and more lateral non-pH3 stained cells. This is very much in line with our previous findings. Specifically, in Soto et al., 2020, we reported her6 is expressed in apical progenitor cells but also in cells that are in the process of transitioning to differentiation (where it is co-expressed with Elavl3) and is switched off in fully differentiated cells. This is also what we have previously found for the mouse Hes5 (Manning et al., 2019).
The authors need to show, in the HV homozygous embryos, her6 and venus mRNA staining on DAPI and quantify the level of nuclear expression of both across DV and AP axis of the telencephalon at the stages of interest (I would suggest 5 to 20 somite stage).

In Figure 1B, we included FISH for her6 mRNA and venus protein in WT and HV Homozygous embryos to show overlap in forebrain tissue staining. In Figure 1C we also show FISH for her6 mRNA and venus mRNA in HV Homozygous embryos, again showing overlap. All are maximum intensity projections and we have made the labelling clearer. In the revised manuscript we also show 3D rotations of the staining which should give a complete view of the expression (movie S1). We are not sure what extra information, that would be relevant to this paper, the requested analysis would provide. Therefore, with apologies, we have focused on experiments that were key to strengthening our conclusions.

-The classification of expression profiles into non-oscillatory and oscillatory in Fig. 3B is by far not as clear as in Fig. 2G. Especially, temporal changes in HV expression does not seem to be convincingly oscillatory in any of the 6 traces (3 deemed 'osc' + 3 'non-osc'). And variation across traces suggest that more exemplar measurements need to be plotted. Difference between the traces shown in 2G and 3B (2G clearly showing oscillation and 3B not really showing any clear ones) for the same HV homozygous line need to be explained.

-In the same vein, the HVP protein expression seems to be always oscillatory. The traces shown in Fig 3B for HVP ‘non-oscillatory’ show a couple of peaks and troughs more convincing than the ‘oscillatory’ HV traces in the same figure. Please

© 2024. Published by The Company of Biologists under the terms of the Creative Commons Attribution License (https://creativecommons.org/licenses/by/4.0/).

define very precisely the criteria used to define oscillation and explain how each nucleus is allocated to one or the other category.

Thank you for giving us the opportunity to explain the method that we use. We do not classify traces as oscillatory or not based on visual inspection. We use a statistical method based on Gaussian Processes and model fitting that we published as Phillips et al 2017 for luminescence data and adapted in Soto et al. 2020 for Her6 fluorescence data. Briefly, the her6-Venus traces are normalised by division to H2B-Keima traces that are not oscillatory (do not pass the test, see Figure 2J and Figure 3F, H2B-mKeima), to remove any technical artefacts. The traces are then detrended, as long-range trends (3 times the expected periodicity) interfere with the detection of periodic activity. These steps are detailed in Figure 2D and G . Then our detection method is applied which involves a model fitting approach, essentially looking at the likelihood that the given traces have been generated by an oscillatory of non-oscillatory process. The method is described in detail in the Materials and Methods- “ **Detection of oscillatory and aperiodic fluctuating activity from time-series” section**. More example traces and their classification are shown in Figure S2.

In the revised manuscript, in order to increase the reader’s confidence in the detection of oscillations, we have analysed the traces with an additional method that uses frequency analysis to quantify the “coherence’ of the dynamic traces. High coherence (close to 1) means better oscillations. This is now included in the Figure 3G and described in the Materials and Methods- **“Coherence of Power Spectra**” section. This independent method agrees that the HVP traces are more “coherent” i.e. more oscillatory (as shown previously in Figure 3F).

We have improved Figure 3B by showing 2 example traces (rather than 3 which were difficult to separate) and by changing the colour so it is easier to distinguish each trace. Please note however, that indeed, in HVP embryos the Her6 traces are overall more dynamic we do see a big increase in cells that show oscillatory expression as shown in Figure 2F as part of the HVP phenotype. In the revised manuscript, we were pleased that our new mathematical modelling data (added in new Figure 5) can also explain the increase in the % of oscillators as we elaborate in our response to reviewer 3.

Finally, Figure 2C (time series image) has been replaced with a less processed image where the example traced cells and the fluctuations in Her6 intensity are easier to see. We have not been asked to make this last change, but we have proactively decided that it will make visual evaluation of the dynamicity of the trace easier.

-In Fig. 4, the difference in expression level is very convincing between HV and HVP. However, these may be due to change in cell types rather than same cell type expressing differently.

And the claim about reduction in number of positive cells is not properly illustrated.

The reduction in the number of positive cells is shown in Figure 4A and it is quantitated in Figure 4B with additional repeats in Figure S5A. It is clear that the her6 expression domain is smaller in HVP embryos (now included as Figure S5C). However, the interspersed “salt and pepper” appearance of positive and negative cells in the HVP embryos (shown in Figure 4A) argues against a specific cell type missing and more in favour of increased heterogeneity in the level of expression and an overall reduction in the Her6 domain. There is also no reduction in the overall size of the telencephahon at 20 or 24hpf even though her6 is already quite abnormal (see new Foxg1 staining in Figure 6).
The z-projection of the two stacks shown in Figure 4A are not identical with HV sample imaged from tip of olfactory area (anterior-most tip, with skin cells included) and the HVP stack starting deeper (no superficial population and no skin).
This difference in volume analysed by itself would introduce a reduction in positive cells in HVP as it does not include the antero-medial her6 high-expressing population.
The authors need to show z-projection of whole forebrain in each case.

This said, the lateral views (shown in Fig. S3c,d) if it z-projections would strongly support the absence of the anterior-most population. However, this lateral view is of only one z plane.

© 2024. Published by The Company of Biologists under the terms of the Creative Commons Attribution License (https://creativecommons.org/licenses/by/4.0/).

The data needs to show more than one focal plane to support the statement (difference in depth of the chosen z plane could provide the same difference because the high-expressing population is very medial and can be missed if not at the same z). A set of z planes need to be shown and whole telencephalon (volume including the entire structure) need to be used in the quantification given in Fig. 4 to avoid artificial biases.

-This lack of high-expressing antero-medial population, if verified, may be an exciting phenotype (see next point). A time series needs to be done from 5 to 20 somite stage to see when this difference emerges.

We have taken great care to match the z-stacks shown for Her6-venus in Figure 4A , based on the shape of anatomical landmarks (in this case Olfactory placodes, now labelled OP in Figure 4A) and the shape of the Forebrain cross section. However, the H2B-mKeima Caax RFP panel has escaped our vigilance and indeed the reviewer is right that the HV stack shown is more anterior that HVP. We have now replaced this with a stack that starts at a comparable level. However, we must stress that H2B-mKeima/Caax RFP panel was purely illustrative of the methodology used to identify nuclei/cells. The Her6-venus panels in Figure 4A are all comparable z-projections of the whole forebrain.

Additionally, we have now added 3D rotations of the forebrain in the new supplementary Movie S1. One can see the generalised reduction in Her6 level but also increased heterogeneity in Her6 expression (Figure 4) and increase in oscillatory activity (Figure 3).

-The absence of impact of the changes in oscillation in HVP on 'early' telencephalic development is not supported by solid evidence. The analysis of the telencephalon is too rudimentary. The authors need to compare dlx2 (subpalium) and tbr1 (pallium) expression (any time between 20 to 24hpf as long as they are sure the stage collected is identical between the two lines) to check possible change in DV identity (suggested by Fig. S8a where ngn1 is expanded in HVP – contradicting the statement in the main text)

The issue with normal FISH could give high background and give misleading results and use an early olfactory bulb marker (eg. zns2 antibody) to evaluate difference in olfactory bulb development.
The data shown in Fig.S3 suggests there may be an olfactory defect or a more general anterio-posterior issue (Fgf3 ISH at 18 somite stage should be checked too).
We have improved the telencephalic phenotype characterisation as follows: we have used a different in situ method based on Hybridisation Chain reaction (HCR) and we have performed quantification/volume measurements normalised to Foxg1 and backed it up by qPCR. We included early progenitor/neuronal markers (elavl3, ngn1, ascl1), subpallial and pallial neuronal markers (vglut2a, tbr1, gad1, gad2) all in early and late stages and comparing HV and HVP. This is included in the new Figure 7. Overall, the new experimentation supports our conclusion that there is no significantly different early phenotype but some markers can be abnormal (although the overall size of the telencephalon is normal) at late stages.

The previous data that showed the telencephalic phenotype based on double FISH for elavl3/ascl1/ngn1, plus quantification, is now in supplementary Figure S8F-I with her6 volume and RT-qPCR quantification moved to Figure S5C,D. It is true that there is some variability in the expression of these markers between embryos, for example in Figure S8H ngn1 staining may look increased, as the reviewer pointed out. However, this is not reproducible and there are not statistically significant differences. We suspect that tissue-level compensation for premature decline in Her6 is not perfect. Later in development, there are some small differences when using terminally differentiated markers, which we interpret as compensatory mechanisms having reached the limit of what they can do to preserve normal development.

© 2024. Published by The Company of Biologists under the terms of the Creative Commons Attribution License (https://creativecommons.org/licenses/by/4.0/).

We understand the scepticism for what an unusual finding is but we hope that the additional experimentation and data analysis work has strengthened our interpretation.

-The ‘mRNA abundance’ shown for neurotransmitters at 24 and 48hpf is poorly explained. Are neuronal domains bigger or cells in same number but expressing higher level of RNA?

Our new HCR data for gad1 suggest that both are true i.e. the neuronal domain is bigger, and cells express higher level of RNA. (shown in new Figure 7M).

Smaller comments:

-In zebrafish, her6 is expressed in most/all telencephalic progenitors from 5 to 18 somite stage but seems to restrict progressively to anterior progenitors by 24hpf (ZFIN and Fig. S1). This restriction over-time may cloud some of the analysis done here (eg. colocalization with p-H3 posterior to the anterior-most cells and quantification of expression level across nuclei). Co-localisation with progenitors would have been easier to ascertain at 15-20 somite stage, across the whole telencephalon, while neurogenesis has already started.

We agree that there is gradual restriction of Her6 which is also evident in our Figure 4A and 4B that shows the evolution of expression at 20hpf, 24hpf and 26hpf. This gradual restriction during development is ALSO observed in HVP embryos, although more dramatic as cell lose Her6 expression, again shown in Figure 4A and B. The main difference between HV and HVP, at any stage of development analysed, is the increased heterogeneity in the level of Her6 expression in neighbouring cells in HVP embryos. This is shown in Figure 4A (we have added arrows to show some high expressing cells in HVP) and thoroughly quantitated in 3D throughout the paper.

-Fig S1 is gorgeous and would have been perfect if adding forebrain frontal. Lateral view makes it is extremely tricky to distinguish progenitors from neurons as they are located on top of each other.

We are glad that the reviewer liked this time-course of expression figure. We are unable to re-do the entire time course on a frontal view and we don’t think that it would add a substantial amount of new information. The new PH3/Her6 co- localisation data that we have added in Figure 1E, together with the rest of the current and previous data, agree that her6 overlaps partially with PH3 on one hand, and partially with elavl3 on the other. This is consistent with Her6 expression occurring in dividing progenitors, but continuing as these progenitors transition to differentiation and is eventually switching off when cells differentiate (see also Soto et al., 2020).

-In Fig 6, the telencephalon size measure based on colorimetric staining is prone to interpretation. Use of a fluorescent staining would be more appropriate to calculate telencephalon volume.

We have repeated this experiment with Fluorescent in Situ hybridisation (Hybridisation Chain Reaction; HCR) as requested and we measured the volume in 3D images. The result was remarkably the same as with the colorimetric in situ’s, i.e. that there is non-significant difference in volume early (24hpf), a small difference at 30hpf, and no difference at 48hpf, indicating that any small early differences in the overall size of the telencephalon are compensated during the course development. We have kept the chromogenic ISH data in Figure 6A-C and added the new fluorescent in situ data in Figure 6D-F, as they tell the same story but with different methods.

Reviewer 3

Advance Summary and Potential Significance to Field:

In “Cell coupling compensates for changes in single-cell Her6 dynamics and provides phenotypic

© 2024. Published by The Company of Biologists under the terms of the Creative Commons Attribution License (https://creativecommons.org/licenses/by/4.0/).

robustness", Doostdar and co-authors build a zebrafish line where Her6 is tagged to a Venus florescent protein in order to observe its dynamics (HV), and a line where additionally the Her6 has a faster degradation rate by adding destabilizing PEST domain (HVP). Observing the telencephalon, some changes in HVP dynamics are found with respect to HV. However, phenotypic change is weak, reducing mainly to changes in the late expression of some neuronal differentiation markers. The main criterion for publication of a Research Article or Report in Development is that a paper should make a significant and novel contribution to our understanding of developmental mechanisms. Sincerely, finding that inducing faster degradation changes the dynamics is not surprising, and we do not increase much our understanding on the development of the telencephalon from these observations: this is almost a negative result manuscript (which is fine, and I support publishing in the appropriate venue). The authors claim that the weak phenotype is because cell coupling adds robustness. But, at the same time, they claim that the single cell changes in Her6 dynamics are caused by this cell coupling. Both contradicting claims may be right (or not), but while the first is pure hypothesis, the rationale for the second it comes from a flawed mathematical analysis, as I will detail below. Therefore, I cannot recommend this manuscript for publication in Development.

To provide stronger support for the interpretation of our findings we have added new experiments (notably, the effect of blocking cell coupling by Notch inhibition) and we have significantly deepened the mathematical analysis as outlined below, proactively going beyond what was requested. We hope that the new results add confidence to our interpretation and that the reviewer now appreciates the originality of our paper which does not simply report a negative result. Our paper shows that cell coupling can provides tissue level compensation for single cell dynamic changes, the latter brought about by changes in biochemical parameters such as protein stability. We are not aware of similar work in the literature.

Reviewer 3 Comments for the Author:
About the mathematical analysis: first I have to discuss some of the experimental findings. The authors find that a much lower fraction of the cells in the telencephalon express Venus in the case of PEST mutants, but the variability of the population is greater. A diminishing Venus intensity trend over time is observed in both HV and HVP, and superimposed to it, some cells (around 25% of cells expressing Venus in HV, 70% in HVP) show two cycles of what could be termed "oscillations" (quotation marks because, with only two cycles, we could be talking of a transient phenomenon, although the manuscript does a statistical effort to show that, transient or not, this effect is real; also, I cannot avoid but observing how similar figure 2D is to figure 8E in Schröter et al. PLoS Biology 2012, where the position axis could be translated into time).

These oscillations have similar period in HV and HVP, but amplitude is increased in HVP, and, spatially neighboring cells show more uncorrelated fluorescence in HVP (in agreement with the observation of greater variability in the whole population). Of all these observations, the mathematical models only pretend to find a rationale for the increased difference between neighbors in HVP: no comparisons are made to the change in the fraction of oscillators, to the change in the amplitude of the oscillations, or to the equal value of the period. Just for that, I find the analysis rather unsatisfactory.

In the previous manuscript, we had analysed the outcome of a two-cell coupled model (model 3 in the previous manuscript) building up from simpler models. While the reviewer did not directly complain about model 3, we felt that modelling the interaction of just 2 cells was a limitation of our work, as more than 2 cells interact in the tissue in vivo. In addition, we have new experimental data that show that disruption of Notch signalling rescues the Her6 expression level heterogeneity- included in Figure 4H-J and Figure S6E.

In the revised manuscript, using this new multicellular model (new Model 2; Figure 5B), we were able to examine additional dynamic features, as requested by the reviewer. Specifically, we examined theoretically the increase in the fraction of oscillators (average coherence, Figure 5F), the change in the amplitude (time series coefficient of variation Figure 5D) and the period, which was found to the same in HV and HVP (Figure 5E), for small changes in protein degradation values. This new mathematical analysis shows that we can accurately reproduce the differences we observe at the single cell level in HV versus HVP tissue.

Note: in the mathematical modeling section the authors freely use the word "exponential" for

© 2024. Published by The Company of Biologists under the terms of the Creative Commons Attribution License (https://creativecommons.org/licenses/by/4.0/).

behaviors for which they give the analytical formula which are actually not exponential, this is a bit disturbing, to say the least.

Using the term exponential was indeed a mistake and we should have used the term asymptotic instead; we apologise for this error. The term was used to describe Models 1 and 2 which have now been removed in favour of more complete models, therefore, this term has also been completely removed from the text.

That said, the manuscript presents three possible models to interpret the experiments. Model 1 is an unregulated gene constitutively expressed. Unsurprisingly, the authors find that this model cannot recapitulate their observations, that involve oscillating gene expression. Model 2 is a negative feedback loop, modeled as a single equation for protein concentration, with no delay of any kind, direct or indirect. Two things can be said about this model: first, I know no evidence of Her6 directly regulating its own expression (none is cited in the manuscript), but for the sake of example I could accept it as a simplified phenomenological model of the dynamics of a more complex network of her genes (although this should be discussed).

Second: this model does not fulfill the conditions of the Poincaré-Bendixon theorem: therefore it does not have limit cycle oscillations as solutions. Again, unsurprisingly, it does not reproduce experimental observations: only for very high, unrealistic cooperativity, where damped (not sustained) oscillations of some amplitude may appear, there is a chance of finding some resemblance.

Now, let's recapitulate. Model 2 is a negative feedback loop with no delay. Model 3 is a negative feedback loop with delay (therefore, allowing for sustained oscillations)… together with delayed intercellular coupling. This model has some properties, when increasing the degradation rate, that compare better with the increased difference between neighbors in HVP. This leads the authors to the conclude "that the increase in population Her6 expression heterogeneity is an emergent property of finely tuned Notch signalling coupling between single cells". However, Model 3 is not the next logical step in complexity from Model 2: that would be a negative feedback loop with delay, a model that could sustain oscillations.

It is against this delayed model that Model 3 should have been tested: Model 2 without delay is a straw man, and claims that isolated cell dynamics cannot reproduce the dynamics are unsubstantiated without having studied the relevant single cell model. There is yet another model, simpler than Model 3, that also makes sense testing: a model only with lateral inhibition. This, however, attending to the sentence "our 2-cell model (Model 3) fails to recapitulate the HVP phenotype when it is only run in the presence of lateral inhibition but without autoinhibition" has been tested, although no further information is given.

We will reply to these comments together, because we have made drastic improvement to this part of the paper. In the revised manuscript, we have reformulated the set of models and we have removed models 1 (constitutively expressed gene) and 2 (negative feedback loop with no delay) which were too simplistic to be useful, even as "straw men".

We are now starting directly from a multicellular model of uncoupled single cells, modelled with stochastic delay differential equations (new Model 1; Figure 5A), capable of generating single cell oscillations via protein repressing its own expression of the form used in (Monk, 2003)). This uncoupled cell model has now been more fully described and discussed in the results section, again finding that it is insufficient in recapitulating the HVP increase in heterogeneity. Then, we compare this with a coupled Her6 expression in a multicellular context, again with stochastic delay differential equations (new Model 2; Figure 5B). Both of the new models do satisfy the Poincaré-Bendixon theorem. Developing the coupling model further was also motivated by our new experimental data where disruption of Notch signalling rescues the protein destabilisation population heterogeneity, suggesting that cell-cell coupling is crucial. We were very pleased to see that this new coupled multicellular Her6 model recapitulated the Her6 abundance heterogeneity and bimodal distribution when we vary the protein degradation rate by small values, which themselves align with the experimental data. This model also allowed us to look at the effect in the fraction of oscillations, the period and amplitude, as mentioned above. This is now shown in new Figure 5.

© 2024. Published by The Company of Biologists under the terms of the Creative Commons Attribution License (https://creativecommons.org/licenses/by/4.0/).

With regards to the comment of the evidence of Her6 autoregulating itself, we can say the following: Autoinhibition of Hes genes has been first shown in the mouse for Hes1 and Hes7 by Kageyama's lab (Hirata et al., 2002; DOI: 10.1126/science.1074560) (Bessho et al., 2003, DOI: 10.1101/gad.1092303) respectively. Her6 is the zebrafish orthologue of the mouse Hes1 therefore we also expect autorepression to underlie the oscillatory activity. Additionally, autorepression has been shown for the zebrafish her1 (Brend and Holley, 2009 DOI: 10.1002/dvdy.22100) and has been incorporated in most , if not all, models of Hes/Her oscillatory activity, for example, in the zebrafish, see Lewis, 2003, DOI: 10.1016/s0960-9822(03)00534-7 Giudicelli et al., 2007 DOI: 10.1371/journal.pbio.0050150.) for Her1 and Her7. We have now added a section in the introduction regarding autorepression and included the references above.

Finally, the hypothesis that "cell coupling compensates for changes in single-cell Her6 dynamics and provides phenotypic robustness" is just that, a hypothesis with no support by any experimental or modeling data.

Moreover, it is in contrast with the the conclusion that cell coupling is the responsible for the increase in population Her6 expression heterogeneity. Other hypothesis are equally compelling (and untested), just to cite one, it is possible that Her6 is forming a network of post-transcriptional regulations with other Her proteins that somehow mitigates the phenotypic effect of changes in the dynamics of just one element of the network.

The combination of new modelling (described here) and disruption of Notch signalling experiments (described in response to reviewer 1, point 3) provide much stronger support for our hypothesis, which can be summarised as follows: Cell coupling is able to compensate for the premature loss of her6 protein in some cells when protein degradation is increased. This is manifested by upregulating Her6 protein in other cells, and results in increased heterogeneity in Her6 expression at the population level. We propose that this compensation of the level of gene expression in some cells, account for the minor, if any, phenotypic differences between the wild type and the mutant embryos, providing some level of phenotypic robustness in the face of biochemical parameter changes, especially in early stages of development.

Summarizing: I find the single cell and tissue dynamics reported alluring, but the mathematical modeling flawed and the conclusions unsupported. Under this conditions, I cannot recommend publication in Development. The mathematical modeling would be helpful if analyzing a logically complete set of models to really exclude single cell phenomena as an explanation, while at the same time comparing with a larger set of experimental observations (fraction of oscillating cells; amplitude, not only relative, but also absolute, of oscillations). Also, there is previous work on dynamics of Her6 in relation to other Her genes that could be used to inform the model. This is such an amount of work that it goes beyond the scope of a revision, leaving for two possibilities: publish the experimental findings and leave the theory for a follow up; or keep it all together in a broadly different version of the manuscript. Also note that neither modeling nor experiments support the title. Experimentally, disruption of Notch signaling is a possibility to test this hypothesis.

We hope the reviewer will appreciate the depth and thoroughness of our revisions, including both modelling and experimental data, even though the amount of work may have exceeded what would be expected from a revision.

Resubmission

First decision letter

MS ID#: DEVELOP/2023/202640

© 2024. Published by The Company of Biologists under the terms of the Creative Commons Attribution License (https://creativecommons.org/licenses/by/4.0/).

MS TITLE: Cell coupling compensates for changes in single-cell Her6 dynamics and provides phenotypic robustness

AUTHORS: Parnian Doostdar, Joshua Hawley, Kunal Chopra, Elli Marinopoulou, Robert Lea, Kiana Arashvand, Veronica Biga, Nancy Papalopulu, and Ximena Soto

Apologies for the length of time that the review process has taken. I was keen that the paper was seen by the same referees that saw it in the first iteration, and I'm afraid one of the referees had several deadlines early in the year that delayed their review. But I have now received all the referees' reports on the above manuscript, and have reached a decision. The referees' comments are appended below, or you can access them online: please go to BenchPress and click on the 'Manuscripts with Decisions' queue in the Author Area.

As you will see, the referees express considerable interest in your work and recognise that most of the initial concerns have been addressed, but Referee 2 still has some significant criticisms and recommends a revision of your manuscript before we can consider publication. Referee 2 argues the manuscript does not sufficiently engage with prior work on Her6 dynamics and interactions with other Her proteins, omitting a key reference (SchrÃ¶ter 2012), which provides evidence seemingly inconsistent with assumptions made in the current modelling approach. While appreciating the study's advance, the Referee is of the opinion that findings from SchrÃ¶ter et al 2012 need to be addressed to strengthen the theoretical framing and accurately position the findings within the current understanding of the system.

If you are able to revise the manuscript along the lines suggested, I will be happy to receive a revised version of the manuscript. Your revised paper will be re-reviewed by one or more of the original referees, and acceptance of your manuscript will depend on your addressing satisfactorily the reviewers' major concerns. Please also note that Development will normally permit only one round of major revision. If it would be helpful, you are welcome to contact us to discuss your revision in greater detail. Please send us a point-by-point response indicating your plans for addressing the referees' comments, and we will look over this and provide further guidance.

Please attend to all of the reviewers' comments and ensure that you clearly highlight all changes made in the revised manuscript. Please avoid using 'Tracked changes' in Word files as these are lost in PDF conversion. I should be grateful if you would also provide a point-by-point response detailing how you have dealt with the points raised by the reviewers in the 'Response to Reviewers' box. If you do not agree with any of their criticisms or suggestions please explain clearly why this is so.

Reviewer 1

Advance summary and potential significance to field

The authors have properly addressed my concerns.

Comments for the author

I have no further comments.

Reviewer 2

Advance summary and potential significance to field

I have had the opportunity to review the revised version of the manuscript titled "Cell coupling compensates for changes in single-cell Her6 dynamics and provides phenotypic robustness," by Parnian Doostdar et al. I commend the authors for their diligent work and the improvements made to the manuscript based on previous feedback. The effort to enhance both the experimental and theoretical aspects of the study is evident and addresses several concerns raised during the initial review process. The investigation into the role of Her6 in the embryonic zebrafish telencephalon and its implications for phenotypic robustness is both ambitious and timely. The use of a

© 2024. Published by The Company of Biologists under the terms of the Creative Commons Attribution License (https://creativecommons.org/licenses/by/4.0/).

homozygote endogenous Her6:Venus reporter and 4D single-cell tracking to elucidate the dynamics of Her6 oscillation and expression is particularly noteworthy and advances our understanding of cellular behavior in developmental biology.

Comments for the author

However, despite these strengths, a crucial issue regarding the incorporation and discussion of relevant previous works remains inadequately addressed. Specifically, the manuscript does not sufficiently engage with earlier studies on Her6 and its interactions with other Her family members, which are essential for a comprehensive understanding of Her6 dynamics and function. For example, in situ experiments on the zebrafish segmentation clock have shown non-oscillatory expression of her6 mRNA, differing from the cyclic expression observed for her1 or her7 mRNA. This is within the context of the presomitic mesoderm (PSM), that of course could be different in the telencephalon, but warrants discussion to contextualize the current findings within the broader research landscape.

Moreover, the omission of a key reference, Schröter 2012, which specifically examines the molecular interactions between Her1, Her7, and Her6 in zebrafish represents a significant gap in the literature review. Schröter 2012's findings on the binding capacities and dimerization patterns of these proteins provide crucial insights into their regulatory mechanisms and interactions, which are directly relevant to the current study's focus on Her6 dynamics. This work found evidence for promiscuous formation of all pairs of Her Protains homo- and heterodimers, but only the Her1 homodimer and Her6/Her7 heteordimer showed strong DNA binding to promnoters of her1, her7 or deltaC. The manuscript's modeling component, which interprets experimental evidence without considering these known interactions, and posing Her6 auto-inhibition, appears at odds with established evidence. The failure to discuss these precedents compromises the manuscript's theoretical foundation, both conceptually and mathematically. However, Schröter 2012 did find evidence for Her6 protein oscillation, as a byproduct of forming dimers with Her1 and Her7, cyclic even at the mRNA level. It may be possible to reconcile these observations with the ones presented in this manuscript, and it definitely has to be discussed.

To justify the auto-inhibitory activity of Her6, the manuscript cites works that predate Schröter 2012 and primarily concern Her1 and Her7. This reliance on older references to support assumptions about Her6 dynamics undermines the argument's contemporary relevance and accuracy.

I appreciate the stated intention behind the presented model as a qualitative tool for elucidating mechanisms. Nonetheless, the interpretation of experimental results heavily depends on assumptions that diverge from existing evidence. A more thorough integration of this evidence into the manuscript's narrative is crucial for a balanced and accurate presentation of the work. Addressing this gap would not only strengthen the manuscript's theoretical basis but also enhance its contribution to the field by situating its findings within a broader context of known protein interactions and dynamics.

On a minor note, the stochastic model formulation appears sound, and the inclusion of dynamic snapshots or figures illustrating the model's predicted behaviors over time would enhance the reader's understanding. Additionally, the minimal difference in degradation rates observed in the model, 10%, despite being qualitatively consistent with the experimental design, warrants a brief discussion regarding its implications for the study's conclusions.

In summary, while the manuscript presents significant advancements in our understanding of Her6 dynamics and phenotypic robustness in the zebrafish telencephalon, addressing the aforementioned concerns regarding literature integration and theoretical framing is essential for providing a comprehensive and accurate account of the study's contributions to the field. Incorporating these suggestions would not only clarify the manuscript's position within the existing body of research but also strengthen its scientific impact.

Reference:

© 2024. Published by The Company of Biologists under the terms of the Creative Commons Attribution License (https://creativecommons.org/licenses/by/4.0/).

Schröter C, Ares S, Morelli LG, Isakova A, Hens K, et al. (2012) Topology and Dynamics of the Zebrafish Segmentation Clock Core Circuit. PLoS Biol 10(7): e1001364. doi:10.1371/journal.pbio.1001364

First revision

Author response to reviewers' comments

Reviewer 2 Advance Summary and Potential Significance to Field:
I have had the opportunity to review the revised version of the manuscript titled "Cell coupling compensates for changes in single-cell Her6 dynamics and provides phenotypic robustness," by Parnian Doostdar et al. I commend the authors for their diligent work and the improvements made to the manuscript based on previous feedback. The effort to enhance both the experimental and theoretical aspects of the study is evident and addresses several concerns raised during the initial review process. The investigation into the role of Her6 in the embryonic zebrafish telencephalon and its implications for phenotypic robustness is both ambitious and timely. The use of a homozygote endogenous Her6:Venus reporter and 4D single-cell tracking to elucidate the dynamics of Her6 oscillation and expression is particularly noteworthy and advances our understanding of cellular behavior in developmental biology.

We thank the reviewer for their supportive comments.

Reviewer 2 Comments for the Author:
However, despite these strengths, a crucial issue regarding the incorporation and discussion of relevant previous works remains inadequately addressed. Specifically, the manuscript does not sufficiently engage with earlier studies on Her6 and its interactions with other Her family members, which are essential for a comprehensive understanding of Her6 dynamics and function. For example, in situ experiments on the zebrafish segmentation clock have shown non-oscillatory expression of her6 mRNA, differing from the cyclic expression observed for her1 or her7 mRNA. This is within the context of the presomitic mesoderm (PSM), that of course could be different in the telencephalon, but warrants discussion to contextualize the current findings within the broader research landscape.

Unfortunately, there appears to have been some confusion with regards to the gene nomenclature. The non-oscillatory family member that the reviewer refers to, and which is described in the Schroter, C. et al., 2012 paper, is Hes6, not Her6.

Her6 which we describe in our work is the ortholog of the mammalian Hes1, as we specify in the introduction (line 108). Hes1/Her6 is distinct from Hes6. The ZFIN ID number for Her6 is ZDB-GENE-980526-144 while the ZFIN ID for Hes6 is ZDB-GENE-030828-5. We have added the ID number of Her6 in the materials and methods (under molecular cloning) to avoid any such confusion for other readers of our paper. In the discussion (line 462-470), we now also do state that Her6 is distinct from Hes6.

This is a significant difference because Hes6, which is described in the Schroter, C. et al., 2012 paper, is an unusual member of the Hairy and enhancer of split related (HES/Her) family of transcriptional repressors. Hes6 alone does not bind DNA and perhaps this is why it does not oscillate at mRNA level. In addition, it suppresses Hes1 (the ortholog of Her6) preventing it from repressing transcription. Furthermore, Hes6 is unusual because rather than inhibiting neuronal differentiation like “canonical” Hes/Her genes, it promotes neuronal differentiation, most likely by inhibiting Hes1, as Kageyama’s lab have shown in Bae, S-K. et al., 2000 (DOI: 10.1242/dev.127.13.2933). Hes6 was also described in mouse and frog at the same time by the Kintner lab (Koyano-Nakagawa, N. et al., 2000) again showing that it has the unusual property of promoting, rather than inhibiting, neuronal differentiation. Kintner and colleagues suggested that Hes6 acts in a positive loop with neurogenins (DOI: 10.1242/dev.127.19.4203). Hes6 is not induced by Notch, and it is expressed in both progenitors and neurons, while canonical Hes/Her members are expressed in progenitors and switch off in neurons.

© 2024. Published by The Company of Biologists under the terms of the Creative Commons Attribution License (https://creativecommons.org/licenses/by/4.0/).

Subsequently, the Schroter, C. et al., 2012 paper showed that Hes6 heterodimerises with other Her family members, namely Her1 and Her7, and forms complexes with strong DNA binding affinity but only with Her7. This heterodimerisation affects the "effective stability" of Hes6 (page 11 of their paper) so that it oscillates at the protein level even though it does not oscillate at the mRNA level.

To summarise, Hes6 (Schroter, C. et al., 2012) is very different than Her6, the zebrafish ortholog of Hes1 (this work). The interactions with other family members that have been shown for Hes6, have not been studied for Her6. Hes1 (the human ortholog of Her6) is known to oscillate at the mRNA level to auto-repress itself (references in the paper, lines 82-86).

Moreover, the omission of a key reference, Schröter 2012, which specifically examines the molecular interactions between Her1, Her7, and Her6 in zebrafish, represents a significant gap in the literature review. Schröter 2012's findings on the binding capacities and dimerization patterns of these proteins provide crucial insights into their regulatory mechanisms and interactions, which are directly relevant to the current study's focus on Her6 dynamics. This work found evidence for promiscuous formation of all pairs of Her Protains homo- and heterodimers, but only the Her1 homodimer and Her6/Her7 heteordimer showed strong DNA binding to promnoters of her1, her7 or deltaC. The manuscript's modeling component, which interprets experimental evidence without considering these known interactions, and posing Her6 auto-inhibition, appears at odds with established evidence. The failure to discuss these precedents compromises the manuscript's theoretical foundation, both conceptually and mathematically. However, Schröter 2012 did find evidence for Her6 protein oscillation, as a byproduct of forming dimers with Her1 and Her7, cyclic even at the mRNA level. It may be possible to reconcile these observations with the ones presented in this manuscript, and it definitely has to be discussed.

To justify the auto-inhibitory activity of Her6, the manuscript cites works that predate Schröter 2012 and primarily concern Her1 and Her7. This reliance on older references to support assumptions about Her6 dynamics undermines the argument's contemporary relevance and accuracy.

I appreciate the stated intention behind the presented model as a qualitative tool for elucidating mechanisms. Nonetheless, the interpretation of experimental results heavily depends on assumptions that diverge from existing evidence. A more thorough integration of this evidence into the manuscript's narrative is crucial for a balanced and accurate presentation of the work. Addressing this gap would not only strengthen the manuscript's theoretical basis but also enhance its contribution to the field by situating its findings within a broader context of known protein interactions and dynamics.

As mentioned above the Schroter, C. et al., 2012 paper deals with Hes6, not Her6, which are different genes products, with very different biological effects, protein properties and mode of regulation, as fully explained above.

The paper of Schroter, C. et al., 2012 is concerned with developing a mathematical model that is sufficient to explain the phenotypes that they observe in somitogenesis in single and double Her1 and Her7 mutants. The central idea of their model is Hes/Her proteins dimerize promiscuously and form a dimer cloud that contains complexes with strong and weak DNA binding affinity. In their paper they show that Hes6 (which as we explained above is different than Her6) heterodimerises with Her1 and Her7, has a destabilising effect on both, but forms heterodimers with strong binding affinity only with Her7. In the absence of Her7, more Hes6 dimerises with Her1 reducing the pool of Her1 homodimers that are available for transcriptional regulation. This dimer sequestration mechanism was a novel idea for the control of the segmentation clock. While these are interesting ideas, there is no experimental evidence related to homo or hetero dimer formation of Her6 (either in this paper or prior literature) and thus, we would not be able to introduce such interactions in our model because they would not be backed up by experimental data.

In the addition to the differences highlighted above, one other important difference of the Schroter, C. et al., 2012 paper from ours is that it deals with single cells, while we focus on the cell interactions that occur in a multicellular environment. The Schroter, C. et al. paper specifically states "*Therefore, although our single cell model allows us to fit a range of dynamic tissue-level*

© 2024. Published by The Company of Biologists under the terms of the Creative Commons Attribution License (https://creativecommons.org/licenses/by/4.0/).

phenotypes, it is possible that these phenotypes do not solely arise from changes in the regulatory interactions within single cells but might also depend on the modulation of altered single cell dynamics by cell-cell communication.

To summarise, the Schroter, C. et al. 2012 paper is outside the context of our work for the following reasons;

It deals with Hes6, not Her6, which have different properties, as explained in detail above. It deals with somitogenesis, not neurogenesis which has very different tissue formation requirements. It deals with complete protein knock-outs rather than a subtle change in protein stability. It deals with a single cell model while the focus of our paper is specifically the multicellular interactions.

Our multicellular mathematical model shows that the difference in Her6 dynamics that we observe can be explained by coupling of cells and reduced stability of the protein. Thus, we do not need to evoke a more complicated scenario to explain our experimental observations. Nevertheless, in the interests of highlighting the complexity of Hes/Her protein regulation, in the re-revised manuscript we are discussing that hetero and homo dimer formation are biochemical control processes of Hes/Her protein regulation. As such, they may have a role to play in the phenotype and they ought to be taken into consideration in the interpretation of molecular manipulations of any individual Hes/her protein in future work. This can be found in lines 464-470 of the revised paper.

On a minor note, the stochastic model formulation appears sound, and the inclusion of dynamic snapshots or figures illustrating the model's predicted behaviors over time would enhance the reader's understanding.

We have included the behaviour over time in Figure 5, which shows the time traces of all cells in each simulation for each degradation rate.

Additionally, the minimal difference in degradation rates observed in the model, 10%, despite being qualitatively consistent with the experimental design, warrants a brief discussion regarding its implications for the study's conclusions.

We have previously discussed this in lines 409-412, as follows “The addition of a PEST domain had a small effect in the overall stability of the protein, which was revealed when the protein was introduced in a heterologous system (a mammalian cell line); yet, the effect on the protein dynamics was pronounced”. We have now added a sentence, as follows; “The implication of our work is that small differences in protein stability at the single cell level (in the order of 10%) can have a strong effect on protein dynamics at population level when the cells are coupled” (lines 412-414).

In summary, while the manuscript presents significant advancements in our understanding of Her6 dynamics and phenotypic robustness in the zebrafish telencephalon, addressing the aforementioned concerns regarding literature integration and theoretical framing is essential for providing a comprehensive and accurate account of the study's contributions to the field. Incorporating these suggestions would not only clarify the manuscript's position within the existing body of research but also strengthen its scientific impact.

We hope that our additional changes to the manuscript have addressed these concerns.

Reference:
Schröter C, Ares S, Morelli LG, Isakova A, Hens K, et al. (2012) Topology and Dynamics of the Zebrafish Segmentation Clock Core Circuit. PLoS Biol 10(7):e1001364. doi:10.1371/journal.pbio.1001364

We have included the citation to the Schroter, C. et al., 2012 paper.

Additional revisions

© 2024. Published by The Company of Biologists under the terms of the Creative Commons Attribution License (https://creativecommons.org/licenses/by/4.0/).

We have made two additional changes to the manuscript, which we outline here.

1. Line 156; corrected the word “co-localisation” between her6 and elavl3” to “partial overlap of the domains of expression” to avoid the impression of analysis at single cell level.

2. Upon proof-reading of the manuscript we realised that there was an error in Figure 7. Specifically, there was a mismatch between the statistical method mentioned in Table S6, where the statistical methods for all figures are summarised, and the statistical method actually used in the analysis of the RT-PCR data shown in Figure7.

While Table S6 specifies that the test used was “ANOVA with Sidak multiple comparison correction”, the data shown in Figure 7 had been analysed with a non-parametric unpaired T-test in R. The error arose when inadvertently we used an interim version of this figure. While using a t-test is not “wrong” as such, the ANOVA test is preferable in this figure because it is a more complete test and with it, the statistical analysis of Figure 7 would be consistent with the statistical analysis of other figures in the paper. Therefore, we have now replaced the affected panels as shown below. We must emphasize that the raw data is exactly the same and will be available to the reader as the source data; the graphs themselves have not changed at all. The only difference is that the P value is now precisely specified by the number of stars (as indicated in Table S6) and that Tbr1 went from small change to non-significant change. Thus, the biological conclusion that any changes in marker gene expression between HV and HVP are confined to some terminal differentiation markers and at late stages of development has not changed.

In Supplementary Table S6 we now also specify 1-way versus 2-way ANOVA, as appropriate.

Fig 7 at previous resubmission stage

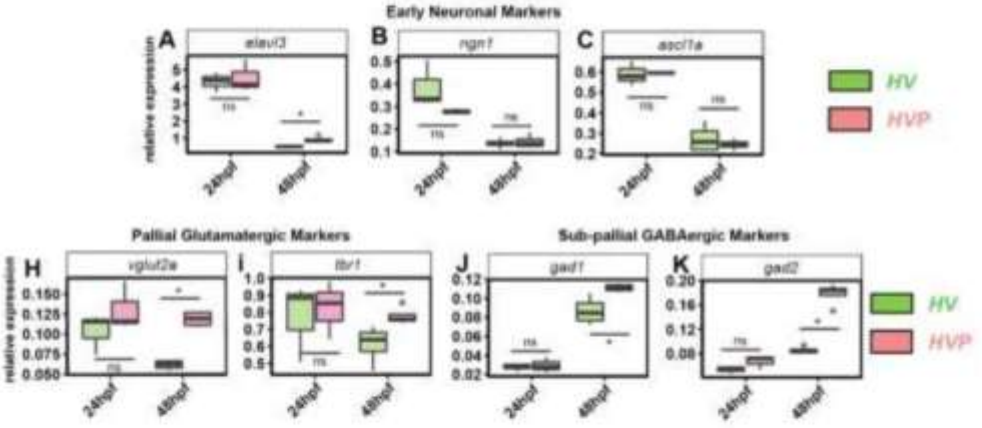

Fig 7 after amendments in current manuscript

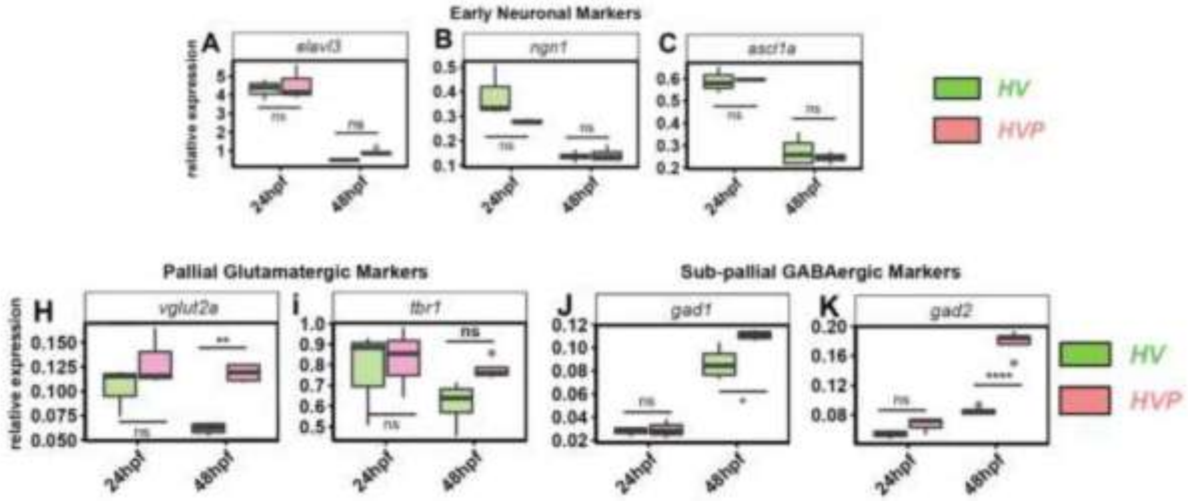

© 2024. Published by The Company of Biologists under the terms of the Creative Commons Attribution License (https://creativecommons.org/licenses/by/4.0/).

Second decision letter

MS ID#: DEVELOP/2023/202640

MS TITLE: Cell coupling compensates for changes in single-cell Her6 dynamics and provides phenotypic robustness

AUTHORS: Parnian Doostdar, Joshua Hawley, Kunal Chopra, Elli Marinopoulou, Robert Lea, Kiana Arashvand, Veronica Biga, Nancy Papalopulu, and Ximena Soto
ARTICLE TYPE: Research Article

I am happy to tell you that your manuscript has been accepted for publication in Development, pending our standard ethics checks.

Reviewer 2

Advance summary and potential significance to field

The responses are satisfactory, and I recommend publication of the manuscript.

Comments for the author

I apologize to the authors for the confusion with the gene's name. I should indeed do an effort to stay up to date with the nomenclature. Thank you for the detailed explanations. I also appreciate the effort to explain these nomenclature details in the new version, to avoid that any reader can be confused as I was.

Congratulations to the authors on the nice job.

© 2024. Published by The Company of Biologists under the terms of the Creative Commons Attribution License (https://creativecommons.org/licenses/by/4.0/).
